# The ChUSE trial - improving access to psychological therapies for children with distressing sensory experiences in NHS CAMHS: a randomised controlled feasibility trial protocol

**DOI:** 10.1136/bmjopen-2026-119727

**Published:** 2026-09-10

**Authors:** Sarah Parry, Sarah Carr, Lesley-Anne Carter, Sue Kirk, Cinzia Petrussa, Fiona Malpass, Gemma E Shields, Alexander Thompson, Anthony Morrision, Filippo Varese

**Affiliations:** 1 Young People’s Mental Health Research Centre, Pennine Care NHS Foundation Trust, Ashton-under-Lyne, UK; 2 Division of Psychology and Mental Health | School of Health Sciences | Faculty of Biology, The University of Manchester, Manchester, UK; 3 Complex Trauma and Resilience Research Unit, Greater Manchester Mental Health NHS Foundation Trust, Manchester, UK; 4 Centre for Biostatistics, Division of Population Health, Health Services Research and Primary Care, The University of Manchester, Manchester, UK; 5 School of Health Sciences, The University of Manchester, Manchester, UK; 6 Mind in Camden, Voice Collective, London, UK; 7 Centre for Health Economics, The University of Manchester, Manchester, UK; 8 Manchester Centre for Health Economics, The University of Manchester, Manchester, UK; 9 Division of Psychology and Mental Health, The University of Manchester, Manchester, UK; 10 Psychosis Research Unit, Greater Manchester Mental Health NHS Foundation Trust, Manchester, UK

**Keywords:** MENTAL HEALTH, Child & adolescent psychiatry, Psychosocial Intervention, Clinical Protocols

## Abstract

**Introduction:**

Distressing sensory experiences (DSEs) are commonly reported by children and young people accessing Child and Adolescent Mental Health Services (CAMHS) in the UK. Despite their prevalence, evidence-based tailored interventions for this population are scarce. The ChUSE trial aims to address this gap by evaluating the feasibility and acceptability of a novel brief talking therapy for children aged 8–16 years and an accompanying parent coaching programme.

**Methods and analysis:**

A single-blind, two-arm randomised controlled feasibility trial will be conducted across three National Health Service trusts in Greater Manchester, UK. Sixty young people under the care of CAMHS experiencing DSEs and their parents/caregivers will be randomised 1:1 to receive either treatment as usual (TAU) or TAU plus the ChUSE intervention. The intervention involves four child therapy sessions and three optional parent sessions. Primary feasibility outcomes include recruitment and retention rates, treatment adherence and intervention safety. Secondary objectives assess the feasibility of data collection procedures and gather qualitative insights into trial acceptability and the influence of the intervention on young people’s well-being.

**Ethics and dissemination:**

The ChUSE Trial has received ethical approval through the Health Research Authority following review by the West Midlands – South Birmingham Research Ethics Committee specialising in paediatric research (ID 327343). The feasibility trial addresses a significant clinical gap in CAMHS provision and will also capture information as to current TAU practices, contributing novel data to guide future service provision as TAU is currently understood to be heterogeneous. The information gathered through this trial will inform the design of a definitive trial evaluating the clinical effectiveness and cost-consequences of an early, age-appropriate intervention for DSEs. Findings and translational outputs will be shared through scientific and public forums to reach a variety of audiences.

**Trial registration number:**

ISRCTN12245618.

**STRENGTHS AND LIMITATIONS OF THIS STUDY**

- Rigorous methodological design with clearly defined procedures that support transparency and replicability.
- Systematic recruitment and sampling approach to minimise selection bias and enhance the credibility of the findings.
- Use of validated data-collection tools, increasing reliability and consistency of measurement, paired with more novel and specialised measures to explore any phenomenological change.
- Potential limitations in generalisability, as the sample is suitably small, so it is unlikely to be representative of all relevant populations or contexts.
- Risk of unmeasured confounding variables, which may influence interpretations despite efforts to control methodological bias.

## Introduction

Approximately half of children and young people (CYP) attending Child and Adolescent Mental Health Services (CAMHS) report distressing sensory experiences (DSEs^1^), including auditory and visual hallucinations, feelings of disconnection and intrusive sensory thoughts.^2 3^ Young people can experience DSEs due to the chronic stress of poverty^4^, bereavement, abuse and neglect,^5 6^ bullying,^7^ medical conditions such as epilepsy,^8^ adverse responses to medication^9^ or disrupted sleep.^10^ Distressing voice content has been associated with negative self-schemas and insecure attachments with caregivers.^10^ Voice hearing is often transient in childhood and not usually a sign of severe mental illness, with DSEs (also referred to as multimodal hallucinations) typically less complex and less distressing in non-clinical samples.^11 12^ However, young people who experience even transient voice-hearing can report reduced quality of life at later follow-ups compared with their peers^13^ and a higher rate of suicidal ideation,^14^ highlighting the potential long-term impact of voice hearing. Furthermore, DSEs in adolescence have been connected to later experiences of depression and anxiety,^15^ indicating a need for intervention as soon as possible to promote coping, well-being and to prevent further difficulties.

A large-scale review^16^ and primary research^17 18^ indicate that many children begin to report unusual sensory experiences (USEs) between the ages of 8 and 11 years, with 51% of a community sample describing these experiences as distressing.^19^ From the age of 14, some young people may access specialist support through psychosis care pathways in the UK’s National Health Service (NHS). However, referrals to such services can generate additional anxieties, potentially increasing self-stigma and adversely affecting well-being.^20^ Current referral pathways for psychosis are not appropriate for many children experiencing DSEs, as only 15–22% of young people identified as at-risk for developing psychosis will access these pathways within a year.^21^ This misalignment in referral pathways can increase anxiety for families and delay access to proportionate support in age-appropriate child and adolescent settings.

Providing earlier, non-psychosis-specific support within CAMHS could improve timely access to appropriate, proportionate interventions while reducing future reliance on specialist services. Early intervention is crucial to promote coping strategies,^22^ enhance well-being^15^ and to potentially mitigate the development of more severe conditions such as psychosis or mood disorders throughout the turbulent teenage years and into early adulthood.

Auditory verbal hallucinations are thought to be the most common multisensory experience, although this assumption is largely based on data with adults and young people aged 16 years and over. Voices can be comforting or distressing, and young people can experience both comforting and distressing voices at the same time,^23^ each sometimes taking on distinct identities.^16 24 25^ Visual and tactile hallucinations can occur separately or concurrently and have been linked to trauma and/or anxiety.^26 27^ The impact of these multisensory experiences varies, influenced by individual psychosocial factors, personal resources and social support.^28 29^ Despite nearly 50% of children in CAMHS reporting multisensory experiences,^1^ there is currently no evidence-based talking therapy available for children with DSEs.

Young people have described benefitting from re-appraisal, normalising,^30^ psychoeducation, externalisation,^1^ multisensory and cognitive restructuring coping strategies,^17 19^ similar to many of the approaches within cognitive therapies and compassionate mind training (CMT). Emerging findings suggest compassion for voices is a beneficial approach for young adult voice hearers.^31–33^ Our recent reviews have explored what is particularly important for young people with DSEs and first episode psychosis in therapeutic relationships,^34^ specific gender needs for girls when help-seeking,^35^ family members' experiences of help-seeking for DSEs,^36^ and compassionate approaches to coping with voice hearing for young people.^37^ These reviews recommend enhancing early support and communication about DSEs for young people, highlighting that girls and young women may experience more dismissive responses to their difficulties when they present to services and the importance of collaborative therapeutic relationships and compassion-informed approaches for coping with hearing voices.

Drawing from the adult literature, a recent feasibility study on electronic or digital Cognitive Behavioral Therapy (e-CBT) for adults with DSEs found 4–6 sessions were acceptable for service users and progression criteria were met for recruitment, retention and adherence to treatment.^38^ While it is positive to see the development of new interventions among the adult population,^39^ there remains a need for DSE research to be undertaken with young people in the unique setting of CAMHS, employing age-appropriate interventions.^16^ Suitably tailored youth-focused interventions are limited in their availability and scientific evidence-base due to a scarcity of research with young people and their families to inform tailored protocols. Overall, research has overlooked younger children with DSEs, which means important developmental and contextual information is currently missing from our understanding of how children experience DSEs and what support they and their families want from services.

Thus far, we know further work is needed with younger children to improve treatment pathways and reduce delays in accessing suitable support and that clinicians desire clearer clinical guidance for tailored care.^16^ Priorities for tailored care seem to include careful, developmentally informed assessment and caution around the use of structured interviews, which may insufficiently capture the form, function and content of DSEs.^40^ Detailed clinical assessment is also important to distinguish between DSEs and hallucinations caused by neurological conditions such as migraine and occipital epilepsy.^41^ Due to the role traumatic events^40^,^42^, substance use, sleep disorders, depression and anxiety may play in the experience of DSEs,^42^ a trauma-informed assessment may also be helpful where appropriate support can then be offered. Additionally, DSEs have been associated with sensory processing difficulties in paediatric samples for many years, although causal relationships and treatment implications remain unclear.^43^ The ChUSE Trial is open to children in core CAMHS, autistic children and children currently being assessed for neurodiversity in CAMHS. Adopting a neuroinclusive approach as early as possible in the treatment development process helps to ensure the final intervention is more acceptable and useful for all.

Developmental processes, including executive functioning, social cognition and language development, are thought to influence DSE phenomenology,^44^ although further research with younger children is needed to understand how to inform psychological support for DSEs. Overall, further research is needed to design developmentally sensitive assessments, interventions and treatment strategies and to improve psychosocial outcomes.^45^ Drawing on this emerging literature base and alongside lived experience consultations between 2016 and 2025, the ChUSE Trial aims to address recommendations for care in CAMHS by offering young people and their parents/caregivers an intervention that mobilises existent knowledge to offer a tailored brief coping-focused therapy, while assessing feasibility parameters to prepare for a definitive trial.

When supporting younger children with DSEs, it is essential to consider the role and involvement of parents and caregivers. Parental exclusion within services and feelings of shame and guilt can contribute towards relational instability within families.^46^ Family interventions for psychosis (FIfP) have been found to improve cognitive functioning, reduce symptoms of psychosis and reduce the likelihood of relapse.^47^ Importantly, carer burden and well-being can also improve following a FIfP approach, indicating benefits for the wider family system of parental support, which may be especially relevant for younger children. The parent coaching sessions within the ChUSE trial will offer a new and tailored approach for parents, offering separate spaces with a tailored therapeutic focus for parent and child.

In a recent review of parents’ experiences of seeking help for young people with DSEs, we found many parents cannot find suitable care or are deterred from help-seeking following finding ‘nothing on the other side of the door’ of children’s mental health services for DSEs, which can delay help-finding until the young person is in crisis.^36^ Our primary research with parents and caregivers prior to the ChUSE trial demonstrated parents want reliable information, skills and strategies to help their child cope.^23^ Prior research identified that many families have dissatisfying experiences of help-seeking for hearing voices and would like more opportunities for normalising and holistic care.^48^ Therefore, the ChUSE Trial meets an identified need in CAMHS, for children, practitioners and parents alike, offering a symptom-focused, low-intensity, systemic early intervention, adding significantly to the clinical support for children and their parents and caregivers.

### The ChUSE trial

A significant challenge in treating DSEs for young people is the absence of child-centred research informing clinical guidelines, despite the recognised prevalence of these difficulties within CAMHS.^1^ CAMHS practitioners frequently report missing the clinical guidance necessary to support this group of young people, resulting in restricted treatment options and delays in care for young people presenting with DSEs. To address this, the ChUSE trial will implement and evaluate an intervention protocol designed to offer early intervention for use within CAMHS. In time, this could provide practitioners with clear, practical guidance and ensure timely, appropriate support for young people. Informed by evidence from cognitive-behaviour therapy (CBT) interventions for adults and the few studies available that have involved CYP, the ChUSE therapy integrates psychoeducation and therapeutic mechanisms from CBT and CMT to formulate young people’s experiences of DSEs and identify potential coping strategies to reduce distress and enhance coping for each young person.

A challenge for the ChUSE Trial is our limited understanding of what constitutes ‘treatment as usual’ (TAU) for this population. This adds complexity to the design and delivery of high-quality research in this area. To mitigate this, the ChUSE trial will incorporate a TAU arm, systematically gathering data on existing care practices across three NHS trusts. This will provide essential information to inform the design of a future definitive trial.

## Methods and analysis

### Aims and objectives

#### Aim

To evaluate the feasibility and acceptability of conducting a future, larger-scale, definitive trial to assess the clinical effectiveness and cost-consequences of a brief psychological intervention for young people aged 8–16 years experiencing DSEs, accompanied by up to three parent-coaching sessions.

#### Objectives

The primary objective is to assess key feasibility outcomes and gather data to inform the design of a future multi-site randomised controlled trial (RCT). Specifically, the study will examine:

- The feasibility of recruiting children and their parents or caregivers to the trial.
- The retention of participants throughout the trial period.
- Engagement with and adherence to the intervention and trial procedures.
- The safety of the intervention and trial procedures.
- Estimation of key parameters to support a sample size calculation for a future definitive efficacy trial.

#### Secondary objectives

In addition to the primary feasibility outcomes, this study will address the following feasibility uncertainties:

- To determine the uptake of the optional parent coaching sessions.
- To evaluate the feasibility of collecting a set of proposed outcome measures, informing the selection of appropriate measures and data collection procedures for a future trial.
- To gather qualitative data exploring the acceptability of the intervention and trial procedures from the perspectives of young people, parents and caregivers, as well as their recommendations for refining the trial design.
- To collect qualitative insights from CAMHS practitioners and clinical leads regarding barriers, facilitators, costs and consequences of implementing the intervention within routine CAMHS provision.

#### Primary feasibility outcomes

The main feasibility outcomes for this study are as follows:

- Recruitment rates, measured as the number of family units recruited and randomised per month.
- Participant retention rates, expressed as the proportion of participants completing outcome assessments at each time point.
- Treatment engagement and adherence, assessed by the number of sessions attended by young people and parents or caregivers allocated to both the ChUSE intervention plus TAU arm and the TAU-only arm.
- Treatment fidelity, determined by the extent to which trial therapists deliver the ChUSE intervention according to the intervention protocol, as measured by fidelity checklists.
- Safety of the intervention, evaluated through the monitoring and recording of adverse events (AEs) and serious adverse events (SAEs), both related and unrelated to the intervention or trial procedures, using detailed AE reporting forms.
- Preliminary signals of efficacy and estimation of parameters required for a future sample size calculation, with outcome data collected at baseline (T0), 3 months (T1) and 6 months (T2).

#### Secondary feasibility outcomes

Secondary feasibility outcomes will include:

- Uptake of the parent coaching sessions, assessed through treatment engagement data, specifically the number of parents or caregivers who accept the offer of the PCS and the number of sessions attended.
- Feasibility of outcome data collection, determined by examining the completeness of the proposed outcome measures (percentage of missing responses and reasons for missing data), to guide measure selection and optimise data collection procedures for the future definitive trial.

#### Key secondary outcome measures

The following secondary outcome measures will be evaluated at baseline, T1 (3 months) and T2 (6 months) using a range of self-report measures collected from both CYP and their parents/guardians: The ‘promise of efficacy’ of the ChUSE intervention and the necessary parameters to inform a sample size calculation for a future efficacy trial.

Measures for CYP

1. Symptom level measured using:

1.1. Manchester Voices Inventory for Children (MAVIC)

1.2. Unusual Experiences Questionnaire (UEQ)

1.3. Brief Self-compassion Scale

2. Health-related quality of life outcomes measured using:

2.1. Stirling Children’s Wellbeing Scale

2.2. EuroQol 5-Dimensional Questionnaire for Youth (EQ-5D-Y)

2.3. KidCOPE scale

2.4. Child Health Utility Instrument (CHU-9D)

2.5. Strengths and Difficulties Questionnaire Self-Report version for 11–17-year-olds

Measures for parents/caregivers

1. Health-related quality of life outcomes measured using:

1.1. Strengths and Difficulties Questionnaire-parent version

1.2. Adult Wellbeing Scale (parents/caregivers)

1.3. Parental Stress Scale, an 18-item questionnaire assessing parents’ feelings about their parenting role, exploring positive (eg, demands on resources, feelings of stress)

1.4. Coping Health Inventory for Parents

1.5. EQ-5D

### Trial design

This study will take the form of a feasibility RCT with an embedded process evaluation. It will employ a single-blind, parallel-group design, comprising two arms: (1) a brief psychological intervention involving 4–6 sessions of individual talking therapy for DSEs, with the optional offer of up to three sessions for parents/caregivers, in addition to TAU and (2) TAU alone. Participants in the intervention arm will also be offered a joint session zero with the trial therapist to discuss taking part in the intervention and to plan appointments. Outcome assessments will be conducted with all participants (n=60 parent–child dyads) at baseline (T0), at the end of the intervention (3-month post-randomisation, T1) and at 6-month post-randomisation (T2). At T2, participants will be invited to take part in qualitative interviews to inform the process evaluation. Participants who do not wish to take part in qualitative interviews will be sent a brief online questionnaire via Qualtrics to enable participants to provide feedback on their experience of being in the trial and their experience of the intervention or CAMHS, depending on which arm of the trial they were randomised to. The trial is due to run between October 2024 and November 2026.

While the primary focus of the study is on feasibility outcomes, the process evaluation will generate both quantitative and qualitative data to enhance understanding of potential mechanisms of change, the acceptability of the intervention, preliminary indications of clinical promise within CAMHS settings as well as likely cost drivers and wider system consequences. Quantitative measures will help to track changes in symptoms and well-being over time, while qualitative feedback will capture participants’ experiences of the intervention, trial procedures and how they have experienced their engagement in the trial. Qualitative interviews with practitioners (n=20) will also be conducted to inform interpretation of contextual factors and future implementation considerations.

### Trial setting

The trial will be conducted across multiple sites, with participant identification, recruitment, and treatment delivery taking place in outpatient CAMHS across three NHS Trusts in Greater Manchester. Should additional recruitment be required, participant identification may be extended to other comparable services within trusts supported by the National Institute for Health and Care Research Research Delivery Network.

### Patient and public involvement and engagement and equality, diversity, inclusion and accessibility

This study has been co-developed with meaningful input from young people, parents and caregivers and professionals with personal lived and clinical experience. A Lived Experience Advisory Group (LEAG), consisting of eight young people, will advise on the study throughout its duration. The LEAG has contributed to the design of study materials, intervention content and recruitment approaches through their involvement with Voice Collective, a lived experience-led community group, to ensure relevance, accessibility and sensitivity to lived experience. Alongside the LEAG, a parent and caregiver advisory group comprising six members provides insights into family needs, language appropriateness and acceptability of procedures from a caregiver’s perspective.

A stakeholder advisory group of 10 professionals, including CAMHS clinicians, education specialists and voluntary sector representatives, will offer strategic guidance on implementation, dissemination and real-world relevance. Governance oversight will be provided by a Trial Steering Committee (TSC), which includes eight members with expertise in child mental health, trial methodology and public involvement.

We are committed to upholding the principles of equality, diversity, inclusion and accessibility across all components of the trial. Recruitment materials and procedures have been co-produced to ensure they are inclusive and appropriate for a range of cultural, linguistic and neurodevelopmental needs. Monitoring of demographic data will help ensure transparency and accountability in terms of equitable access and identify any disparities in participation or outcomes.

### Participants and recruitment

This feasibility trial has been designed to be inclusive and accessible, with eligibility criteria and recruitment procedures shaped by extensive public and patient involvement and engagement (PPIE) and consultation with clinical stakeholders. Recognising that DSEs are not condition-specific, young people will not be excluded based on mental health diagnoses, co-occurring treatments or neurodevelopmental status. The trial and intervention have been specifically designed to be inclusive of neurodiverse participants, and parents/caregivers are invited, but not required, to take part in the parent/caregiver sessions.

#### Eligibility criteria

CYP (1) aged 8–16 years who are (2) currently receiving support from participating CAMHS and who (3) self-identify as experiencing DSEs will be eligible to participate. Given the absence of validated clinical thresholds for DSEs, eligibility will be confirmed using the MAVIC and an adapted version of the Unusual Experiences Questionnaire (UEQ) in screening to determine the presence of at least one self-identified current DSE following initial referral. Although additional funding is not available across all locations, translators and interpreters will be sourced where funding from clinical services is available. If funding for this provision is not available, (4) the young person and their parent/caregiver will need to have conversational English to take part. (5) Current inpatient admission for acute mental health crisis or (6) participation in another active psychological intervention trial will also preclude people from taking part.

#### Participant identification and recruitment

Participants will be identified from existing CAMHS referrals where young people have self-reported DSEs. Research staff will regularly attend multidisciplinary team meetings to support clinicians in identifying eligible participants. Clinicians will provide initial study information to families and seek verbal consent for contact by the research team. The research team will subsequently contact interested families to provide full study details and obtain informed consent from parents/caregivers and young people aged 16 years and over and assent from young people. aged 15 years and under, documented in accordance with local governance procedures.

#### Screening and enrolment

Following consent and assent, screening assessments using the MAVIC and MUSEQ measures will confirm eligibility prior to enrolment. Consenting participants will complete baseline assessments (T0) before randomisation. Young people and parents/caregivers will be offered choices regarding the format and location of assessments (in-person, telephone or video call) and whether to complete these independently or together.

#### Sampling and recruitment targets

As a feasibility trial, a formal power calculation is not required.^49 50^ In line with recommendations for pilot trials, we aim to randomise 60 young people and parent/caregiver dyads.^51 52^ This sample size is considered sufficient to estimate outcome measure variability and inform the design of a future definitive trial. Recruitment will take place over 19 months, with a minimum target of four participants per month in the first year. A recruitment review will be conducted at month 12, with contingency plans to extend to additional NHS trusts if 75% of the target sample has not been achieved 6 months before the end of the trial.

#### Consent, assent and withdrawal

Informed consent and assent will be obtained prior to participation in each stage of the study (T0, T1, T2). Participants may provide consent via signed hard copy, electronic form or audio-recorded verbal consent, depending on preference. The consent process will be clearly documented, and participants will be reminded that participation is voluntary and can be withdrawn at any time without affecting their access to usual clinical care. If a participant withdraws but then wishes to opt back in, they may do so if this is within four weeks of their six-month post-randomisation date for T2 data collection. Additional optional consent provisions will cover audio-recording assessments, use of anonymised data for secondary analysis, future research contact, teaching and sharing study results. Based on media interest in our work in this area previously, there is also an option for participants to opt-in to receive information about future media enquiries or invitations. In line with the Standard Protocol Items: Recommendations for Interventional Trials (SPIRIT statement^53^, predefined criteria for the discontinuation or interruption of the intervention will be applied at the individual participant level, with intervention delivery paused or possibly stopped where a SAE is assessed as being related to the ChUSE intervention procedures.

#### Remuneration and expenses

Participants will receive a £10 shopping voucher as a thank you for completing outcome assessments at T0, T1 and T2. Reasonable travel expenses can be reimbursed on request for in-person research appointments, although the majority of trial procedures will be conducted remotely or during routine clinic visits.

## Measures

### Eligibility screening and baseline assessments

Eligibility will be confirmed via a short screening form (age and presence of at least one self-identified current DSEs using the MAVIC and USQ). Those eligible will proceed to baseline (T0) assessments, including demographic, clinical history and validated outcome measures. Ineligible participants will be debriefed, receive a voucher and be offered a summary of their screening results to share with their clinical team.

Assessments will be flexible, delivered face-to-face or remotely, with options for breaks or multiple shorter sessions. After completing the baseline assessment, the young person (and, by proxy, their parent/caregiver) will be randomised to receive either TAU or ChUSE+TAU. Young people’s preferences around who is present during assessment will be respected. Measures will be repeated at T1 and T2, as shown in table 1 and table 2.

**Table 1:** Assessments for children and young people

T0 (baseline)	T1 (3-month follow-up)	T2 (6-month follow-up)
Screen: MAVIC	MAVIC	MAVIC
Screen: Unusual Sensory Experiences Questionnaire	–	–
Demographics	–	–
Strengths and Difficulties Questionnaire (SDQ)	SDQ	SDQ
Kidcope Scale	Kidcope Scale	Kidcope Scale
The Stirling Children’s Wellbeing Scale	The Stirling Children’s Wellbeing Scale	The Stirling Children’s Wellbeing Scale
Self-Compassion Scale Short Form (SCS-SF)	SCS-SF	SCS-SF
EQ-5D-Y	EQ-5D-Y	EQ-5D-Y
CHU-9D	CHU-9D	CHU-9D
CSS	CSS	CSS
		TAU checklist

CHU-9D, Child Health Utility Instrument; CSS, Child Stigma Scale; EQ-5D-Y, EuroQol 5-Dimensional Questionnaire for Youth; MAVIC, Manchester Voices Inventory for Children; TAU, treatment as usual.

**Table 2:** Parent/carergiver measures

T0 (baseline)	T1 (3-month follow-up)	T2 (6-month follow-up)
Demographics	–	–
SDQ (parent version)	SDQ (parent version)	SDQ (parent version)
Adult Wellbeing Scale	Adult Wellbeing Scale	Adult Wellbeing Scale
Parental Stress Scale	Parental Stress Scale	Parental Stress Scale
Coping Health Inventory for Parents	Coping Health Inventory for Parents	Coping Health Inventory for Parents
EQ-5D	EQ-5D	EQ-5D

EQ-5D, EuroQol-5 Dimension questionnaire; SDQ, Strengths and DifficultiesQuestionnaire.

Participants do not have to complete all questions in the outcome measures. At the end of the baseline assessment, participants will be informed of their trial arm allocation by a member of the research team.

### Randomisation and blinding

Participants will be randomly allocated on a 1:1 basis to either (1) ChUSE talking therapy plus parent sessions alongside TAU or (2) TAU alone. Randomisation will be conducted independently using a web-based service (sealedenvelope.com), with concealed allocation via a pseudo-random sequence employing random permuted blocks of varying sizes, unknown to the study team.

Following baseline assessment, research workers will notify the chief investigator (CI) or a delegate about participant eligibility for randomisation. The CI or trial therapist (or delegate) will then enter the participant ID into the secure online system, which will immediately generate the allocation. Allocation outcomes will be communicated only to unblinded team members (CI, Co-CI and the trial therapist) and centrally logged on the secure platform and a restricted-access randomisation log.

Blinding will be maintained for research workers and trainees responsible for data collection. Research workers and trainees will not access clinical notes or treatment information post-randomisation and will be routinely reminded, along with participants and clinical teams, to avoid disclosing treatment allocations during assessments. The CI, therapist and co-investigators will remain unblinded in line with standard practice for psychological intervention trials. Any breaches of blinding will be documented, and where possible, follow-up will be reassigned to a blinded researcher.

### Interventions

#### Comparator: treatment as usual

Participants allocated to the TAU arm will continue to receive care as usual in CAMHS. The trial team will not intervene in routine care or request clinicians to withhold any referrals or treatments typically offered to participants. This aligns with established practices for pragmatic psychological intervention trials.^54^ In the absence of national clinical guidelines specifically addressing DSEs for children, clinicians will be asked to describe what interventions and supports they provide to these young people and their families. These data will be systematically collected and tabulated using a structured checklist and free-text responses, offering novel insights into routine clinical practices for DSE.^55^

#### ChUSE intervention

The feasibility trial will deliver the ChUSE intervention through qualified, NHS-employed, HCPC-registered clinical psychologists trained and supervised in the protocol by the study’s CI. The manualised intervention will comprise ideally seven 50-min sessions (four child-focused and up to three parent coaching sessions) delivered over a 3-month period. While the intervention will always remain brief, there is some flexibility in the number of sessions offered to young people to support engagement for up to a maximum of six sessions. This is to allow for pacing of content and accommodate breaks or shorter sessions as required by families with different needs. There will also be a session zero offered to all participants in the trial arm to attend as a child–parent/caregiver dyad. After each session, the therapist will complete an adherence checklist to monitor progression towards therapy milestones, the use of intervention strategies in line with the manual and any deviations.^56^

Parents/caregivers of children in the intervention arm will be offered the parent sessions alongside the child-focused therapy. Importantly, parental engagement in the parent sessions will not be a prerequisite for children to access individual therapy. The structure and delivery format of both child and parent components were informed by PPIE consultations, which recommended offering a choice of in-person or online sessions via Microsoft Teams to enhance accessibility.

#### ChUSE child-focused intervention

The child-focused therapy is a psychological intervention targeting DSEs, delivered across 4–6 structured sessions. Content draws on contemporary research on child distress, USEs and psychosis-like phenomena.

- The first session will focus on exploring the young person’s DSEs and goals, psychoeducation and normalisation.^1 30 57^
- The second will introduce formulation and multisensory coping strategies based on compassion-focused therapy principles, shaped by recommendations from young people with lived experience and the growing compassion for voices literature.^17 23 32^
- The third session will include cognitive appraisal work, coping skills practice and simple self-formulation exercises.^31 33^
- The final session will focus on compassion-focused reflection, consolidation of coping strategies and safety/future planning.

#### ChUSE parent training programme

Parents/caregivers will be invited to attend three structured sessions designed to complement the child’s therapy.

- The first session offers psychoeducation, normalisation, reappraisal strategies and therapist-guided, scenario-based problem-solving.^19 57^
- The second introduces compassion-focused coping techniques and self-kind containment strategies.
- The third provides therapist-guided reflective discussions, consolidating new knowledge and coping skills.

Between-session homework will involve reading psychoeducational materials and practising coping strategies, facilitating skills generalisation and enhancing therapeutic outcomes.^58^

#### Fidelity and process monitoring

With participant consent, therapy sessions may be audio- or video-recorded for supervision and fidelity monitoring purposes. Declining to consent for recording will not affect the care received. Recordings will be destroyed following review or fidelity assessment.

Fidelity will be monitored via a therapist-completed intervention integrity checklist developed for the study. Additionally, a random sample of 10% of recorded sessions will be rated using the CBT-SCYP to assess adherence to the ChUSE manual.^59^ The therapeutic alliance will also be measured post-intervention using the Therapeutic Alliance Scale for Children and Caregivers, completed by children and parents/caregivers.^60^

### Follow-up assessments and safeguarding procedures

Participants will be invited to complete follow-up assessments at 3 months (T1) and 6 months (T2) post-randomisation. These assessments will replicate the baseline battery of measures, as shown in tables 1 and 3. To enhance active safety monitoring, AEs will be systematically assessed during each contact point and through consultation with clinical notes.

**Table 3:** Measures and completion times

Instrument used in the proposed trial	Number of items	Indicative completion time
Strengths and Difficulties Questionnaire Self-Report version for 11–17-year-olds^69^	25	10–15 min
Manchester Voices Inventory for Children (MAVIC;^23^)	18	10–12 min
Unusual Experiences Questionnaire (MUSEQ;^70^)	9 (only issued at T0)	10–15 min
Stirling Children’s Wellbeing Scale^71^	12	8–10 min
Brief Self-compassion Scale^72^	12	8–10 min
KidCOPE scale^73^	15	10–20 min
EQ-5D-Y^63^	5	5 min
Child Health Utility Instrument (CHU-9D;^64^)	9	5–8 min
Child Stigma Scale (CSS) – adapted multisensory experiences^74^	7	5 min
Treatment as usual checklist (TAU checklist)	12	8–10 min

EQ-5D, EuroQol-5 Dimension questionnaire; EQ-5D-Y, EuroQol 5-Dimensional Questionnaire for Youth; UEQ, Unusual Experiences Questionnaire.

Each assessment is expected to last approximately 1 hour, with options for breaks or multiple shorter sessions to accommodate participants’ needs (see table 3). T2 may take up to 2 hours, as this data point includes qualitative interviews. For all participants, there is then an option to provide further qualitative feedback through a Qualtrics survey, based on the same questions as the semi-structured interview guides. There is one survey for CYP and one for parents/caregivers. On completion of the final follow-up, participants will be formally debriefed about the feasibility of the RCT and, where consent has been provided, reminded about possible future contact for research updates or follow-up studies.

To minimise missing data, the research team will make multiple attempts to contact participants before considering them lost to follow-up. Data collection methods will include in-person, telephone, video conferencing or postal options. Any spurious or incomplete data will be reviewed by the research team, who will determine appropriate actions such as verification or re-collection.

Safeguarding procedures will adhere to local and national guidelines. The CI will be informed of any safeguarding concerns and referrals will be made to the appropriate safeguarding team for the child’s local area. In cases of immediate risk, the emergency duty team will be contacted directly. Contact details for safeguarding services across Greater Manchester’s 10 boroughs will be held by designated trial staff, and procedures will follow the Greater Manchester Mental Health NHS Foundation Trust safeguarding policies.

### Progression criteria

Progression to a definitive trial will be determined using pre-specified feasibility criteria across five domains (see table 4). Recruitment feasibility will be assessed by the number of parent–child dyads consented and randomised per month, alongside referral sources and reasons for non-eligibility or non-participation. Feasibility will be demonstrated if an average of at least three dyads are recruited per month. Recruitment of two dyads per month will indicate conditional feasibility, requiring the identification of additional recruitment strategies, while recruitment averaging one participant per month (fewer than 20 participants overall) will indicate that progression is not feasible within the current design. However, these criteria will be applied with some flexibility where there are contextual factors to consider, such as the impact of school holidays and national holidays on families’ engagement with services.

**Table 4:** Progression criteria

Domain	Green	Amber	Red
Recruitment	≥3 dyads/month	2 dyads/month	≤1 /month (<20 total)
Intervention completion	≥70% complete	50–70%	<50%
Trial retention	≥70% retained	30–70%	<30%
Parent programme uptake	>70% uptake	50–70%	<50%
Safety (SAEs)	<5% SAE	5–10%	>10% SAE

SAEs, serious adverse events.

Intervention feasibility will be evaluated through adherence and attendance. Progression will be supported if at least 70% of participants allocated to the intervention arm complete the full course of treatment, defined as engagement with all four CYP sessions. Completion rates of 50–70% will indicate conditional feasibility, contingent on identifying barriers and potential adaptations informed by qualitative interviews and adherence data. Completion rates below 50% will indicate that progression to a definitive trial is not feasible without substantial modification of the intervention or delivery model.

Trial retention will be assessed by the proportion of participants completing end-of-treatment and follow-up assessments. Retention of at least 70% of participants will demonstrate feasibility. Retention rates between 30% and 70% will indicate conditional feasibility, requiring the development of strategies to address attrition, informed by reasons for withdrawal, recorded at the point of withdrawal in a participant tracker and qualitative interview data. Retention below 30% will indicate non-feasibility of progression under the current trial design.

The feasibility of including the parent training programme in a future definitive trial will be determined by parental uptake and attendance. Inclusion of the parent sessions will be considered feasible if more than 70% of parents allocated to the intervention arm take up the offer. Uptake of 50–70% will indicate conditional feasibility, dependent on evidence of session attendance, potential added benefit and health economic data suggesting cost-effectiveness. Uptake below 50% will indicate that inclusion of the parent sessions is not feasible within the current design.

Safety and acceptability of trial procedures will be assessed through monitoring of SAEs. Feasibility will be demonstrated if fewer than 5% of participants experience SAEs related to trial procedures. SAE rates between 5% and 10% will indicate conditional feasibility, requiring the identification of additional risk-mitigation strategies informed by qualitative data. SAE rates exceeding 10% will indicate that progression to a definitive trial is not feasible. All SAEs will be reviewed by the TSC Chair and reported to the sponsor as appropriate.

#### Analysis of key feasibility outcomes

Descriptive statistics will be employed to summarise key feasibility and acceptability outcomes, including trial recruitment, retention, therapy engagement and treatment fidelity. Participant flow through the trial will be reported in accordance with the Consolidated Standards of Reporting Trials (CONSORT) extension for feasibility and pilot trials,^49^ detailing the number of referrals received per month, recruitment sources, number of participants contacted and assessed for eligibility, numbers consented (with reasons for ineligibility or declining participation when available), randomisation to trial arms, adherence to allocated interventions and retention at the 3- and 6-month follow-up assessments.

All quantitative data analysis will be conducted according to a pre-specified statistical analysis plan prepared by our trial statistician (co-author LAC) and available on request from the corresponding author. Descriptive analyses of therapy session records and fidelity checklists will estimate the adherence of therapists to the intervention protocol. AEs and SAEs will be tabulated by trial arm and reviewed to assess the safety and acceptability of the intervention and study procedures.

In addition, descriptive statistics (mean, SD, minimum, maximum, range and percentage of missing data) will be reported for all outcome measures at baseline and follow-up assessments, disaggregated by trial arm. These data will inform feasibility parameters for a future definitive trial, including estimates of variance and preliminary outcome differences. No hypothesis testing for clinical effectiveness (p values) will be conducted, but where appropriate, mean differences, SD and 80% CIs will be calculated at follow-up time points to provide a preliminary indication of the ‘promise of efficacy’ of the ChUSE intervention on valued trial outcomes.^61^

To support the feasibility of future health economic evaluation, the completeness and quality of data collected using the EQ-5D,^62^ EQ-5D-Y^63^ and CHU-9D^64^ will be summarised. Intervention and TAU service costs will be estimated using national unit cost data from NHS England and the Personal Social Services Research Unit.

#### Qualitative evaluation

Interviews will be guided by topic guides co-developed with the PPIE groups and refined iteratively throughout the trial. Qualitative inquiry will be undertaken to explore participant and clinician experiences of trial processes and intervention delivery, which is essential for informing the development and future implementation of complex interventions.^65^ Semi-structured interviews will be conducted by members of the research team at mutually convenient locations, either face-to-face, online via Microsoft Teams or by telephone. Young people may be accompanied by a trusted adult if they wish.

Interviews will be audio-recorded, transcribed verbatim using secure transcription software and subsequently reviewed and anonymised by a second member of the research team. Transcripts will be analysed using the Framework Method,^66^ a structured and transparent approach suitable for multidisciplinary health research teams.^67^ Regular interpretation meetings will be held among the qualitative research team and LEAG to promote reflexivity and transparency during the analytic process.

Future access to anonymised final datasets will be considered case-by-case by the CI and TSC, ensuring no risk of participant identification. Study findings will be disseminated through peer-reviewed publications, conference presentations, public forums and participant summaries, in line with NIHR publication requirements.

## Nested MAVIC-R

This nested validation study within the ChUSE Trial aims to assess the reliability, validity and usability of the MAVIC-R. MAVIC-R is a novel communication and assessment tool designed to help young people describe their USEs (which may be comforting, banal or distressing) or DSEs in a non-pathologising, supportive manner. This study seeks to explore the tool’s psychometric properties and explore its practical applications in NHS CAMHS and related services.

### Research questions

1. How effectively does MAVIC-R facilitate communication about DSEs with young people?
2. Which components are most helpful in exploring personal appraisals and meanings of DSEs?
3. How do young people, caregivers and professionals perceive the tool’s utility?
4. What are the practical and therapeutic implications of its use in CAMHS?

### Research design

#### Phase 1: MAVIC-R refinement

Discussions with the ChUSE PPIE groups will inform updates to the existing MAVIC, expanding its scope from voice-hearing to multisensory experiences. Insights from these consultations will guide revisions to language and content.

#### Phase 2: validation study

A mixed-methods design will assess the psychometric properties and practical acceptability of the MAVIC-R:

- **Quantitative**: An online survey with young people will assess item properties and factor structure via exploratory factor analysis (EFA).
- **Qualitative**: Mental health professionals will trial the tool in practice and provide feedback through interviews on its implementation and acceptability.

### Participant recruitment

- Up to 150 young people (aged 8–18) with lived experience of USEs, recruited through professional and lived experience networks, such as voice collective.
- 10 parents/caregivers of young people with USEs.
- 10 multiservice professionals working with young people experiencing USEs.

Inclusion criteria

- Young people aged 8–18, fluent in conversational English, with personal experiences of one or more USEs.
- Caregivers with current or past experience of supporting a young person with USEs.
- Professionals with regular experience working with young people experiencing USEs.

Exclusion criteria

- Young people outside the specified age range or whose current mental health prevents participation.
- Professionals/caregivers without relevant experience.
- Autistic young people whose USEs are solely attributed to sensory processing differences.

### Recruitment process

Participants will be recruited via the RRDN, NHS networks, peer organisations (eg, voice collective) and professional contacts. The same referral process as the wider ChUSE trial will be followed, with practitioners completing brief referral forms with consent.

### Consent/assent

Participants will provide electronic consent via Qualtrics for surveys or audio-recorded consent for in-person interviews. For those under 16, caregiver consent and participant assent will be obtained.

### Data collection and analysis

Audio-recorded interviews and focus groups will be transcribed and analysed thematically^68^ using NVivo. Qualitative survey data will also be analysed thematically, and quantitative survey data will be analysed in SPSS. Data will be screened for missingness and item correlations examined and sampling adequacy assessed via KMO and Bartlett’s tests. EFA using maximum likelihood extraction with oblique rotation will determine factor structure, guided by scree plots, Kaiser’s criterion and parallel analysis. Reliability will be tested via Cronbach’s alpha and item-total correlations.

### Expected outcomes and dissemination

If validated, the MAVIC-R will provide a practical, non-stigmatising tool for facilitating discussions about USEs with young people, supporting tailored, person-centred care. Its use could foster more holistic, youth-informed care pathways within CAMHS and other youth services. Findings will be disseminated via peer-reviewed publications, stakeholder summaries and multimedia resources for young people, families and professionals. This study will also enable the inclusion of a validated, youth-specific measure in a future definitive ChUSE trial.

### Trial endpoints, ethical and regulatory considerations and data management

The intervention period for this trial is scheduled to conclude by 1 March 2026, with all data collection for the trial completed by 1 June 2026. The nested validation study will be completed by 1 December 2026. Prior to study commencement, the protocol, consent materials and study documents will obtain favourable NHS Research Ethics Committee (REC) and Health Research Authority (HRA) approvals, with local capacity and capability agreements from participating NHS trusts. The study will adhere to the Declaration of Helsinki, Good Clinical Practice and the UK Policy Framework for Health and Social Care Research (2017).

Regulatory compliance will be maintained through appropriate approvals for all participating sites, timely submission of amendments and regular oversight from the sponsor and TSC. Protocol deviations will be formally logged and corrective actions agreed on collaboratively.

Risk management procedures will include bespoke standard operating procedure (SOPs) for safeguarding, clear limits of confidentiality, procedures for managing participant distress and strict lone working and infection control policies for face-to-face contact.

AEs and SAEs will be monitored closely, recorded according to established SOPs and reviewed for severity, relatedness, expectedness and seriousness. SAEs that are unexpected and related will be reported to the REC and sponsor in line with standard HRA timelines.

Data management will comply with the Data Protection Act 2018 and GDPR. All personal data will be pseudonymised, securely stored and deleted or anonymised at study end. Research data will be stored on encrypted servers with access restricted to authorised study personnel. Final datasets and supporting materials will be archived securely with the Sponsor.

## Ethics and dissemination

The ChUSE Trial received ethical approval on 2 May 2024 through the Health Research Authority following review by the West Midlands - South Birmingham Research Ethics Committee (REC) specialising in paediatric research (ID 327343). Any amendments to the protocol will be submitted to the REC committee as modifications. This study was registered with the ISRCTN on 14 June 2024 (https://www.isrctn.com/ISRCTN12245618).

Study findings will be widely disseminated through open access publications and presentations at national and international conferences, alongside more accessible multimedia disseminations developed in conjunction with our PPIE group, such as podcasts, animations, infographics and youth-friendly summaries. In parallel, findings will be disseminated through policy briefings and stakeholder events to inform national guidance and service development, with the aim of influencing policy and practice in child and adolescent mental healthcare. If successful, this study has the potential to inform clinical guidelines, contribute to workforce development through training and supervision models and ultimately improve the quality and safety of services offered to young people in NHS mental health settings.

## Patient and public involvement

Public engagement has been central to the development and design of this study, with young people, families and practitioners contributing to intervention refinement, study materials and data collection processes. Their input ensures that the research is sensitive to the needs and preferences of those it aims to support and that dissemination plans are inclusive and accessible. We will endeavour to adopt the multisensory approach of the ChUSE intervention into the development of multisensory and multimedia translational outputs from the study, suitable and engaging for a range of audiences, in collaboration with our advisory groups.

Dissemination activities will be designed to engage a range of audiences, including young people, families, the public and professionals across health, education and voluntary sectors. A co-produced multimedia portfolio, including short animations, infographics, podcasts and youth-friendly summaries, will support accessible communication of findings. These outputs will be developed in collaboration with the LEAG and shared through digital platforms, schools, CAMHS services and third-sector networks.

## Supplementary Material

Reviewer comments

Author's manuscript
