# Supplementary material for: The ChUSE trial - improving access to psychological therapies for children with distressing sensory experiences in NHS CAMHS: a randomised controlled feasibility trial protocol
Source: BMJ Open. 2026 Sep 10;16(9):e119727. doi: 10.1136/bmjopen-2026-119727 (PMC13584016; doi:10.1136/bmjopen-2026-119727)
Supplement: Supplementary file 1 [file bmjopen-2026-119727.draft_revisions.pdf]

BMJ Open is committed to open peer review. As part of this commitment we make the peer review history of every article we publish publicly available.

When an article is published we post the peer reviewers' comments and the authors' responses online. We also post the versions of the paper that were used during peer review. These are the versions that the peer review comments apply to.

The versions of the paper that follow are the versions that were submitted during the peer review process. They are not the versions of record or the final published versions. They should not be cited or distributed as the published version of this manuscript.

BMJ Open is an open access journal and the full, final, typeset and author-corrected version of record of the manuscript is available on our site with no access controls, subscription charges or pay-per-view fees (http://bmjopen.bmj.com).

If you have any questions on BMJ Open's open peer review process please email info.bmjopen@bmj.com

The ChUSE Trial - Improving Access to Psychological Therapies for Children with Distressing Sensory Experiences: A Randomised Controlled Feasibility Trial Protocol

Journal:	*BMJ Open*
Manuscript ID	bmjopen-2026-119727
Article Type:	Protocol
Date Submitted by the Author:	24-Mar-2026

Complete List of Authors:	Parry, Sarah ; Pennine Care NHS Foundation Trust, Young People's Mental Health Research Centre; The University of Manchester, Division of Psychology and Mental Health | School of Health Sciences | Faculty of Biology<br>Carr, Sarah; Greater Manchester Mental Health NHS Foundation Trust, Complex Trauma and Resilience Research Unit<br>Carter, Lesley-Anne; The University of Manchester, Centre for Biostatistics, Division of Population Health, Health Services Research and Primary Care<br>Kirk, Sue; The University of Manchester, School of Health Sciences<br>Petrussa, Cinzia; Greater Manchester Mental Health NHS Foundation Trust, Complex Trauma and Resilience Research Unit<br>Malpass, Fiona; Voice Collective, Mind in Camden<br>Shields, Gemma; University of Manchester, Centre for Health Economics<br>Thompson, Alexander; The University of Manchester, Manchester Centre for Health Economics<br>Morrision, Tony; The University of Manchester, Division of Psychology and Mental Health; Greater Manchester Mental Health NHS Foundation Trust, Psychosis Research Unit<br>Varese, Filippo; The University of Manchester, Division of Psychology and Mental Health; Greater Manchester Mental Health NHS Foundation Trust, Complex Trauma and Resilience Research Unit

Keywords:	MENTAL HEALTH, Child & adolescent psychiatry < PSYCHIATRY, Psychosocial Intervention, Clinical Protocols

The ChUSE Trial - Improving Access to Psychological Therapies for Children with Distressing Sensory Experiences: A Randomised Controlled Feasibility Trial Protocol

Authors:

1. Dr Sarah Parry, University of Manchester and Young People's Mental Health Research Centre, Pennine Care NHS Foundation Trust HQ, 225 Old St, Ashton-under-Lyne OL6 7SR, UK Email: sarah.parry@manchester.ac.uk
2. Dr Sarah Carr, Greater Manchester Mental Health NHS Foundation Trust, Manchester UK
3. Dr Lesley-Anne Carter, University of Manchester, Manchester, UK
4. Professor Sue Kirk, University of Manchester, Manchester, UK
5. Cinzia Petrussa, Greater Manchester Mental Health NHS Foundation Trust, Manchester, UK
6. Fiona Malpass, Voice Collective, Mind in Camden, London, UK
7. Gemma Shields, University of Manchester, Manchester, UK
8. Dr Alexander Thompson, University of Manchester, Manchester, UK
9. Professor Tony Morrison, University of Manchester and Greater Manchester Mental Health NHS Foundation Trust, UK
10. Professor Filippo Varese, University of Manchester and Greater Manchester Mental Health NHS Foundation Trust, Manchester, UK

Corresponding Author: Dr Sarah Parry, University of Manchester and Young People's Mental Health Research Centre, Pennine Care NHS Foundation Trust. University of Manchester | Division of Psychology and Mental Health | School of Health Sciences | Faculty of Biology, Medicine and Health, M13 9PL, UK Email: sarah.parry@manchester.ac.uk

For peer review only

Abstract

Introduction

Distressing sensory experiences (DSEs) are commonly reported by children and young people accessing Child and Adolescent Mental Health Services (CAMHS) in the UK. Despite their prevalence, evidence-based, tailored interventions for this population are scarce. The ChUSE trial aims to address this gap by evaluating the feasibility and acceptability of a novel brief talking therapy for children aged 8-16 years old and an accompanying parent coaching programme.

Methods and Analysis

A single-blind, two-arm randomised controlled feasibility trial will be conducted across three NHS Trusts in Greater Manchester, UK. Sixty young people under the care of CAMHS experiencing DSEs and their parents/caregivers will be randomised 1:1 to receive either Treatment as Usual (TAU) or TAU plus the ChUSE intervention. The intervention involves four child therapy sessions and three optional parent sessions. Primary feasibility outcomes include recruitment and retention rates, treatment adherence, and intervention safety. Secondary objectives assess the feasibility of data collection procedures and gather qualitative insights into trial acceptability and the influence of the intervention upon young people's wellbeing.

Ethics and Dissemination

The ChUSE Trial has received ethical approval through the Health Research Authority following review by a Research Ethics Committee specialising in paediatric research (ID 327343). The feasibility trial addresses a significant clinical gap in CAMHS provision and will also capture information as to current TAU practices, contributing novel data to guide future service provision, as TAU is currently understood to be heterogeneous. The information gathered through this trial will inform the design of a definitive trial evaluating the clinical effectiveness and cost-consequences of an early, age-appropriate intervention for DSEs. Findings and translational outputs will be shared through scientific and public forums to reach a variety of audiences.

Trial registration: ISRCTN12245618

Keywords: distressing sensory experiences, CAMHS, child mental health, cognitive therapy, compassionate mind training, early intervention, feasibility trial

Strengths and limitations of this study

1. Rigorous methodological design with clearly defined procedures that support transparency and replicability.
2. Systematic recruitment and sampling approach to minimise selection bias and enhance the credibility of the findings.
3. Use of validated data-collection tools, increasing reliability and consistency of measurement, paired with more novel and specialised measures to explore any phenomenological change.
4. Potential limitations in generalisability, as the sample is suitably small, so is unlikely to be representative of all relevant populations or contexts.
5. Risk of unmeasured confounding variables, which may influence interpretations despite efforts to control methodological bias.

For peer review only

Word count: 7,429 (not including abstract, references or checklists)

Improving Access to Psychological Therapies for Children with Distressing Sensory Experiences: The ChUSE Trial Protocol

Introduction

Approximately half of children and young people attending Child and Adolescent Mental Health Services (CAMHS) report distressing sensory experiences (DSEs; Jolley, et al. 2018), including auditory and visual hallucinations, feelings of disconnection, and intrusive sensory thoughts (Johns, et al., 2014; Maijer, et al., 2017). Young people can experience DSEs due to the chronic stress of poverty (Hastings et al., 2020), bereavement, abuse and neglect (Corstens & Longden, 2013; Longden et al., 2012), bullying (Domínguez, et al., 2025), medical conditions such as epilepsy (Bisulli, et al., 2018), adverse responses to medication (Capaldi & Carr, 2010), or disrupted sleep (Waters et al., 2018). Distressing voice content has been associated with negative self-schemas and insecure attachments with caregivers (Waters et al., 2018). Young people who experience even transient voice-hearing can report reduced quality of life at later follow-ups compared to their peers (Calkins et al., 2017) and a higher rate of suicidal ideation (Fujita et al., 2015), highlighting the potential long-term impact of voice hearing. Furthermore, DSEs in adolescence have been connected to later experiences of depression and anxiety (Kelleher & Cannon, 2011), indicating a need for intervention as soon as possible to promote coping, wellbeing, and to prevent further difficulties.

A large-scale review (Maijer, et al., 2019) and primary research (e.g., Parry & Varese, 2020; Parry, et al., 2018) indicate that many children begin to report unusual sensory experiences between the ages of 8 and 11 years, with 51% of a community sample describing these experiences as distressing (Parry et al., 2021). From the age of 14, some young people may access specialist support through psychosis care pathways in the UK's National Health Service (NHS). However, referrals to such services can generate additional anxieties, potentially increasing self-stigma and adversely affecting wellbeing (Yang et al., 2010). Current referral pathways for psychosis are not appropriate for many children experiencing DSEs, as only 15–22% of young people identified as at-risk for developing psychosis will access these pathways within a year (Fusar-Poli et al., 2017). This misalignment in referral pathways can increase anxiety for families and delay access to proportionate support in age-appropriate child and adolescent settings.

Providing earlier, non-psychosis-specific support within CAMHS could improve timely access to appropriate, proportionate interventions, while reducing future reliance on specialist services. Early intervention is crucial to promote coping strategies (Hayward, et al., 2025), enhance wellbeing (Kelleher & Cannon, 2011), and to potentially mitigate the development of more severe conditions such as psychosis or mood disorders throughout the turbulent teenage years and into early adulthood.

Auditory verbal hallucinations are thought to be the most common multisensory experience, although this assumption is largely based on data with adults and young people aged 16 years and over. Voices can be comforting or distressing, and young people can experience both comforting and distressing voices at the same time (Parry & Varese, 2021), each sometimes taking on distinct identities (Longden, 2017; Maijer et al., 2019; Alderson-Day et al., 2021). Visual and tactile hallucinations can occur separately or concurrently and have been linked to trauma and/or anxiety

For peer review only

(Jardri et al., 2014; Maijer et al., 2018). The impact of these multisensory experiences varies, influenced by individual psychosocial factors, personal resources, and social support (Løberg et al., 2019; Maijer et al., 2020). Despite nearly 50% of children in CAMHS reporting multisensory experiences (Jolley et al., 2018), there is currently no evidence-based talking therapy available for children with DSEs.

Young people have described benefitting from re-appraisal, normalizing (Maddox, at al., 2013), psychoeducation, externalisation (Jolley et al 2018), multisensory and cognitive restructuring coping strategies (Parry & Varese, 2020; Parry, Loren & Varese, 2021), similar to many of the approaches within cognitive therapies and compassionate mind training (CMT). Emerging findings suggest Compassion for Voices is a beneficial approach for young adult voice hearers, (Norman, Correia, & Paulik, 2020; Heriot-Maitland, McCarthy-Jones, Longden, & Gilbert, 2019; Nisar, et al., 2020). Our recent reviews have explored what is particularly important for young people with DSEs and first episode psychosis in therapeutic relationships (Brown & Parry, 2024), specific gender needs for girls when help-seeking (Roberts & Parry, 2023), family members experiences of help-seeking for DSEs (Rodell & Parry, 2024), and compassionate approaches to coping with voice hearing for young people (Leach, Kelly, & Parry, 2024). These reviews recommend enhancing early support and communication about DSEs for young people, highlighting that girls and young women may experience more dismissive responses to their difficulties when they present to services, and the importance of collaborative therapeutic relationships and compassion-informed approaches for coping with hearing voices.

Drawing from the adult literature, a recent feasibility study on e-CBT for adults with DSEs found 4-6 sessions were acceptable for service users and progression criteria were met for recruitment, retention, and adherence to treatment (Dodgson, et al., 2021). Whilst it is positive to see the development of new interventions amongst the adult population (e.g., Dudley, et al., 2022), there remains a need for DSE research to be undertaken with young people in the unique setting of CAMHS, employing age-appropriate interventions (Maijer, et al., 2019). Suitably tailored youth-focused interventions are limited in their availability and scientific evidence-base due to a scarcity of research with young people and their families to inform tailored protocols (Immonen, Jääskeläinen, Korpela, & Miettunen, 2017). Overall, research has overlooked younger children with DSEs (Stentebjerg-Olesen, et al., 2017), which means important developmental and contextual information is currently missing from our understanding of how children experience DSEs and what support they and their families want from services.

Thus far, we know further work is needed with younger children to improve treatment pathways and reduce delays in accessing suitable support, and that clinicians desire clearer clinical guidance for tailored care (Maijer, et al., 2019). Priorities for tailored care seem to include careful, developmentally informed assessment and caution around the use of structured interviews, which may insufficiently capture the form, function and content of DSEs (Di Luzio et al., 2022). Detailed clinical assessment is also important to distinguish between DSEs and hallucinations caused by neurological conditions such as migraine and occipital epilepsy (D'Agnano et al., 2023). Due to the role traumatic events (Di Luzio et al., 2022; Tang et al., 2025), substance use, sleep disorders, depression and anxiety may play in the experience of DSEs (Tang et al., 2025), a trauma-informed assessment may also be helpful where appropriate support can then be offered. Additionally, DSEs have been associated with sensory processing difficulties in paediatric samples for many years, although causal relationships and treatment implications remain unclear (Nishiura et al., 2024). The ChUSE Trial is open to children in core CAMHS, autistic children, and children currently being assessed for neurodiversity in CAMHS. Adopting a neuroinclusive approach as early as possible in the

For peer review only

treatment development process helps to ensure the final intervention is more acceptable and useful for all.

Developmental processes, including executive functioning, social cognition and language development, are thought to influence DSE phenomenology (Fernyhough et al., 2025), although further research with younger children is needed to understand how to inform psychological support for DSEs. Overall, further research is needed to design developmentally sensitive assessments, interventions, treatment strategies, and to improve psychosocial outcomes (Kelleher, 2025). Drawing on this emerging literature base and alongside lived experience consultations between 2016 and 2025, the ChUSE Trial aims to address recommendations for care in CAMHS by offering young people and their parents/caregivers an intervention that mobilises existent knowledge to offer a tailored brief coping focused therapy, whilst assessing feasibility parameters to prepare for a definitive trial.

When supporting younger children with DSEs, it is essential to consider the role and involvement of parents and caregivers. Parental exclusion within services, and feelings of shame and guilt, can contribute towards relational instability within families (Romelli & Pozzi, 2018). Family interventions for psychosis (FIfP) have been found to improve cognitive functioning, reduce symptoms of psychosis, and reduce the likelihood of relapse (Claxton, Onwumere, & Fornells-Ambrojo, 2017). Importantly, carer burden and wellbeing can also improve following a FIfP approach, indicating benefits for the wider family system of parental support, which may be especially relevant for younger children. The parent coaching sessions within the ChUSE Trial will offer a new and tailored approach for parents, offering separate spaces with a tailored therapeutic focus for parent and child.

In a recent review of parent's experiences of seeking help for young people with DSEs, we found many parents cannot find suitable care or are deterred from help-seeking following finding 'nothing on the other side of the door' of children's mental health services for DSEs, which can delay help-finding until the young person is in crisis (Rodell & Parry, 2024). Our primary research with parents and caregivers prior to the ChUSE Trial demonstrated parents want reliable information, skills and strategies to help their child cope (Parry & Varese, 2021). Prior research identified that many families have dissatisfying experiences of help-seeking for hearing voices and would like more opportunities for normalising and holistic care (Kapur, et al., 2014). Therefore, the ChUSE Trial meets an identified need in CAMHS, for children, practitioners and parents alike, offering a symptom-focused, low-intensity, systemic early-intervention, adding significantly to the clinical support for children and their parents and caregivers.

The ChUSE Trial

A significant challenge in treating DSEs for young people is the absence of child-centred research informing clinical guidelines, despite the recognised prevalence of these difficulties within CAMHS (Jolley et al., 2018). CAMHS practitioners frequently report missing the clinical guidance necessary to support this group of young people, resulting in restricted treatment options and delays in care for young people presenting with DSEs. To address this, the ChUSE trial will implement and evaluate an intervention protocol designed to offer early intervention for use within CAMHS. In time, this could provide practitioners with clear, practical guidance and ensure timely, appropriate support for young people.

A challenge for the ChUSE Trial is our limited understanding of what constitutes 'treatment as usual' (TAU) for this population. This adds complexity to the design and delivery of high-quality research in this area. To mitigate this, the ChUSE trial will incorporate a TAU arm, systematically gathering data

For peer review only

on existing care practices across three NHS Trusts. This will provide essential information to inform the design of a future definitive trial.

For peer review only

Methods and Analysis

Aims and Objectives

Aim

To evaluate the feasibility and acceptability of conducting a future, larger-scale, definitive trial to assess the clinical effectiveness and cost-consequences of a brief psychological intervention for young people aged 8–16 years experiencing DSEs, accompanied by up to three parent coaching sessions.

Objectives

The primary objective is to assess key feasibility outcomes and gather data to inform the design of a future multi-site randomised controlled trial (RCT). Specifically, the study will examine:

- The feasibility of recruiting children and their parents or carers to the trial.
- The retention of participants throughout the trial period.
- Engagement with and adherence to the intervention and trial procedures.
- The safety of the intervention and trial procedures.
- Estimation of key parameters to support a sample size calculation for a future definitive efficacy trial.

Secondary Objectives

In addition to the primary feasibility outcomes, this study will address the following feasibility uncertainties:

- To determine the uptake of the optional parent coaching sessions.
- To evaluate the feasibility of collecting a set of proposed outcome measures, informing the selection of appropriate measures and data collection procedures for a future trial.
- To gather qualitative data exploring the acceptability of the intervention and trial procedures from the perspectives of young people, parents and carers, as well as their recommendations for refining the trial design.
- To collect qualitative insights from Child and Adolescent Mental Health Services (CAMHS) practitioners and clinical leads regarding barriers, facilitators, costs and consequences of implementing the intervention within routine CAMHS provision.

Primary Feasibility Outcomes

The main feasibility outcomes for this study are as follows:

- Recruitment rates, measured as the number of family units recruited and randomised per month.
- Participant retention rates, expressed as the proportion of participants completing outcome assessments at each time point.
- Treatment engagement and adherence, assessed by the number of sessions attended by young people and parents or carers allocated to both the ChUSE intervention plus treatment as usual (TAU) arm, and the TAU-only arm.

For peer review only

- Treatment fidelity, determined by the extent to which trial therapists deliver the ChUSE intervention according to the intervention protocol, as measured by fidelity checklists.
- Safety of the intervention, evaluated through the monitoring and recording of adverse events (AEs) and serious adverse events (SAEs), both related and unrelated to the intervention or trial procedures, using detailed AE reporting forms.
- Preliminary signals of efficacy and estimation of parameters required for a future sample size calculation, with outcome data collected at baseline (T0), three months (T1), and six months (T2).

Secondary Feasibility Outcomes

Secondary feasibility outcomes will include:

- Uptake of the parent coaching sessions, assessed through treatment engagement data, specifically the number of parents or carers who accept the offer of the PCS and the number of sessions attended.
- Feasibility of outcome data collection, determined by examining the completeness of the proposed outcome measures (percentage of missing responses and reasons for missing data), to guide measure selection and optimise data collection procedures for the future definitive trial.

Trial Design

This study will take the form of a feasibility randomised controlled trial (RCT) with an embedded process evaluation. It will employ a single-blind, parallel-group design, comprising two arms: (1) a brief psychological intervention involving four-six sessions of individual talking therapy for DSEs, with the optional offer of up to three sessions for parents/caregivers, in addition to treatment as usual (TAU); and (2) TAU alone. Participants in the intervention arm will also be offered a joint session zero with the trial therapist to discuss taking part in the intervention and to plan appointments. Outcome assessments will be conducted with all participants (N=60 parent-child dyads) at baseline (T0), at the end of the intervention (three months post-randomisation, T1), and at six months post-randomisation (T2). At T2, participants will be invited to take part in qualitative interviews to inform the process evaluation. Participants who do not wish to take part in qualitative interviews will be sent a brief online questionnaire via Qualtrics to enable participants to provide feedback on their experience of being in the trial and their experience of the intervention or CAMHS, depending on which arm of the trial they were randomised to.

While the primary focus of the study is on feasibility outcomes, the process evaluation will generate both quantitative and qualitative data to enhance understanding of potential mechanisms of change, the acceptability of the intervention, preliminary indications of clinical promise within CAMHS settings as well as likely cost drivers and wider system consequences. Quantitative measures will help to track changes in symptoms and wellbeing over time, while qualitative feedback will capture participants' experiences of the intervention, trial procedures, and how they have experienced their engagement in the trial. Qualitative interviews with practitioner (N=20) will also

For peer review only

be conducted to inform interpretation of contextual factors and future implementation considerations.

Trial Setting

The trial will be conducted across multiple sites, with participant identification, recruitment, and treatment delivery taking place in outpatient CAMHS across three NHS Trusts in Greater Manchester. Should additional recruitment be required, participant identification may be extended to other comparable services within Trusts supported by the NIHR Research Delivery Network.

Patient and Public Involvement and Engagement (PPIE) and Equality, Diversity, Inclusion and Accessibility (EDIA)

This study has been co-developed with meaningful input from young people, parents and caregivers, and professionals with personal lived and clinical experience. A Lived Experience Advisory Group (LEAG), consisting of eight young people, will advise on the study throughout its duration. The LEAG has contributed to the design of study materials, intervention content and recruitment approaches through their involvement with Voice Collective, a lived experience led community group, to ensure relevance, accessibility, and sensitivity to lived experience. Alongside the LEAG, a Parent and Caregiver Advisory Group comprising six members provides insights into family needs, language appropriateness, and acceptability of procedures from a caregiver's perspective.

A Stakeholder Advisory Group of ten professionals, including CAMHS clinicians, education specialists, and voluntary sector representatives, will offer strategic guidance on implementation, dissemination, and real-world relevance. Governance oversight will be provided by a Trial Steering Committee (TSC), which includes eight members with expertise in child mental health, trial methodology, and public involvement.

We are committed to upholding the principles of equality, diversity, inclusion, and accessibility (EDIA) across all components of the trial. Recruitment materials and procedures have been co-produced to ensure they are inclusive and appropriate for a range of cultural, linguistic, and neurodevelopmental needs. Monitoring of demographic data will help ensure transparency and accountability in terms of equitable access and identify any disparities in participation or outcomes.

Participants and Recruitment

This feasibility trial has been designed to be inclusive and accessible, with eligibility criteria and recruitment procedures shaped by extensive public and patient involvement and engagement (PPIE) and consultation with clinical stakeholders. Recognising that DSEs are not condition-specific, young people will not be excluded based on mental health diagnoses, co-occurring treatments, or neurodevelopmental status. The trial and intervention have been specifically designed to be inclusive of neurodiverse participants, and parents/caregivers are invited, but not required, to take part in the parent/caregiver sessions.

Eligibility Criteria

Children and young people aged 8–16 years who are currently receiving support from participating CAMHS and who self-identify as experiencing DSEs will be eligible to participate. Given the absence of validated clinical thresholds for DSEs, eligibility will be confirmed using the Manchester Voices Inventory for Children (MAVIC) and the unusual sensory experiences questionnaire (MUSEQ) in screening to determine the presence of at least one self-identified current DSE, following initial

For peer review only

referral. Although additional funding is not available across all locations, translators and interpreters will be sourced where funding from clinical services is available. If funding for this provision is not available, the young person and their parent/caregiver will need to have conversational English to take part. Current inpatient admission for acute mental health crisis, or participation in another active psychological intervention trial, will also preclude people from taking part.

Participant Identification and Recruitment

Participants will be identified from existing CAMHS referrals where young people have self-reported DSEs. Research staff will regularly attend multidisciplinary team meetings to support clinicians in identifying eligible participants. Clinicians will provide initial study information to families and seek verbal consent for contact by the research team. The research team will subsequently contact interested families to provide full study details and obtain informed consent from parents/caregivers and young people aged 16 years and over, and assent from young people aged 15 years and under, documented in accordance with local governance procedures.

Screening and Enrolment

Following consent and assent, screening assessments using the MAVIC and MUSEQ measures will confirm eligibility prior to enrolment. Consenting participants will complete baseline assessments (T0) before randomisation. Young people and parents/caregivers will be offered choices regarding the format and location of assessments (in-person, telephone, or video call) and whether to complete these independently or together.

Sampling and Recruitment Targets

As a feasibility trial, a formal power calculation is not required (Eldridge, et al., 2016; Lancaster, et al., 2004). In line with recommendations for pilot trials, we aim to randomise 60 young people and parent/caregiver dyads (Teare, et al., 2014; Whitehead, et al., 2016). This sample size is considered sufficient to estimate outcome measure variability and inform the design of a future definitive trial. Recruitment will take place over 19 months, with a minimum target of four participants per month in the first year. A recruitment review will be conducted at month 12, with contingency plans to extend to additional NHS Trusts if 75% of the target sample has not been achieved 6 months before the end of the trial.

Consent, Assent, and Withdrawal

Informed consent and assent will be obtained prior to participation in each stage of the study (T0, T1, T2). Participants may provide consent via signed hard copy, electronic form, or audio-recorded verbal consent, depending on preference. The consent process will be clearly documented, and participants will be reminded that participation is voluntary and can be withdrawn at any time without affecting their access to usual clinical care. If a participant withdraws but then wishes to opt back in, they may do so, if this is within four weeks of their six-month post-randomisation date for T2 data collection. Additional optional consent provisions will cover audio-recording assessments, use of anonymised data for secondary analysis, future research contact, teaching, and sharing study results. Based on media interest in our work in this area previously, there is also an option for participants to opt-in to receive information about future media enquiries or invitations. In line with the SPIRIT statement, predefined criteria for the discontinuation or interruption of the intervention will be applied at the individual participant level, with intervention delivery paused or possibly stopped where a serious adverse event (SAE) is assessed as being related to the ChUSE intervention procedures.

For peer review only

Remuneration and Expenses

Participants will receive a £15 shopping voucher as a thank you for completing outcome assessments at T0, T1 and T2. Reasonable travel expenses can be reimbursed upon request for in-person research appointments, although the majority of trial procedures will be conducted remotely or during routine clinic visits.

Measures

Eligibility Screening and Baseline Assessments

Eligibility will be confirmed via a short screening form (age and presence of at least one self-identified current distressing sensory experience using the MAVIC and MUSEQ). Those eligible will proceed to baseline (T0) assessments, including demographic, clinical history, and validated outcome measures. Ineligible participants will be debriefed, receive a voucher, and be offered a summary of their screening results to share with their clinical team.

Assessments will be flexible, delivered face-to-face or remotely, with options for breaks or multiple shorter sessions. After completing the baseline assessment, the young person (and, by proxy, their parent/caregiver) will be randomised to receive either TAU or ChUSE+TAU. Young people's preferences around who is present during assessment will be respected. Measures will be repeated at T1 and T2, as shown in table one and three.

Table 1: Assessments for children and young people

T0 (baseline)	T1 (3-month follow-up)	T2 (6-month follow-up)
Screen: MAVIC	MAVIC	MAVIC
Screen: Unusual Sensory Experiences Questionnaire (MUSEQ)	-	-
Demographics	-	-
Strengths and Difficulties Questionnaire (SDQ)	SDQ	SDQ
Kidcope Scale	Kidcope Scale	Kidcope Scale
The Stirling Children's Wellbeing Scale	The Stirling Children's Wellbeing Scale	The Stirling Children's Wellbeing Scale
Self-Compassion Scale Short Form (SCS-SF)	SCS-SF	SCS-SF
EQ-5D-Y	EQ-5D-Y	EQ-5D-Y
CHU-9D	CHU-9D	CHU-9D
CSS	CSS	CSS
-	-	TAU checklist

Table 2: Measures and Completion Times

Instrument used in the proposed trial	Number of items	Indicative completion time
Strengths and Difficulties Questionnaire Self-Report version for 11–17-year-olds (Goodman et al., 1998)	25	10-15 minutes

Manchester Voices Inventory for Children (MAVIC; Parry & Varese, 2021)	18	10-12 minutes
Unusual Experiences Questionnaire (MUSEQ) (Mitchell, et al., 2017)	9 (only issued at T0)	10-15 minutes
Stirling Children's Wellbeing Scale (Liddle, & Carter, 2015)	12	8-10 minutes
Brief Self-compassion Scale (Neff, 2003).	12	8-10 minutes
KidCOPE scale (Spirito et al., 1988)	15	10-20 minutes
EQ-5D-Y (Wille., 2010)	5	5 minutes
Child Health Utility Instrument (CHU-9D) (Stevens, 2012)	9	5-8 minutes
Child Stigma Scale (CSS) – adapted multisensory experiences	7	5 minutes
Treatment as usual checklist (TAU checklist)	12	8-10 minutes

Table 3: Parent/carergiver measures

T0 (baseline)	T1 (3-month follow-up)	T2 (6-month follow-up)
Demographics	-	-
SDQ (parent version)	SDQ (parent version)	SDQ (parent version)
Adult Wellbeing Scale	Adult Wellbeing Scale	Adult Wellbeing Scale
Parental Stress Scale	Parental Stress Scale	Parental Stress Scale
Coping Health Inventory for Parents	Coping Health Inventory for Parents	Coping Health Inventory for Parents
EQ-5D	EQ-5D	EQ-5D

Participants do not have to complete all questions in the outcome measures. At the end of baseline assessment, participants will be informed of their trial arm allocation by a member of the research team.

Randomisation and Blinding

Participants will be randomly allocated on a 1:1 basis to either (1) ChUSE talking therapy plus parent sessions alongside TAU, or (2) TAU alone. Randomisation will be conducted independently using a web-based service (sealedenvelope.com), with concealed allocation via a pseudo-random sequence employing random permuted blocks of varying sizes, unknown to the study team.

For peer review only

Following baseline assessment, research workers will notify the Chief Investigator (CI) or a delegate about participant eligibility for randomisation. The CI or trial therapist (or delegate) will then enter the participant ID into the secure online system, which will immediately generate the allocation. Allocation outcomes will be communicated only to unblinded team members (CI, Co-CI, and the trial therapist) and centrally logged on the secure platform and a restricted-access randomisation log.

Blinding will be maintained for research workers and trainees responsible for data collection. Research workers and trainees will not access clinical notes or treatment information post-randomisation and will be routinely reminded, along with participants and clinical teams, to avoid disclosing treatment allocations during assessments. The CI, therapist, and co-investigators will remain unblinded in line with standard practice for psychological intervention trials. Any breaches of blinding will be documented, and where possible, follow-up will be reassigned to a blinded researcher.

Interventions

Comparator: Treatment as Usual (TAU)

Participants allocated to the TAU arm will continue to receive care as usual in CAMHS. The trial team will not intervene in routine care or request clinicians to withhold any referrals or treatments typically offered to participants. This aligns with established practices for pragmatic psychological intervention trials (Craig et al., 2008). In the absence of national clinical guidelines specifically addressing DSEs for children, clinicians will be asked to describe what interventions and supports they provide to these young people and their families. These data will be systematically collected and tabulated using a structured checklist and free-text responses, offering novel insights into routine clinical practices for DSE (National Institute for Health and Care Excellence, 2023).

ChUSE Intervention

The feasibility trial will deliver the ChUSE intervention through a qualified, NHS-employed, HCPC-registered Clinical Psychologists, trained and supervised in the protocol by the study's Chief Investigator (CI). The manualised intervention will comprise of ideally seven 50-minute sessions (four child-focused and up to three parent coaching sessions) delivered over a three-month period. While the intervention will always remain brief, there is some flexibility in the number of sessions offered to young people to support engagement for up to a maximum of six sessions. This is to allow for pacing of content and accommodate breaks or shorter sessions as required by families with different needs. There will also be a session zero offered to all participants in the trial arm to attend as a child-parent/caregiver dyad. After each session, the therapist will complete an adherence checklist to monitor progression towards therapy milestones, the use of intervention strategies in line with the manual, and any deviations (Waltz et al., 1993).

Parents/caregivers of children in the intervention arm will be offered the parent sessions alongside the child-focused therapy. Importantly, parental engagement in the parent sessions will not be a prerequisite for children to access individual therapy. The structure and delivery format of both child and parent components were informed by PPIE consultations, which recommended offering a choice of in-person or online sessions via Microsoft Teams to enhance accessibility.

ChUSE Child-Focused Intervention

For peer review only

The child-focused therapy is a psychological intervention targeting DSEs, delivered across four-six structured sessions. Content draws upon contemporary research on child distress, unusual sensory experiences, and psychosis-like phenomena.

- The first session will focus on exploring the young person's DSEs and goals, psychoeducation and normalisation (Jolley et al., 2017; Jolley et al., 2018; Maddox, 2013).
- The second will introduce formulation and multisensory coping strategies based on compassion-focused therapy (CFT) principles, shaped by recommendations from young people with lived experience and the growing compassion for voices literature (Parry & Varese, 2020; Parry et al., 2021; Heriot-Maitland et al., 2019).
- The third session will include cognitive appraisal work, coping skills practice, and simple self-formulation exercises (Nisar et al., 2020; Norman et al., 2020).
- The final session will focus on compassion-focused reflection, consolidation of coping strategies, and safety/future planning.

ChUSE Parent Training Programme

Parents/caregivers will be invited to attend three structured sessions designed to complement the child's therapy.

- The first session offers psychoeducation, normalisation, reappraisal strategies, and therapist-guided, scenario-based problem-solving (Jolley et al., 2017; Parry et al., 2021).
- The second introduces compassion-focused coping techniques and self-kind containment strategies.
- The third provides therapist-guided reflective discussions, consolidating new knowledge and coping skills.

Between-session homework will involve reading psychoeducational materials and practising coping strategies, facilitating skills generalisation and enhancing therapeutic outcomes (Kazantzis, et al., 2016).

Fidelity and Process Monitoring

With participant consent, therapy sessions may be audio- or video-recorded for supervision and fidelity monitoring purposes. Declining to consent for recording will not affect the care received. Recordings will be destroyed following review or fidelity assessment.

Fidelity will be monitored via a therapist-completed intervention integrity checklist developed for the study. Additionally, a random sample of 10% of recorded sessions will be rated using the Cognitive Behaviour Therapy Scale for Children and Young People (CBT-SCYP) to assess adherence to the ChUSE manual (Stallard, et al., 2014). The therapeutic alliance will also be measured post-intervention using the Therapeutic Alliance Scale for Children and Caregivers (TASC), completed by children, and parents/caregivers (Shirk & Saiz, 1992).

Follow-Up Assessments and Safeguarding Procedures

Participants will be invited to complete follow-up assessments at three months (T1) and six months (T2) post-randomisation. These assessments will replicate the baseline battery of measures, as

For peer review only

shown in Tables One and Three. To enhance active safety monitoring, adverse events (AEs) will be systematically assessed during each contact point and through consultation with clinical notes.

Each assessment is expected to last approximately one hour, with options for breaks or multiple shorter sessions to accommodate participant's needs. T2 may take up to two hours, as this data point includes qualitative interviews. For all participants, there is then an option to provide further qualitative feedback through a Qualtrics survey, based on the same questions as the semi-structured interview guides. There is one survey for children and young people, and one for parents/caregivers. Upon completion of the final follow-up, participants will be formally debriefed about the feasibility RCT and, where consent has been provided, reminded about possible future contact for research updates or follow-up studies.

To minimise missing data, the research team will make multiple attempts to contact participants before considering them lost to follow-up. Data collection methods will include in-person, telephone, video conferencing, or postal options. Any spurious or incomplete data will be reviewed by the research team, who will determine appropriate actions such as verification or re-collection.

Safeguarding procedures will adhere to local and national guidelines. The Chief Investigator (CI) will be informed of any safeguarding concerns, and referrals will be made to the appropriate safeguarding team for the child's local area. In cases of immediate risk, the Emergency Duty Team will be contacted directly. Contact details for safeguarding services across Greater Manchester's ten boroughs will be held by designated trial staff, and procedures will follow the Greater Manchester Mental Health NHS Foundation Trust safeguarding policies.

Progression Criteria

Progression to a definitive trial will be determined using pre-specified feasibility criteria across five domains. Recruitment feasibility will be assessed by the number of parent–child dyads consented and randomised per month, alongside referral sources and reasons for non-eligibility or non-participation. Feasibility will be demonstrated if an average of at least three dyads are recruited per month. Recruitment of two dyads per month will indicate conditional feasibility, requiring the identification of additional recruitment strategies, while recruitment averaging one participant per month (fewer than 20 participants overall) will indicate that progression is not feasible within the current design. However, these criteria will be applied with some flexibility where there are contextual factors to consider, such as the impact of school holidays and national holidays on family's engagement with services.

Intervention feasibility will be evaluated through adherence and attendance. Progression will be supported if at least 70% of participants allocated to the intervention arm complete the full course of treatment, defined as engagement with all four CYP sessions. Completion rates of 50–70% will indicate conditional feasibility, contingent on identifying barriers and potential adaptations informed by qualitative interviews and adherence data. Completion rates below 50% will indicate that progression to a definitive trial is not feasible without substantial modification of the intervention or delivery model.

Trial retention will be assessed by the proportion of participants completing end-of-treatment and follow-up assessments. Retention of at least 70% of participants will demonstrate feasibility. Retention rates between 30% and 70% will indicate conditional feasibility, requiring the development of strategies to address attrition, informed by reasons for withdrawal, recorded at the point of withdrawal in a participant tracker, and qualitative interview data. Retention below 30% will indicate non-feasibility of progression under the current trial design.

For peer review only

The feasibility of including the parent training programme in a future definitive trial will be determined by parental uptake and attendance. Inclusion of the parent sessions will be considered feasible if more than 70% of parents allocated to the intervention arm take up the offer. Uptake of 50–70% will indicate conditional feasibility, dependent on evidence of session attendance, potential added benefit, and health economic data suggesting cost-effectiveness. Uptake below 50% will indicate that inclusion of the parent sessions is not feasible within the current design.

Safety and acceptability of trial procedures will be assessed through monitoring of serious adverse events (SAEs). Feasibility will be demonstrated if fewer than 5% of participants experience SAEs related to trial procedures. SAE rates between 5% and 10% will indicate conditional feasibility, requiring the identification of additional risk-mitigation strategies informed by qualitative data. SAE rates exceeding 10% will indicate that progression to a definitive trial is not feasible. All SAEs will be reviewed by the Trial Steering Committee Chair and reported to the sponsor as appropriate.

Analysis of Key Feasibility Outcomes

Descriptive statistics will be employed to summarise key feasibility and acceptability outcomes, including trial recruitment, retention, therapy engagement, and treatment fidelity. Participant flow through the trial will be reported in accordance with the CONSORT extension for feasibility and pilot trials (Eldridge et al., 2016), detailing the number of referrals received per month, recruitment sources, number of participants contacted and assessed for eligibility, numbers consented (with reasons for ineligibility or declining participation when available), randomisation to trial arms, adherence to allocated interventions, and retention at the three- and six-month follow-up assessments.

All quantitative data analysis will be conducted according to a pre-specified statistical analysis plan prepared by our trial statistician (co-author LAC) and available upon request from the corresponding author. Descriptive analyses of therapy session records and fidelity checklists will estimate the adherence of therapists to the intervention protocol. Adverse events (AEs) and serious adverse events (SAEs) will be tabulated by trial arm and reviewed to assess the safety and acceptability of the intervention and study procedures.

In addition, descriptive statistics (mean, standard deviation, minimum, maximum, range, and percentage of missing data) will be reported for all outcome measures at baseline and follow-up assessments, disaggregated by trial arm. These data will inform feasibility parameters for a future definitive trial, including estimates of variance and preliminary outcome differences. No hypothesis testing for clinical effectiveness (p-values) will be conducted, but where appropriate, mean differences, standard deviations, and 80% confidence intervals will be calculated at follow-up time points to provide a preliminary indication of the 'promise of efficacy' of the ChUSE intervention on valued trial outcomes (Lee, 2014).

To support the feasibility of future health economic evaluation, the completeness and quality of data collected using the EQ-5D (Devlin & Brooks, 2017), EQ-5D-Y (Wille et al., 2010), and CHU-9D (Stevens, 2012) will be summarised. Intervention and TAU service costs will be estimated using national unit cost data from NHS England and the Personal Social Services Research Unit.

Qualitative Evaluation

For peer review only

Interviews will be guided by topic guides co-developed with the PPIE groups and refined iteratively throughout the trial. Qualitative inquiry will be undertaken to explore participant and clinician experiences of trial processes and intervention delivery, which is essential for informing the development and future implementation of complex interventions (Martin-Kerry et al., 2019). Semi-structured interviews will be conducted by members of the research team at mutually convenient locations, either face-to-face, online via Microsoft Teams, or by telephone. Young people may be accompanied by a trusted adult if they wish.

Interviews will be audio-recorded, transcribed verbatim using secure transcription software, and subsequently reviewed and anonymised by a second member of the research team. Transcripts will be analysed using the Framework Method (Ritchie & Lewis, 2003), a structured and transparent approach suitable for multidisciplinary health research teams (Gale et al., 2013). Regular interpretation meetings will be held among the qualitative research team and LEAG to promote reflexivity and transparency during the analytic process.

Future access to anonymised final datasets will be considered case-by-case by the CI and Trial Sterring Committee, ensuring no risk of participant identification. Study findings will be disseminated through peer-reviewed publications, conference presentations, public forums, and participant summaries, in line with NIHR publication requirements.

Nested Validation Study: Manchester Voices Inventory for Children and Young People – Revised (MAVIC-R)

This nested validation study within the ChUSE Trial aims to assess the reliability, validity, and usability of the Manchester Voices Inventory for Children and Young People - Revised (MAVIC-R). MAVIC-R is a novel communication and assessment tool designed to help young people describe their unusual sensory experiences (USEs, which may be comforting, banal, or distressing) or DSEs in a non-pathologising, supportive manner. This study seeks to explore the tool's psychometric properties and explore its practical applications in NHS CAMHS and related services.

Research Questions

1. How effectively does MAVIC-R facilitate communication about DSEs with young people?
2. Which components are most helpful in exploring personal appraisals and meanings of DSEs?
3. How do young people, caregivers, and professionals perceive the tool's utility?
4. What are the practical and therapeutic implications of its use in CAMHS?

Research Design

Phase 1: MAVIC-R Refinement
Discussions with the ChUSE PPIE groups will inform updates to the existing MAVIC, expanding its scope from voice-hearing to multisensory experiences. Insights from these consultations will guide revisions to language and content.

Phase 2: Validation Study
A mixed-methods design will assess the psychometric properties and practical acceptability of the MAVIC-R:

For peer review only

- **Quantitative**: An online survey with young people will assess item properties and factor structure via Exploratory Factor Analysis (EFA).
- **Qualitative**: Mental health professionals will trial the tool in practice and provide feedback through interviews on its implementation and acceptability.

Participant Recruitment

- Up to 150 Young People (Aged 8–18) with lived experience of USEs, recruited through professional and lived experience networks, such as Voice Collective.
- 10 Parents/Caregivers of young people with USEs
- 10 Multiservice Professionals working with young people experiencing USEs

Inclusion Criteria

- Young people aged 8–18, fluent in conversational English, with personal experiences of one or more USEs
- Caregivers with current or past experience of supporting a young person with USEs
- Professionals with regular experience working with young people experiencing USEs

Exclusion Criteria

- Young people outside the specified age range, or whose current mental health prevents participation
- Professionals/caregivers without relevant experience
- Autistic young people whose USEs are solely attributed to sensory processing differences

Recruitment Process

Participants will be recruited via the RRDN, NHS networks, peer organisations (e.g. Voice Collective), and professional contacts. The same referral process as the wider ChUSE Trial will be followed, with practitioners completing brief referral forms with consent.

Consent/Assent

Participants will provide electronic consent via Qualtrics for surveys or audio-recorded consent for in-person interviews. For those under 16, caregiver consent and participant assent will be obtained.

Data Collection and Analysis

Audio-recorded interviews and focus groups will be transcribed and analysed thematically (Braun & Clark, 2022) using NVivo. Qualitative survey data will also be analysed thematically, and quantitative survey data will be analysed in SPSS. Data will be screened for missingness, item correlations examined, and sampling adequacy assessed via KMO and Bartlett's tests. EFA using Maximum Likelihood extraction with oblique rotation will determine factor structure, guided by scree plots, Kaiser's criterion, and parallel analysis. Reliability will be tested via Cronbach's alpha and item-total correlations.

Expected Outcomes and Dissemination

For peer review only

If validated, the MAVIC-R will provide a practical, non-stigmatising tool for facilitating discussions about USEs with young people, supporting tailored, person-centred care. Its use could foster more holistic, youth-informed care pathways within CAMHS and other youth services. Findings will be disseminated via peer-reviewed publications, stakeholder summaries, and multimedia resources for young people, families, and professionals. This study will also enable the inclusion of a validated, youth-specific measure in a future definitive ChUSE Trial.

Trial Endpoints, Ethical and Regulatory Considerations, and Data Management

The intervention period for this trial is scheduled to conclude by 1st March 2026, with all data collection for the trial completed by 1st June 2026. The nested validation study will be completed by the 1st December 2026. Prior to study commencement, the protocol, consent materials, and study documents will obtain favourable NHS REC and HRA approvals, with local Capacity and Capability agreements from participating NHS Trusts. The study will adhere to the Declaration of Helsinki, Good Clinical Practice, and the UK Policy Framework for Health and Social Care Research (2017).

Regulatory compliance will be maintained through appropriate approvals for all participating sites, timely submission of amendments, and regular oversight from the Sponsor and TSC. Protocol deviations will be formally logged and corrective actions agreed upon collaboratively.

Risk management procedures will include bespoke SOPs for safeguarding, clear limits of confidentiality, procedures for managing participant distress, and strict lone working and infection control policies for face-to-face contact.

Adverse events (AEs) and serious adverse events (SAEs) will be monitored closely, recorded according to established SOPs, and reviewed for severity, relatedness, expectedness, and seriousness. SAEs that are unexpected and related will be reported to the REC and Sponsor in line with standard HRA timelines.

Data management will comply with the Data Protection Act 2018 and GDPR. All personal data will be pseudonymised, securely stored, and deleted or anonymised at study end. Research data will be stored on encrypted servers, with access restricted to authorised study personnel. Final datasets and supporting materials will be archived securely with the Sponsor.

Ethics and Dissemination

This study addresses a significant clinical need in the care of young people with DSEs in CAMHS. Given the high prevalence of emotional distress and risk-related difficulties among this population, alongside existing service pressures and limitations in evidence-based care pathways, this trial has the potential to inform urgently needed improvements in clinical practice. Currently, no psychological interventions have established evidence specifically for treating DSEs for young people, though a focus on coping strategies has been suggested as a useful approach (Denno, et al., 2022; Hayward, et al., 2022). Our research so far has indicated young people (e.g., Parry & Varese, 2020) and parents (e.g., Parry & Varese, 2021) want more information and support from service providers on coping strategies and skills for managing at home, which the ChUSE intervention has the potential to offer.

For peer review only

The protocol incorporates several methodological innovations, including the integration of remote and in-person data collection, rigorous adverse event monitoring frameworks tailored for non-CTIMP trials, and a comprehensive safeguarding strategy embedded within standard operating procedures. The trial also benefits from a mixed-methods design, enabling exploration of both quantitative outcomes and qualitative insights into participant and clinician experiences, enhancing the relevance and applicability of findings to frontline services.

Public engagement has been central to the development and design of this study, with young people, families, and practitioners contributing to intervention refinement, study materials, and data collection processes. Their input ensures that the research is sensitive to the needs and preferences of those it aims to support, and that dissemination plans are inclusive and accessible. We will endeavour to adopt the multisensory approach of the ChUSE intervention into the development of multisensory and multimedia translational outputs from the study, suitable and engaging for a range of audiences, in collaboration with our advisory groups.

If successful, this study has the potential to inform clinical guidelines, contribute to workforce development through training and supervision models, and ultimately improve the quality and safety of services offered to young people in NHS mental health settings. Dissemination activities will be designed to engage a range of audiences, including young people, families, the public, and professionals across health, education, and voluntary sectors. A co-produced multimedia portfolio, including short animations, infographics, podcasts, and youth-friendly summaries, will support accessible communication of findings. These outputs will be developed in collaboration with the LEAG and shared through digital platforms, schools, CAMHS services, and third-sector networks. In parallel, findings will be disseminated through academic publications, policy briefings, and stakeholder events to inform national guidance and service development, with the aim of influencing policy and practice in child and adolescent mental health care.

For peer review only

Declarations: Ethics approval and consent to participate: Approved by NHS Research Ethics Committee (Ref: IRAS 327343). This study was funded by the National Institute for Health and Care Research (NIHR). The views expressed are those of the authors and not necessarily those of the NIHR or the Department of Health and Social Care.

Consent for publication: Not applicable.

Availability of data and materials: The datasets generated and/or analysed during this study will be available from the corresponding author on reasonable request.

Competing interests: The authors declare that they have no competing interests.

Funding: National Institute for Health and Care Research (NIHR) Research for Patient Benefit Programme (NIHR205326).

Author contributions: SP and FV conceived the study and developed the protocol. FM contributed to intervention development and PPI coordination. TM, SK, and DF provided methodological expertise. LAC advised on statistical analyses. GS and AT advised on health economics. All authors reviewed and approved the final manuscript.

Acknowledgements: The authors thank the young people, parents, CAMHS practitioners, and members of the Lived Experience Advisory Group who contributed to the development of this protocol.

For peer review only

References

Abajobir, A. A., Kisely, S., Scott, J. G., Williams, G., Clavarino, A., Strathearn, L., & Najman, J. M. (2017). Childhood maltreatment and young adulthood hallucinations, delusional experiences, and psychosis: A longitudinal study. *Schizophrenia Bulletin, 43*(5), 1045–1055. https://doi.org/10.1093/schbul/sbx091

Alderson-Day, B., Moseley, P., Common, S., Deamer, F., Dodgson, G., Fernyhough, C., & Woods, A. (2021). Voice-hearing and personification: Characterizing social qualities of auditory verbal hallucinations in early psychosis. *Schizophrenia Bulletin, 47*(1), 228–236. https://doi.org/10.1093/schbul/sbaa127

Alderson-Day, B., Woods, A., Moseley, P., Common, S., Deamer, F., Dodgson, G., & Fernyhough, C. (2023). Varieties of felt presence? Three surveys of presence phenomena and their relations to psychopathology. *Psychological Medicine, 53*(8), 3692–3700. https://doi.org/10.1017/S0033291723000663

Al-Ghabban, A. (2018). A compassion framework: The role of compassion in schools in promoting well-being and supporting the social and emotional development of children and young people. *Pastoral Care in Education, 36*(3), 176–188. https://doi.org/10.1080/02643944.2018.1482875

Aynsworth, C., Rolinson, J., Pervez, M., Collerton, D., & Dudley, R. (2023). What is the frequency and nature of visual hallucinations in non-clinical participants? *Psychology and Psychotherapy: Theory, Research and Practice, 96*(2), 281–295. https://doi.org/10.1111/papt.12387

Baumeister, D., Sedgwick, O., Howes, O., & Peters, E. (2017). Auditory verbal hallucinations and continuum models of psychosis: A systematic review of the healthy voice-hearer literature. *Clinical Psychology Review, 51*, 125–141. https://doi.org/10.1016/j.cpr.2016.10.005

Begemann, M. J. H., Sommer, I. E., Brand, R. M., Oomen, P. P., Jongeneel, A., Berkhout, J., ... & Bell, I. H. (2022). Auditory verbal hallucinations and childhood trauma subtypes across the psychosis continuum: A cluster analysis. *Cognitive Neuropsychiatry, 27*(2–3), 150–168. https://doi.org/10.1080/13546805.2021.2025367

Bluth, K. (2020). *The self-compassionate teen: Mindfulness and compassion skills to conquer your critical inner voice*. New Harbinger Publications.

Camerini, B. A., Baker, K. K., Gadelha, A., & Margolis, R. L. (2021). Hearing voices and seeing things: Symptoms of anxiety misconstrued as evidence of schizophrenia in an adolescent. *Journal of Psychiatric Practice®, 27*(3), 232–238. https://doi.org/10.1097/PRA.0000000000000532

Coughlan, H., Humphries, N., Clarke, M. C., Healy, C., & Cannon, M. (2022). Psychotic-like experiences? Trajectories and typologies of hallucinations and delusions from early adolescence to early adulthood in a population-based sample of Irish youth. *Irish Journal of Psychological Medicine, 39*(2), 207–222. https://doi.org/10.1017/ipm.2021.67

Coughlan, H., Healy, C., Humphries, N., Clarke, M., Kelleher, I., & Cannon, M. (2022). Phenomenological characteristics and explanations of unusual perceptual experiences, thoughts and beliefs in a population sample of early adolescents. *Irish Journal of Psychological Medicine, 39*(2), 173–184. https://doi.org/10.1017/ipm.2021.68

For peer review only

da Cunha Koch, C., Coughlan, H., & Cannon, M. (2024). Representations of hallucinations and dissociation in young adult literature: Using literature to challenge stigma about psychosis. *Irish Journal of Psychological Medicine, 41*(1), 125–131. https://doi.org/10.1017/ipm.2023.33

D'Agnano, D., Lo Cascio, S., Correnti, E., Raieli, V., & Sciruicchio, V. (2023). A narrative review of visual hallucinations in migraine and epilepsy: Similarities and differences in children and adolescents. *Brain Sciences, 13*(4), 643.

Denno, P., Wallis, S., Caldwell, K., Ives, J., Wood, S. J., Broome, M. R., ... & Upthegrove, R. (2022). Listening to voices: Understanding and self-management of auditory verbal hallucinations in young adults. *Psychosis, 14*(3), 281–292. https://doi.org/10.1080/17522439.2022.2066391

Di Luzio, M., Pontillo, M., Di Vincenzo, C., Bellantoni, D., Demaria, F., & Vicari, S. (2022). Hallucinations in the child and adolescent "ultra-high risk" population: A systematic review. *Schizophrenia Bulletin Open, 3*(1), sgac068.

Dodgson, G., Gordon, E., Bone, A., Bowe, S., McCarthy-Jones, S., & Sommer, I. E. (2020). Voices in the head: A qualitative study exploring the factors associated with distress and benefit. *Schizophrenia Bulletin, 46*(5), 1252–1261. https://doi.org/10.1093/schbul/sbaa041

Domínguez, T., Nieto, L., Fresán, A., Sheinbaum, T., & Robles, R. (2025). The association of bullying and self-esteem with psychotic-like experiences and clinical high risk for psychosis symptoms. *Child and Adolescent Psychiatry and Mental Health*, *19*(1), 51.

Dorahy, M. J., Shannon, C., Seagar, L., Corr, M., Stewart, K., Hanna, D., & Mulholland, C. (2009). Auditory hallucinations in individuals with posttraumatic stress disorder and borderline personality disorder: Similarities and differences in phenomenology and psychological processes. *Journal of Nervous and Mental Disease, 197*(12), 877–881. https://doi.org/10.1097/NMD.0b013e3181c29968

Durbin, A., Lin, E., Layne, C., Reid, G. J., & Steele, M. (2022). Psychotic-like experiences and suicidal ideation and behaviors in Canadian adolescents and young adults. *Early Intervention in Psychiatry, 16*(3), 304–311. https://doi.org/10.1111/eip.13152

Dyer, K. F. W., Bell, R., McGlade, T., & McIlwain, D. (2022). Childhood trauma and voice hearing: A meta-analysis. *Psychological Medicine, 52*(4), 587–597. https://doi.org/10.1017/S0033291720002349

Eldridge, S. M., Chan, C. L., Campbell, M. J., Bond, C. M., Hopewell, S., Thabane, L., & Lancaster, G. A. (2016). CONSORT 2010 statement: Extension to randomised pilot and feasibility trials. *BMJ, 355*, i5239. https://doi.org/10.1136/bmj.i5239

Fazel, S., Doll, H., & Långström, N. (2008). Mental disorders among adolescents in juvenile detention and correctional facilities: A systematic review and metaregression analysis of 25 surveys. *Journal of the American Academy of Child and Adolescent Psychiatry, 47*(9), 1010–1019. https://doi.org/10.1097/CHI.0b013e31817eecf3

Fernyhough, C., de Boer, J., Davis, P. E., Schneider, M., Moriguchi, Y., Alderson-Day, B., … Jardri, R. (2025). Cognitive-developmental mechanisms in hallucinations. *Schizophrenia Bulletin, 51*(Supplement_3), S261–S272.

Fusar-Poli, P., Cappucciati, M., Rutigliano, G., Lee, T. Y., Beveridge, R., Bonoldi, I., ... & McGuire, P. (2015). At risk or not at risk? A meta-analysis of the prognostic accuracy of psychometric

interviews for psychosis prediction. *World Psychiatry, 14*(3), 322–332. https://doi.org/10.1002/wps.20250

Garwood, L., Dodgson, G., Brown, M., & Hunter, E. C. M. (2015). Voice hearing in adolescence: A critique of the literature and directions for future research. *Child and Adolescent Mental Health, 20*(1), 48–54. https://doi.org/10.1111/camh.12062

Gearing, R. E., Schwalbe, C. S. J., MacKenzie, M. J., Brewer, K. B., Ibrahim, R. W., Olimat, H. S., ... & Mian, I. A. (2015). Adaptation and translation of mental health interventions in Middle Eastern Arab countries: A systematic review of barriers to and strategies for effective treatment implementation. *International Journal of Social Psychiatry, 61*(6), 505–513. https://doi.org/10.1177/0020764015573085

Gibson, L. E., Alloy, L. B., Ellman, L. M., & Park, S. (2023). Auditory verbal hallucinations in the general population: Clarifying relations to dissociation and trauma. *Psychological Bulletin, 149*(4), 337–369. https://doi.org/10.1037/bul0000375

Goss, K., & Gilbert, P. (2002). Eating disorders, shame and pride: A cognitive-behavioural functional analysis. In P. Gilbert & J. Miles (Eds.), *Body shame: Conceptualisation, research and treatment* (pp. 219–255). Routledge.

Gosse, C. (2023). "Is this my voice or someone else's?" Exploring the role of compassion and emotion regulation in adolescents and young people who hear voices [Doctoral dissertation, University of Liverpool]. https://livrepository.liverpool.ac.uk/3162029/

Gosse, C., Corcoran, R., & Dudley, R. (2021). The role of emotion regulation in the experience of auditory hallucinations and distress in young people. *Psychosis, 13*(3), 193–202. https://doi.org/10.1080/17522439.2021.1942945

Hancock, K., Lavis, A., & Greenwood, K. (2022). A qualitative evidence synthesis of young people's experiences of hearing voices and the voices' impact on relationships. *Psychosis, 14*(4), 264–280. https://doi.org/10.1080/17522439.2022.2095584

Hayward, M., Berry, K., & Ashton, A. (2011). Applying interpersonal theories to the understanding of and therapy for auditory hallucinations: A review of the literature and directions for further research. *Clinical Psychology Review, 31*(8), 1313–1323. https://doi.org/10.1016/j.cpr.2011.07.001

Hayward, M., Denney, J., Vaughan, S., & Fowler, D. (2008). The voice and me: A constructivist cognitive therapy for persistent auditory hallucinations. *Behavioural and Cognitive Psychotherapy, 36*(5), 491–502. https://doi.org/10.1017/S1352465808004480

Hayward, M., Jones, A. M., & Dunn, G. (2017). The impact of compassionate mind training on adults who hear malevolent voices: A feasibility study. *Psychology and Psychotherapy: Theory, Research and Practice, 90*(2), 178–193. https://doi.org/10.1111/papt.12099

Hayward, M., Overton, J., Dorey, T., & Denney, J. (2009). Relating therapy for people who hear voices: A case series. *Clinical Psychology and Psychotherapy, 16*(3), 216–227. https://doi.org/10.1002/cpp.615

Healy, C., Brannigan, R., Dooley, N., Coughlan, H., Clarke, M., Kelleher, I., & Cannon, M. (2019). Childhood and adolescent psychotic experiences and risk of mental disorder: A systematic

For peer review only

review and meta-analysis. *Psychological Medicine, 49*(10), 1589–1599. https://doi.org/10.1017/S0033291718002584

Heriot-Maitland, C., McCarthy-Jones, S., Longden, E., & Gilbert, P. (2019). Compassion focused approaches to working with distressing voices. *Frontiers in Psychology, 10*, 152. https://doi.org/10.3389/fpsyg.2019.00152

Hickman, C., & Fulford, K. W. M. (2019). Values-based practice in psychosis: A case example. In C. Sandford, R. W. P. Bingham, & G. G. Habermann (Eds.), *Handbook of Philosophy and Psychiatry* (pp. 1279–1292). Springer. https://doi.org/10.1007/978-3-319-51726-6_93-1

Iudici, A., & Salvini, A. (2016). Hearing voices and the relational world: Pathways to understanding the experience. *Journal of Constructivist Psychology, 29*(2), 146–160. https://doi.org/10.1080/10720537.2015.1019993

Jones, S. R., Fernyhough, C., de-Wit, L., & Meins, E. (2008). A message in the medium? Assessing the reliability of voice-hearing in young people using Experience Sampling Methodology. *Journal of Youth and Adolescence, 37*(2), 129–143. https://doi.org/10.1007/s10964-007-9173-0

Kapur, P., Hayes, D., Waddingham, R., Hillman, S., Deighton, J., & Midgley, N. (2014). The experience of engaging with mental health services among young people who hear voices and their families: a mixed methods exploratory study. *BMC Health Services Research*, *14*(1), 527.

Kelleher, I. (2025). *Annual Research Review: Psychosis in children and adolescents: Key updates from the past 2 decades on psychotic disorders, psychotic experiences, and psychosis risk.* Journal of Child Psychology and Psychiatry, 66(4), 460–476. https://doi.org/10.1111/jcpp.14088

Kelleher, I., Keeley, H., Corcoran, P., Lynch, F., Fitzpatrick, C., Devlin, N., ... & Cannon, M. (2012). Clinicopathological significance of psychotic experiences in non-psychotic young people: Evidence from four population-based studies. *British Journal of Psychiatry, 201*(1), 26–32. https://doi.org/10.1192/bjp.bp.111.101543

Kelleher, I., Lynch, F., Harley, M., Molloy, C., Roddy, S., Fitzpatrick, C., & Cannon, M. (2012). Psychotic symptoms in adolescence index risk for suicidal behavior: Findings from 2 population-based case-control clinical interview studies. *Archives of General Psychiatry, 69*(12), 1277–1283. https://doi.org/10.1001/archgenpsychiatry.2012.164

Kennedy, M., Gabbidon, J., & Hill, L. (2018). 'I have to prove myself all the time': The experience of young people of colour living with psychosis. *Clinical Child Psychology and Psychiatry, 23*(1), 65–78. https://doi.org/10.1177/1359104517725672

Larøi, F. (2012). How do auditory verbal hallucinations in patients differ from those in non-patients? *Frontiers in Human Neuroscience, 6*, 25. https://doi.org/10.3389/fnhum.2012.00025

Larøi, F., Luhrmann, T. M., Bell, V., Christian, W. A., Deshpande, S., Fernyhough, C., ... & Woods, A. (2014). Culture and hallucinations: Overview and future directions. *Schizophrenia Bulletin, 40*(S4), S213–S220. https://doi.org/10.1093/schbul/sbu012

Lavis, A., & Winter, R. (2020). 'Voice-hearing is not madness': A qualitative study exploring young people's perceptions of voice-hearing. *Child and Adolescent Mental Health, 25*(1), 40–46. https://doi.org/10.1111/camh.12338

For peer review only

Lawrence, C., Jones, J., & Cooper, M. (2010). Hearing voices in a non-psychiatric population. *Behavioural and Cognitive Psychotherapy, 38*(3), 363–373. https://doi.org/10.1017/S1352465809990702

Lee, E. C., A. L. Whitehead, R. M. Jacques, and S. A. Julious. 2014. "The Statistical Interpretation of Pilot Trials: Should Significance Thresholds Be Reconsidered?" *BMC Medical Research* Methodology 14, no. 1: 1–8. https://doi.org/10.1186/1471-2288-14-41.

Longden, E., Corstens, D., & Dillon, J. (2013). Recovery, discovery and revolution: The work of Intervoice and the Hearing Voices Movement. *Openmind, 174*, 6–8. https://www.intervoiceonline.org/wp-content/uploads/2011/02/Recovery-Discovery-and-Revolution-Longden-et-al.pdf

Longden, E., Madill, A., & Waterman, M. G. (2012). Dissociation, trauma, and the role of lived experience: Toward a new conceptualization of voice hearing. *Psychological Bulletin, 138*(1), 28–76. https://doi.org/10.1037/a0026985

Luhrmann, T. M. (2022). *How God becomes real: Kindling the presence of invisible others*. Princeton University Press.

Luhrmann, T. M., Padmavati, R., Tharoor, H., & Osei, A. (2015). Differences in voice-hearing experiences of people with psychosis in the U.S.A., India and Ghana: Interview-based study. *British Journal of Psychiatry, 206*(1), 41–44. https://doi.org/10.1192/bjp.bp.113.139048

McCarthy-Jones, S. (2012). *Hearing voices: The histories, causes and meanings of auditory verbal hallucinations*. Cambridge University Press.

McCarthy-Jones, S., & Longden, E. (2015). Auditory verbal hallucinations in schizophrenia and post-traumatic stress disorder: Common phenomenology, common cause, common interventions? *Frontiers in Psychology, 6*, 1071. https://doi.org/10.3389/fpsyg.2015.01071

McCarthy-Jones, S., Trauer, T., Mackinnon, A., Sims, E., Thomas, N., & Copolov, D. L. (2014). A new phenomenological survey of auditory hallucinations: Evidence for subtypes and implications for theory and practice. *Schizophrenia Bulletin, 40*(1), 231–235. https://doi.org/10.1093/schbul/sbs156

McPin Hearing Voices Project. (2018). *Understanding voices: A co-produced website and resource on voice-hearing experiences*. https://www.understandingvoices.com

Moskowitz, A., Read, J., Farrelly, S., & Williams, O. (2009). Are psychotic symptoms traumatic in themselves? *Journal of Traumatic Stress, 22*(1), 74–78. https://doi.org/10.1002/jts.20325

Moskowitz, A., Read, J., & Longden, E. (2017). The role of trauma in psychosis: A review of current evidence. In J. Read, & J. Dillon (Eds.), *Models of madness: Psychological, social and biological approaches to psychosis* (2nd ed., pp. 99–124). Routledge.

Moskowitz, A., Schäfer, I., & Dorahy, M. J. (Eds.). (2019). *Psychosis, trauma and dissociation: Evolving perspectives on severe psychopathology*. John Wiley & Sons.

National Institute for Health and Care Excellence (NICE). (2016). *Psychosis and schizophrenia in children and young people: Recognition and management* (NICE guideline [CG155]). https://www.nice.org.uk/guidance/cg155

For peer review only

Nishiura, S., Miyawaki, D., Hirai, K., Sukigara, A., Kakishita, Y., & Inoue, K. (2024). Association between hallucinations and sensory processing difficulties in children and adolescents. *Frontiers in Psychiatry, 15*, 1472328.

Parry, S., Lobban, F., & Jones, S. (2012). Are voices related to beliefs about morality? *Psychosis, 4*(1), 46–58. https://doi.org/10.1080/17522439.2011.582115

Perona-Garcelán, S., Cuevas-Yust, C., García-Montes, J. M., Álvarez-Moral, M. T., López-Jiménez, A. M., & Pérez-Álvarez, M. (2012). Relationship between self-focused attention and dissociation in patients with and without auditory hallucinations. *The Journal of Nervous and Mental Disease, 200*(4), 349–355. https://doi.org/10.1097/NMD.0b013e31824cb278

Read, J., Fosse, R., Moskowitz, A., & Perry, B. D. (2014). The traumagenic neurodevelopmental model of psychosis revisited. *Neuropsychiatry, 4*(1), 65–79. https://doi.org/10.2217/npy.13.89

Read, J., Van Os, J., Morrison, A. P., & Ross, C. A. (2005). Childhood trauma, psychosis and schizophrenia: A literature review with theoretical and clinical implications. *Acta Psychiatrica Scandinavica, 112*(5), 330–350. https://doi.org/10.1111/j.1600-0447.2005.00634.x

Roberts, S., & Parry, S. (2023). Girl's and women's experiences of seeking mental health support for symptoms associated with psychosis. A narrative review. *Clinical Psychology & Psychotherapy*, *30*(2), 294-301.

Romme, M., & Escher, S. (2000). *Making sense of voices: A guide for mental health professionals working with voice-hearers*. Mind Publications.

Smailes, D., Meins, E., & Fernyhough, C. (2015). Self-reflection and auditory hallucinations in non-clinical individuals: A study of the role of inner speech. *Consciousness and Cognition, 31*, 104–112. https://doi.org/10.1016/j.concog.2014.10.006

Tang, S., Xie, Y., Wang, X., You, S., Fu, Y., & Wu, D. (2025). The prevalence and influencing factors of psychotic-like experiences in children and adolescents: A meta-analysis and systematic review. *European Child & Adolescent Psychiatry*, 1–18.

Teare, M. D., Dimairo, M., Shephard, N., Hayman, A., Whitehead, A., & Walters, S. J. (2014). Sample size requirements to estimate key design parameters from external pilot randomised controlled trials: A simulation study. *Trials*, *15*(264). https://doi.org/10.1186/1745-6215-15-264

Thompson, A., Nelson, B., & Yung, A. R. (2011). Predictive validity of clinical high-risk for psychosis diagnosis in helping-seeking youth: A meta-analysis. *Schizophrenia Research, 125*(2-3), 54–62. https://doi.org/10.1016/j.schres.2010.07.017

UK Government. (1989). *Children Act 1989*. https://www.legislation.gov.uk/ukpga/1989/41/contents

Whitehead, A. L., Julious, S. A., Cooper, C. L., & Campbell, M. J. (2016). Estimating the sample size for a pilot randomised trial to minimise the overall trial sample size for the external pilot and main trial for a continuous outcome variable. *Statistical Methods in Medical Research*, *25*(3), 1057–1073. https://doi.org/10.1177/0962280215588241

Woods, A., Jones, N., Alderson-Day, B., Callard, F., & Fernyhough, C. (2015). Experiences of hearing voices: Analysis of a novel phenomenological survey. *The Lancet Psychiatry, 2*(4), 323–331. https://doi.org/10.1016/S2215-0366(15)00006-1

For peer review only

Supplementary Materials

CONSORT Checklist

Item	CONSORT Item (Pilot/Feasibility Extension)	Reported?	Section / Location in Protocol
**TITLE & ABSTRACT**			
**1a**	Identification as a pilot or feasibility randomised trial in the title	**Yes**	Title – "Randomised Controlled Feasibility Trial Protocol"
**1b**	Structured summary appropriately detailing pilot design, methods, feasibility outcomes	**Yes**	Abstract – structured with Introduction, Methods, Ethics, Dissemination
**INTRODUCTION**			
**2a**	Scientific background; rationale for future definitive trial; reasons for conducting pilot	**Yes**	Introduction / Background – unmet need, prevalence, lack of evidence-based interventions; justification for early intervention
**2b**	Specific objectives or research questions	**Yes**	Aims & Objectives – primary feasibility objectives and secondary feasibility questions
**METHODS**			
**3a**	Description of pilot trial design (parallel, allocation ratio, etc.)	**Yes**	Trial Design – single-blind, parallel-group, 1:1 randomisation
**3b**	Important changes to methods after commencement	**N/A**	Protocol (pre-trial) – not applicable
**4a**	Eligibility criteria	**Yes**	Participants & Recruitment – age 8–16; CAMHS involvement; self-identified DSEs; screening using MAVIC/MUSEQ
**4b**	Settings and locations where data collected	**Yes**	Trial Setting – three NHS CAMHS Trusts in Greater Manchester

**4c**	How participants were identified, approached, consented	**Yes**	Participant Identification & Recruitment – MDT screening, clinician referral, consent/assent procedures
**5**	Interventions described with sufficient detail for replication	**Yes**	Interventions – detailed TAU description; full child and parent session structure; fidelity procedures
**6a**	Prespecified assessments/measurements for each pilot objective	**Yes**	Measures; Primary & Secondary Feasibility Outcomes – recruitment, retention, adherence, fidelity, safety
**6b**	Changes to outcomes after the trial begins	**N/A**	Protocol – pre-trial
**6c**	Prespecified **progression criteria** (“Go/No-Go”) for future definitive trial	**Yes**	**Progression Criteria** – explicit quantitative thresholds for recruitment, adherence, retention, parent session uptake, SAE thresholds
**7a**	Rationale for sample size in a pilot trial	**Yes**	Sampling & Recruitment Targets – 60 dyads based on pilot trial recommendations
**7b**	Explanation of interim analyses and stopping rules	**Yes**	Sampling & Recruitment Targets – month-12 recruitment review and contingencies
**8a**	Method used to generate allocation sequence	**Yes**	Randomisation & Blinding – web-based pseudo-random sequence
**8b**	Type of randomisation; restrictions (block size etc.)	**Yes**	Randomisation & Blinding – permuted blocks of varying size
**9**	Allocation concealment mechanism	**Yes**	Randomisation & Blinding – independent sealed envelope system; concealed until assignment

For peer review only

**10**	Who generated the allocation, who enrolled participants, who assigned interventions	**Yes**	Randomisation & Blinding – CI/therapist enters ID; system assigns
**11a**	Blinding: who was blinded and how	**Yes**	Randomisation & Blinding – researchers blinded; CI/therapists unblinded; procedures to avoid unblinding
**11b**	Similarity of interventions, if relevant	**Yes**	Interventions – contrast between TAU and ChUSE+TAU explained
**12a**	Methods for addressing pilot objectives (quantitative/qualitative)	**Yes**	Analysis; Process Evaluation – descriptive statistics; Framework Method
**12b**	Additional analyses	**N/A**	No additional analyses prespecified for protocol stage
**RESULTS *(not applicable for protocol papers)***			
**13–19a**	Participant flow, recruitment dates, baseline data, outcomes, harms	**N/A**	Will be reported in final results; safety plan included in Follow-Up Assessments
**DISCUSSION *(for final results papers)***			
**20–22a**	Pilot limitations, generalisability, interpretation, progression implications	**N/A**	Will be reported in final trial report
**OTHER INFORMATION**			
**23**	Trial registration	**Yes**	Abstract – ISRCTN12245618
**24**	Protocol access	**Yes**	This document is the protocol; also registered via ISRCTN
**25**	Funding	**Yes**	Declarations – NIHR Research for Patient Benefit (NIHR205326)

For peer review only

**26**	Ethical approval	**Yes**	Ethics – NHS REC approval (IRAS 327343)

SPIRIT 2013 Checklist

SECTION 1: ADMINISTRATIVE INFORMATION

**Item**	**SPIRIT Description**	**Addressed in Protocol?**	**Location in Protocol**
**1. Title**	Descriptive title identifying study design, population, interventions	**Yes**	Title page
**2a. Trial Registration**	Trial identifier & registry	**Yes**	Abstract – ISRCTN12245618
**2b. WHO Data Set**	All items from WHO Trial Registration Data Set	**Yes (embedded)**	Trial Registration section
**3. Protocol Version**	Date & version	**Yes**	Cover page (version at submission)
**4. Funding**	Sources & types of support	**Yes**	Declarations – Funding (NIHR RfPB NIHR205326)
**5a. Roles & Responsibilities**	Contributors and roles	**Yes**	Authors' Contributions section
**5b. Sponsor**	Name & contact of sponsor	**Yes**	Governance sections
**5c. Committees**	Steering, advisory, oversight groups	**Yes**	PPIE & Governance (LEAG, Parent Group, Stakeholder Advisory Board, TSC)

SECTION 2: INTRODUCTION

**Item**	**SPIRIT Description**	**Addressed?**	**Location**
**6. Background & Rationale**	Scientific justification, uncertainty, need for feasibility trial	**Yes**	Background & Introduction
**7. Objectives**	Specific primary & secondary objectives	**Yes**	Aims & Objectives

For peer review only

**8. Trial Design**	Description of design, allocation, framework	**Yes**	Trial Design (single-blind, 2-arm, 1:1 RCT with process evaluation)

SECTION 3: METHODS – PARTICIPANTS, INTERVENTIONS & OUTCOMES

Participants

**Item**	**Description**	**Addressed?**	**Location**
**9. Study Setting**	Where data collected	**Yes**	Trial Setting (3 NHS CAMHS Trusts in GM)
**10. Eligibility Criteria**	Inclusion/exclusion	**Yes**	Eligibility Criteria section

Interventions

**Item**	**Description**	**Addressed?**	**Location**
**11a. Interventions**	Detailed descriptions for replication	**Yes**	Interventions section (Child sessions; Parent coaching; TAU)
**11b. Discontinuation Criteria**	When interventions may be stopped	**Yes**	Consent/Withdrawal; SPIRIT discontinuation criteria
**11c. Adherence Strategies**	Strategies to improve fidelity & adherence	**Yes**	Fidelity & Process Monitoring; Adherence checklists
**11d. Concomitant Care**	Permitted/prohibited treatments	**Yes**	TAU description – routine care not restricted

Outcomes

**Item**	**Description**	**Addressed?**	**Location**
**12. Outcomes**	Clearly defined primary (feasibility) & secondary outcomes	**Yes**	Primary & Secondary Feasibility Outcomes; Measures
**13. Participant Timeline**	Expected schedule of enrolment, interventions, and assessments	**Yes**	Follow-Up Assessments; Tables 1–3; Trial Design

Sample Size & Recruitment

**Item**	**Description**	**Addressed?**	**Location**

For peer review only

**14. Sample Size**	Rationale for numbers in pilot	**Yes**	Sampling and Recruitment Targets (n=60 dyads)
**15. Recruitment**	Strategies for achieving adequate enrolment	**Yes**	Participant Identification & Recruitment; MDT meetings; recruitment targets

SECTION 4: METHODS – ASSIGNMENT OF INTERVENTIONS

**Item**	**Description**	**Addressed?**	**Location**
**16a. Sequence Generation**	How randomisation sequence is generated	**Yes**	Randomisation & Blinding – permuted blocks
**16b. Allocation Concealment**	Allocation mechanism	**Yes**	Concealed via web-based system
**16c. Implementation**	Who generates/enrols/assigns	**Yes**	CI/therapist enters ID; system assigns
**17a. Blinding**	Who is blinded and how	**Yes**	Research workers blinded; CI/therapist unblinded
**17b. Unblinding Procedures**	Circumstances requiring unblinding	**Yes**	Randomisation & Blinding – breach documentation

SECTION 5: METHODS – DATA COLLECTION, MANAGEMENT, ANALYSIS

Data Collection

**Item**	**Description**	**Addressed?**	**Location**
**18a. Data Collection Plan**	Detailed plans for assessments	**Yes**	Measures; Follow-up Assessments
**18b. Participant Retention**	Strategies to retain participants and manage missing data	**Yes**	Multi-mode follow-up; repeated contact attempts

Data Management

**Item**	**Description**	**Addressed?**	**Location**
**19. Data Management**	Data quality, collection, entry, coding, storage	**Yes**	Data Management & GDPR section

Statistical Methods

Item	Description	Addressed?	Location
**20a. Primary Feasibility Analyses**	Methods for primary outcomes	**Yes**	Analysis section – descriptive statistics
**20b. Additional Analyses**	Subgroup/adjusted analyses	**Not applicable (protocol stage)**	–
**20c. Analysis Population**	Handling of non-adherence & missing data	**Yes**	Data handling detail in Analysis section

SECTION 6: ETHICS & DISSEMINATION

Item	Description	Addressed?	Location
**21. Research Ethics Approval**	REC approval	**Yes**	Declarations – ethics approval IRAS 327343
**22. Protocol Amendments**	Communication plans	**Yes**	Governance & Regulatory Compliance
**23. Consent/Assent**	Process for obtaining informed consent	**Yes**	Consent & Assent Procedures
**24. Confidentiality**	Protection of personal data	**Yes**	Data Management; GDPR compliance
**25. Declaration of Interests**	Investigator conflicts	**Yes**	Declarations – Competing Interests
**26. Access to Data**	Who will have access	**Yes**	Data management; access limited to authorised staff
**27. Ancillary & Post-Trial Care**	Care for harm and compensation	**Yes**	Safety & AE/SAE procedures
**28. Dissemination**	Plans for reporting and sharing findings	**Yes**	Dissemination section – publications, multimedia, stakeholder events

SECTION 7: APPENDICES

Item	Description	Addressed?	Location
**29. Consent Materials**	Information sheets & consent forms	**Yes**	Mentioned; provided in study materials pack

For peer review only

**30. Biological Specimens**	Collection/storage of samples	**Not applicable**	No biological sampling

For peer review only

BMJ Open

The ChUSE Trial - Improving Access to Psychological Therapies for Children with Distressing Sensory Experiences in NHS CAMHS: A Randomised Controlled Feasibility Trial Protocol

Journal:	*BMJ Open*
Manuscript ID	bmjopen-2026-119727.R1
Article Type:	Protocol
Date Submitted by the Author:	04-Jun-2026
Complete List of Authors:	Parry, Sarah ; Pennine Care NHS Foundation Trust, Young People's Mental Health Research Centre; The University of Manchester, Division of Psychology and Mental Health | School of Health Sciences | Faculty of Biology<br>Carr, Sarah; Greater Manchester Mental Health NHS Foundation Trust, Complex Trauma and Resilience Research Unit<br>Carter, Lesley-Anne; The University of Manchester, Centre for Biostatistics, Division of Population Health, Health Services Research and Primary Care<br>Kirk, Sue; The University of Manchester, School of Health Sciences<br>Petrussa, Cinzia; Greater Manchester Mental Health NHS Foundation Trust, Complex Trauma and Resilience Research Unit<br>Malpass, Fiona; Voice Collective, Mind in Camden<br>Shields, Gemma; University of Manchester, Centre for Health Economics<br>Thompson, Alexander; The University of Manchester, Manchester Centre for Health Economics<br>Morrision, Tony; The University of Manchester, Division of Psychology and Mental Health; Greater Manchester Mental Health NHS Foundation Trust, Psychosis Research Unit<br>Varese, Filippo; The University of Manchester, Division of Psychology and Mental Health; Greater Manchester Mental Health NHS Foundation Trust, Complex Trauma and Resilience Research Unit
**Primary Subject Heading**:	Mental health
Secondary Subject Heading:	Evidence based practice, Health services research, Paediatrics, Research methods
Keywords:	MENTAL HEALTH, Child & adolescent psychiatry < PSYCHIATRY, Psychosocial Intervention, Clinical Protocols

The ChUSE Trial - Improving Access to Psychological Therapies for Children with Distressing Sensory Experiences in NHS CAMHS: A Randomised Controlled Feasibility Trial Protocol

For peer review only

Authors:

1. Dr Sarah Parry, University of Manchester, Division of Psychology and Mental Health and Pennine Care NHS Foundation Trust, Young People's Mental Health Research Centre, , 225 Old St, Ashton-under-Lyne OL6 7SR, UK Email: sarah.parry@manchester.ac.uk
2. Dr Sarah Carr, Greater Manchester Mental Health NHS Foundation Trust, Complex Trauma and Resilience Research Unit, Manchester, UK
3. Dr Lesley-Anne Carter, University of Manchester, Centre for Biostatistics, Division of Population Health, Health Service Research and Primary Care, Manchester, UK
4. Professor Sue Kirk, University of Manchester, School of Health Sciences, Manchester, UK
5. Cinzia Petrussa, Greater Manchester Mental Health NHS Foundation Trust, Complex Trauma and Resilience Research Unit, Manchester, UK
6. Fiona Malpass, Voice Collective, Mind in Camden, London, UK
7. Gemma Shields, University of Manchester, Centre for Health Economics, Manchester, UK
8. Dr Alexander Thompson, University of Manchester, Centre for Health Economics, Manchester, UK
9. Professor Tony Morrison, University of Manchester, Division of Psychology and Mental Health and Greater Manchester Mental Health NHS Foundation Trust, Psychosis Research Unit, Manchester, UK
10. Professor Filippo Varese, University of Manchester, Division of Psychology and Mental Health and Greater Manchester Mental Health NHS Foundation Trust, Complex Trauma and Resilience Research Unit, Manchester, UK

Corresponding Author: Dr Sarah Parry, University of Manchester and Young People's Mental Health Research Centre, Pennine Care NHS Foundation Trust. University of Manchester | Division of Psychology and Mental Health | School of Health Sciences | Faculty of Biology, Medicine and Health, M13 9PL, UK Email: sarah.parry@manchester.ac.uk

Abstract

Introduction

Distressing sensory experiences (DSEs) are commonly reported by children and young people accessing Child and Adolescent Mental Health Services (CAMHS) in the UK. Despite their prevalence, evidence-based, tailored interventions for this population are scarce. The ChUSE trial aims to address this gap by evaluating the feasibility and acceptability of a novel brief talking therapy for children aged 8-16 years old and an accompanying parent coaching programme.

Methods and Analysis

A single-blind, two-arm randomised controlled feasibility trial will be conducted across three NHS Trusts in Greater Manchester, UK. Sixty young people under the care of CAMHS experiencing DSEs and their parents/caregivers will be randomised 1:1 to receive either Treatment as Usual (TAU) or TAU plus the ChUSE intervention. The intervention involves four child therapy sessions and three optional parent sessions. Primary feasibility outcomes include recruitment and retention rates, treatment adherence, and intervention safety. Secondary objectives assess the feasibility of data collection procedures and gather qualitative insights into trial acceptability and the influence of the intervention upon young people's wellbeing.

Ethics and Dissemination

The ChUSE Trial has received ethical approval through the Health Research Authority following review by a Research Ethics Committee specialising in paediatric research (ID 327343). The feasibility trial addresses a significant clinical gap in CAMHS provision and will also capture information as to current TAU practices, contributing novel data to guide future service provision, as TAU is currently understood to be heterogeneous. The information gathered through this trial will inform the design of a definitive trial evaluating the clinical effectiveness and cost-consequences of an early, age-appropriate intervention for DSEs. Findings and translational outputs will be shared through scientific and public forums to reach a variety of audiences.

Trial registration: ISRCTN12245618

Keywords: distressing sensory experiences, CAMHS, child mental health, cognitive therapy, compassionate mind training, early intervention, feasibility trial

Strengths and limitations of this study

1. Rigorous methodological design with clearly defined procedures that support transparency and replicability.
2. Systematic recruitment and sampling approach to minimise selection bias and enhance the credibility of the findings.
3. Use of validated data-collection tools, increasing reliability and consistency of measurement, paired with more novel and specialised measures to explore any phenomenological change.
4. Potential limitations in generalisability, as the sample is suitably small, so is unlikely to be representative of all relevant populations or contexts.
5. Risk of unmeasured confounding variables, which may influence interpretations despite efforts to control methodological bias.

For peer review only

Word count: 7,372 (not including abstract, references or checklists)

Improving Access to Psychological Therapies for Children with Distressing Sensory Experiences in NHS CAMHS: The ChUSE Trial Protocol

Introduction

Approximately half of children and young people attending Child and Adolescent Mental Health Services (CAMHS) report distressing sensory experiences (DSEs; [1]), including auditory and visual hallucinations, feelings of disconnection, and intrusive sensory thoughts [2,3]. Young people can experience DSEs due to the chronic stress of poverty[4] 4], bereavement, abuse and neglect [5][6], bullying [7], medical conditions such as epilepsy [8], adverse responses to medication [9], or disrupted sleep[10]. Distressing voice content has been associated with negative self-schemas and insecure attachments with caregivers [10]. Voice hearing is often transient in childhood and not usually a sign of severe mental illness, with DSEs (also referred to as multimodal hallucinations) typically less complex and less distressing in non-clinical samples [11, 12]. However, young people who experience even transient voice-hearing can report reduced quality of life at later follow-ups compared to their peers [13] and a higher rate of suicidal ideation [14], highlighting the potential long-term impact of voice hearing. Furthermore, DSEs in adolescence have been connected to later experiences of depression and anxiety [15], indicating a need for intervention as soon as possible to promote coping, wellbeing, and to prevent further difficulties.

A large-scale review [16] and primary research [17][18] indicate that many children begin to report unusual sensory experiences between the ages of 8 and 11 years, with 51% of a community sample describing these experiences as distressing [19]. From the age of 14, some young people may access specialist support through psychosis care pathways in the UK's National Health Service (NHS). However, referrals to such services can generate additional anxieties, potentially increasing self-stigma and adversely affecting wellbeing [20]. Current referral pathways for psychosis are not appropriate for many children experiencing DSEs, as only 15–22% of young people identified as at-risk for developing psychosis will access these pathways within a year [21]. This misalignment in referral pathways can increase anxiety for families and delay access to proportionate support in age-appropriate child and adolescent settings.

Providing earlier, non-psychosis-specific support within CAMHS could improve timely access to appropriate, proportionate interventions, while reducing future reliance on specialist services. Early intervention is crucial to promote coping strategies [22], enhance wellbeing [15], and to potentially mitigate the development of more severe conditions such as psychosis or mood disorders throughout the turbulent teenage years and into early adulthood.

Auditory verbal hallucinations are thought to be the most common multisensory experience, although this assumption is largely based on data with adults and young people aged 16 years and over. Voices can be comforting or distressing, and young people can experience both comforting and distressing voices at the same time [23], each sometimes taking on distinct identities[16,24,25]. Visual and tactile hallucinations can occur separately or concurrently and have been linked to trauma and/or anxiety [26, 27]. The impact of these multisensory experiences varies, influenced by

For peer review only

individual psychosocial factors, personal resources, and social support [28, 29]. Despite nearly 50% of children in CAMHS reporting multisensory experiences [1], there is currently no evidence-based talking therapy available for children with DSEs.

Young people have described benefitting from re-appraisal, normalizing [30], psychoeducation, externalisation [1], multisensory and cognitive restructuring coping strategies [17,19], similar to many of the approaches within cognitive therapies and compassionate mind training (CMT). Emerging findings suggest Compassion for Voices is a beneficial approach for young adult voice hearers, [31-33]. Our recent reviews have explored what is particularly important for young people with DSEs and first episode psychosis in therapeutic relationships [34], specific gender needs for girls when help-seeking [35], family members experiences of help-seeking for DSEs [36], and compassionate approaches to coping with voice hearing for young people [37]. These reviews recommend enhancing early support and communication about DSEs for young people, highlighting that girls and young women may experience more dismissive responses to their difficulties when they present to services, and the importance of collaborative therapeutic relationships and compassion-informed approaches for coping with hearing voices.

Drawing from the adult literature, a recent feasibility study on e-CBT for adults with DSEs found 4-6 sessions were acceptable for service users and progression criteria were met for recruitment, retention, and adherence to treatment [38]. Whilst it is positive to see the development of new interventions amongst the adult population [39], there remains a need for DSE research to be undertaken with young people in the unique setting of CAMHS, employing age-appropriate interventions [16]. Suitably tailored youth-focused interventions are limited in their availability and scientific evidence-base due to a scarcity of research with young people and their families to inform tailored protocols. Overall, research has overlooked younger children with DSEs which means important developmental and contextual information is currently missing from our understanding of how children experience DSEs and what support they and their families want from services.

Thus far, we know further work is needed with younger children to improve treatment pathways and reduce delays in accessing suitable support, and that clinicians desire clearer clinical guidance for tailored care [16]. Priorities for tailored care seem to include careful, developmentally informed assessment and caution around the use of structured interviews, which may insufficiently capture the form, function and content of DSEs[40]. Detailed clinical assessment is also important to distinguish between DSEs and hallucinations caused by neurological conditions such as migraine and occipital epilepsy [41]. Due to the role traumatic events [40]42]), substance use, sleep disorders, depression and anxiety may play in the experience of DSEs [42], a trauma-informed assessment may also be helpful where appropriate support can then be offered. Additionally, DSEs have been associated with sensory processing difficulties in paediatric samples for many years, although causal relationships and treatment implications remain unclear [43]. The ChUSE Trial is open to children in core CAMHS, autistic children, and children currently being assessed for neurodiversity in CAMHS. Adopting a neuroinclusive approach as early as possible in the treatment development process helps to ensure the final intervention is more acceptable and useful for all.

Developmental processes, including executive functioning, social cognition and language development, are thought to influence DSE phenomenology [44], although further research with younger children is needed to understand how to inform psychological support for DSEs. Overall, further research is needed to design developmentally sensitive assessments, interventions, treatment strategies, and to improve psychosocial outcomes [45]. Drawing on this emerging literature base and alongside lived experience consultations between 2016 and 2025, the ChUSE Trial aims to address recommendations for care in CAMHS by offering young people and their

For peer review only

parents/caregivers an intervention that mobilises existent knowledge to offer a tailored brief coping focused therapy, whilst assessing feasibility parameters to prepare for a definitive trial.

When supporting younger children with DSEs, it is essential to consider the role and involvement of parents and caregivers. Parental exclusion within services, and feelings of shame and guilt, can contribute towards relational instability within families [46]. Family interventions for psychosis (FIfP) have been found to improve cognitive functioning, reduce symptoms of psychosis, and reduce the likelihood of relapse [47]. Importantly, carer burden and wellbeing can also improve following a FIfP approach, indicating benefits for the wider family system of parental support, which may be especially relevant for younger children. The parent coaching sessions within the ChUSE Trial will offer a new and tailored approach for parents, offering separate spaces with a tailored therapeutic focus for parent and child.

In a recent review of parent's experiences of seeking help for young people with DSEs, we found many parents cannot find suitable care or are deterred from help-seeking following finding 'nothing on the other side of the door' of children's mental health services for DSEs, which can delay help-finding until the young person is in crisis [36]. Our primary research with parents and caregivers prior to the ChUSE Trial demonstrated parents want reliable information, skills and strategies to help their child cope [23]. Prior research identified that many families have dissatisfying experiences of help-seeking for hearing voices and would like more opportunities for normalising and holistic care [48]. Therefore, the ChUSE Trial meets an identified need in CAMHS, for children, practitioners and parents alike, offering a symptom-focused, low-intensity, systemic early-intervention, adding significantly to the clinical support for children and their parents and caregivers.

The ChUSE Trial

A significant challenge in treating DSEs for young people is the absence of child-centred research informing clinical guidelines, despite the recognised prevalence of these difficulties within CAMHS [1]. CAMHS practitioners frequently report missing the clinical guidance necessary to support this group of young people, resulting in restricted treatment options and delays in care for young people presenting with DSEs. To address this, the ChUSE trial will implement and evaluate an intervention protocol designed to offer early intervention for use within CAMHS. In time, this could provide practitioners with clear, practical guidance and ensure timely, appropriate support for young people. Informed by evidence from CBT interventions for adults and the few studies available that have involved children and young people, the ChUSE therapy integrates psychoeducation and therapeutic mechanisms from CBT and CMT to formulate young people's experiences of DSEs and identify potential coping strategies to reduce distress and enhancing coping for each young person.

A challenge for the ChUSE Trial is our limited understanding of what constitutes 'treatment as usual' (TAU) for this population. This adds complexity to the design and delivery of high-quality research in this area. To mitigate this, the ChUSE trial will incorporate a TAU arm, systematically gathering data on existing care practices across three NHS Trusts. This will provide essential information to inform the design of a future definitive trial.

For peer review only

Methods and Analysis

Aims and Objectives

Aim

To evaluate the feasibility and acceptability of conducting a future, larger-scale, definitive trial to assess the clinical effectiveness and cost-consequences of a brief psychological intervention for young people aged 8–16 years experiencing DSEs, accompanied by up to three parent coaching sessions.

Objectives

The primary objective is to assess key feasibility outcomes and gather data to inform the design of a future multi-site randomised controlled trial (RCT). Specifically, the study will examine:

- The feasibility of recruiting children and their parents or carers to the trial.
- The retention of participants throughout the trial period.
- Engagement with and adherence to the intervention and trial procedures.
- The safety of the intervention and trial procedures.
- Estimation of key parameters to support a sample size calculation for a future definitive efficacy trial.

Secondary Objectives

In addition to the primary feasibility outcomes, this study will address the following feasibility uncertainties:

- To determine the uptake of the optional parent coaching sessions.
- To evaluate the feasibility of collecting a set of proposed outcome measures, informing the selection of appropriate measures and data collection procedures for a future trial.
- To gather qualitative data exploring the acceptability of the intervention and trial procedures from the perspectives of young people, parents and carers, as well as their recommendations for refining the trial design.
- To collect qualitative insights from Child and Adolescent Mental Health Services (CAMHS) practitioners and clinical leads regarding barriers, facilitators, costs and consequences of implementing the intervention within routine CAMHS provision.

Primary Feasibility Outcomes

The main feasibility outcomes for this study are as follows:

- Recruitment rates, measured as the number of family units recruited and randomised per month.
- Participant retention rates, expressed as the proportion of participants completing outcome assessments at each time point.
- Treatment engagement and adherence, assessed by the number of sessions attended by young people and parents or carers allocated to both the ChUSE intervention plus treatment as usual (TAU) arm, and the TAU-only arm.

For peer review only

- Treatment fidelity, determined by the extent to which trial therapists deliver the ChUSE intervention according to the intervention protocol, as measured by fidelity checklists.
- Safety of the intervention, evaluated through the monitoring and recording of adverse events (AEs) and serious adverse events (SAEs), both related and unrelated to the intervention or trial procedures, using detailed AE reporting forms.
- Preliminary signals of efficacy and estimation of parameters required for a future sample size calculation, with outcome data collected at baseline (T0), three months (T1), and six months (T2).

Secondary Feasibility Outcomes
Secondary feasibility outcomes will include:

- Uptake of the parent coaching sessions, assessed through treatment engagement data, specifically the number of parents or carers who accept the offer of the PCS and the number of sessions attended.
- Feasibility of outcome data collection, determined by examining the completeness of the proposed outcome measures (percentage of missing responses and reasons for missing data), to guide measure selection and optimise data collection procedures for the future definitive trial.

Trial Design
This study will take the form of a feasibility randomised controlled trial (RCT) with an embedded process evaluation. It will employ a single-blind, parallel-group design, comprising two arms: (1) a brief psychological intervention involving four-six sessions of individual talking therapy for DSEs, with the optional offer of up to three sessions for parents/caregivers, in addition to treatment as usual (TAU); and (2) TAU alone. Participants in the intervention arm will also be offered a joint session zero with the trial therapist to discuss taking part in the intervention and to plan appointments. Outcome assessments will be conducted with all participants (N=60 parent-child dyads) at baseline (T0), at the end of the intervention (three months post-randomisation, T1), and at six months post-randomisation (T2). At T2, participants will be invited to take part in qualitative interviews to inform the process evaluation. Participants who do not wish to take part in qualitative interviews will be sent a brief online questionnaire via Qualtrics to enable participants to provide feedback on their experience of being in the trial and their experience of the intervention or CAMHS, depending on which arm of the trial they were randomised to. The trial is due to run between October 2024 and November 2026.

While the primary focus of the study is on feasibility outcomes, the process evaluation will generate both quantitative and qualitative data to enhance understanding of potential mechanisms of change, the acceptability of the intervention, preliminary indications of clinical promise within CAMHS settings as well as likely cost drivers and wider system consequences. Quantitative measures will help to track changes in symptoms and wellbeing over time, while qualitative feedback will capture participants' experiences of the intervention, trial procedures, and how they have experienced their engagement in the trial. Qualitative interviews with practitioner (N=20) will also be conducted to inform interpretation of contextual factors and future implementation considerations.

For peer review only

Trial Setting

The trial will be conducted across multiple sites, with participant identification, recruitment, and treatment delivery taking place in outpatient CAMHS across three NHS Trusts in Greater Manchester. Should additional recruitment be required, participant identification may be extended to other comparable services within Trusts supported by the NIHR Research Delivery Network.

Patient and Public Involvement and Engagement (PPIE) and Equality, Diversity, Inclusion and Accessibility (EDIA)

This study has been co-developed with meaningful input from young people, parents and caregivers, and professionals with personal lived and clinical experience. A Lived Experience Advisory Group (LEAG), consisting of eight young people, will advise on the study throughout its duration. The LEAG has contributed to the design of study materials, intervention content and recruitment approaches through their involvement with Voice Collective, a lived experience led community group, to ensure relevance, accessibility, and sensitivity to lived experience. Alongside the LEAG, a Parent and Caregiver Advisory Group comprising six members provides insights into family needs, language appropriateness, and acceptability of procedures from a caregiver's perspective.

A Stakeholder Advisory Group of ten professionals, including CAMHS clinicians, education specialists, and voluntary sector representatives, will offer strategic guidance on implementation, dissemination, and real-world relevance. Governance oversight will be provided by a Trial Steering Committee (TSC), which includes eight members with expertise in child mental health, trial methodology, and public involvement.

We are committed to upholding the principles of equality, diversity, inclusion, and accessibility (EDIA) across all components of the trial. Recruitment materials and procedures have been co-produced to ensure they are inclusive and appropriate for a range of cultural, linguistic, and neurodevelopmental needs. Monitoring of demographic data will help ensure transparency and accountability in terms of equitable access and identify any disparities in participation or outcomes.

Participants and Recruitment

This feasibility trial has been designed to be inclusive and accessible, with eligibility criteria and recruitment procedures shaped by extensive public and patient involvement and engagement (PPIE) and consultation with clinical stakeholders. Recognising that DSEs are not condition-specific, young people will not be excluded based on mental health diagnoses, co-occurring treatments, or neurodevelopmental status. The trial and intervention have been specifically designed to be inclusive of neurodiverse participants, and parents/caregivers are invited, but not required, to take part in the parent/caregiver sessions.

Eligibility Criteria

Children and young people (1) aged 8–16 years who are (2) currently receiving support from participating CAMHS and who (3) self-identify as experiencing DSEs will be eligible to participate. Given the absence of validated clinical thresholds for DSEs, eligibility will be confirmed using the Manchester Voices Inventory for Children (MAVIC) and the unusual sensory experiences questionnaire (MUSEQ) in screening to determine the presence of at least one self-identified current DSE, following initial referral. Although additional funding is not available across all locations, translators and interpreters will be sourced where funding from clinical services is available. If funding for this provision is not available, (4) the young person and their parent/caregiver will need

For peer review only - http://bmjopen.bmj.com/site/about/guidelines.xhtml

to have conversational English to take part. (5) Current inpatient admission for acute mental health crisis, or (6) participation in another active psychological intervention trial, will also preclude people from taking part.

Participant Identification and Recruitment

Participants will be identified from existing CAMHS referrals where young people have self-reported DSEs. Research staff will regularly attend multidisciplinary team meetings to support clinicians in identifying eligible participants. Clinicians will provide initial study information to families and seek verbal consent for contact by the research team. The research team will subsequently contact interested families to provide full study details and obtain informed consent from parents/caregivers and young people aged 16 years and over, and assent from young people aged 15 years and under, documented in accordance with local governance procedures.

Screening and Enrolment

Following consent and assent, screening assessments using the MAVIC and MUSEQ measures will confirm eligibility prior to enrolment. Consenting participants will complete baseline assessments (T0) before randomisation. Young people and parents/caregivers will be offered choices regarding the format and location of assessments (in-person, telephone, or video call) and whether to complete these independently or together.

Sampling and Recruitment Targets

As a feasibility trial, a formal power calculation is not required [49, 50]. In line with recommendations for pilot trials, we aim to randomise 60 young people and parent/caregiver dyads [51, 52]. This sample size is considered sufficient to estimate outcome measure variability and inform the design of a future definitive trial. Recruitment will take place over 19 months, with a minimum target of four participants per month in the first year. A recruitment review will be conducted at month 12, with contingency plans to extend to additional NHS Trusts if 75% of the target sample has not been achieved 6 months before the end of the trial.

Consent, Assent, and Withdrawal

Informed consent and assent will be obtained prior to participation in each stage of the study (T0, T1, T2). Participants may provide consent via signed hard copy, electronic form, or audio-recorded verbal consent, depending on preference. The consent process will be clearly documented, and participants will be reminded that participation is voluntary and can be withdrawn at any time without affecting their access to usual clinical care. If a participant withdraws but then wishes to opt back in, they may do so, if this is within four weeks of their six-month post-randomisation date for T2 data collection. Additional optional consent provisions will cover audio-recording assessments, use of anonymised data for secondary analysis, future research contact, teaching, and sharing study results. Based on media interest in our work in this area previously, there is also an option for participants to opt-in to receive information about future media enquiries or invitations. In line with the SPIRIT statement [53], predefined criteria for the discontinuation or interruption of the intervention will be applied at the individual participant level, with intervention delivery paused or possibly stopped where a serious adverse event (SAE) is assessed as being related to the ChUSE intervention procedures.

Remuneration and Expenses

Participants will receive a £10 shopping voucher as a thank you for completing outcome assessments at T0, T1 and T2. Reasonable travel expenses can be reimbursed upon request for in-person research

For peer review only

appointments, although the majority of trial procedures will be conducted remotely or during routine clinic visits.

Measures

Eligibility Screening and Baseline Assessments

Eligibility will be confirmed via a short screening form (age and presence of at least one self-identified current distressing sensory experience using the MAVIC and MUSEQ). Those eligible will proceed to baseline (T0) assessments, including demographic, clinical history, and validated outcome measures. Ineligible participants will be debriefed, receive a voucher, and be offered a summary of their screening results to share with their clinical team.

Assessments will be flexible, delivered face-to-face or remotely, with options for breaks or multiple shorter sessions. After completing the baseline assessment, the young person (and, by proxy, their parent/caregiver) will be randomised to receive either TAU or ChUSE+TAU. Young people's preferences around who is present during assessment will be respected. Measures will be repeated at T1 and T2, as shown in table one and three.

Table 1: Assessments for children and young people

T0 (baseline)	T1 (3-month follow-up)	T2 (6-month follow-up)
Screen: MAVIC	MAVIC	MAVIC
Screen: Unusual Sensory Experiences Questionnaire	-	-
Demographics	-	-
Strengths and Difficulties Questionnaire (SDQ)	SDQ	SDQ
Kidcope Scale	Kidcope Scale	Kidcope Scale
The Stirling Children's Wellbeing Scale	The Stirling Children's Wellbeing Scale	The Stirling Children's Wellbeing Scale
Self-Compassion Scale Short Form (SCS-SF)	SCS-SF	SCS-SF
EQ-5D-Y	EQ-5D-Y	EQ-5D-Y
CHU-9D	CHU-9D	CHU-9D
CSS	CSS	CSS
		TAU checklist

Table 2: Measures and Completion Times

Instrument used in the proposed trial	Number of items	Indicative completion time
Strengths and Difficulties Questionnaire Self-Report version for 11–17-year-olds [54]	25	10-15 minutes
Manchester Voices Inventory for Children (MAVIC; [23])	18	10-12 minutes

For peer review only

Unusual Experiences Questionnaire (MUSEQ;[55])	9 (only issued at T0)	10-15 minutes
Stirling Children's Wellbeing Scale [56]	12	8-10 minutes
Brief Self-compassion Scale [57]	12	8-10 minutes
KidCOPE scale [58]	15	10-20 minutes
EQ-5D-Y [59]	5	5 minutes
Child Health Utility Instrument (CHU-9D; [60])	9	5-8 minutes
Child Stigma Scale (CSS) – adapted multisensory experiences [61]	7	5 minutes
Treatment as usual checklist (TAU checklist)	12	8-10 minutes

Table 3: Parent/carergiver measures

T0 (baseline)	T1 (3-month follow-up)	T2 (6-month follow-up)
Demographics	-	-
SDQ (parent version)	SDQ (parent version)	SDQ (parent version)
Adult Wellbeing Scale	Adult Wellbeing Scale	Adult Wellbeing Scale
Parental Stress Scale	Parental Stress Scale	Parental Stress Scale
Coping Health Inventory for Parents	Coping Health Inventory for Parents	Coping Health Inventory for Parents
EQ-5D	EQ-5D	EQ-5D

Participants do not have to complete all questions in the outcome measures. At the end of baseline assessment, participants will be informed of their trial arm allocation by a member of the research team.

Randomisation and Blinding

Participants will be randomly allocated on a 1:1 basis to either (1) ChUSE talking therapy plus parent sessions alongside TAU, or (2) TAU alone. Randomisation will be conducted independently using a web-based service (sealedenvelope.com), with concealed allocation via a pseudo-random sequence employing random permuted blocks of varying sizes, unknown to the study team.

Following baseline assessment, research workers will notify the Chief Investigator (CI) or a delegate about participant eligibility for randomisation. The CI or trial therapist (or delegate) will then enter the participant ID into the secure online system, which will immediately generate the allocation. Allocation outcomes will be communicated only to unblinded team members (CI, Co-CI, and the trial therapist) and centrally logged on the secure platform and a restricted-access randomisation log.

For peer review only

Blinding will be maintained for research workers and trainees responsible for data collection. Research workers and trainees will not access clinical notes or treatment information post-randomisation and will be routinely reminded, along with participants and clinical teams, to avoid disclosing treatment allocations during assessments. The CI, therapist, and co-investigators will remain unblinded in line with standard practice for psychological intervention trials. Any breaches of blinding will be documented, and where possible, follow-up will be reassigned to a blinded researcher.

Interventions

Comparator: Treatment as Usual (TAU)

Participants allocated to the TAU arm will continue to receive care as usual in CAMHS. The trial team will not intervene in routine care or request clinicians to withhold any referrals or treatments typically offered to participants. This aligns with established practices for pragmatic psychological intervention trials [62]. In the absence of national clinical guidelines specifically addressing DSEs for children, clinicians will be asked to describe what interventions and supports they provide to these young people and their families. These data will be systematically collected and tabulated using a structured checklist and free-text responses, offering novel insights into routine clinical practices for DSE [63].

ChUSE Intervention

The feasibility trial will deliver the ChUSE intervention through a qualified, NHS-employed, HCPC-registered Clinical Psychologists, trained and supervised in the protocol by the study's Chief Investigator (CI). The manualised intervention will comprise of ideally seven 50-minute sessions (four child-focused and up to three parent coaching sessions) delivered over a three-month period. While the intervention will always remain brief, there is some flexibility in the number of sessions offered to young people to support engagement for up to a maximum of six sessions. This is to allow for pacing of content and accommodate breaks or shorter sessions as required by families with different needs. There will also be a session zero offered to all participants in the trial arm to attend as a child-parent/caregiver dyad. After each session, the therapist will complete an adherence checklist to monitor progression towards therapy milestones, the use of intervention strategies in line with the manual, and any deviations [64].

Parents/caregivers of children in the intervention arm will be offered the parent sessions alongside the child-focused therapy. Importantly, parental engagement in the parent sessions will not be a prerequisite for children to access individual therapy. The structure and delivery format of both child and parent components were informed by PPIE consultations, which recommended offering a choice of in-person or online sessions via Microsoft Teams to enhance accessibility.

ChUSE Child-Focused Intervention

The child-focused therapy is a psychological intervention targeting DSEs, delivered across four-six structured sessions. Content draws upon contemporary research on child distress, unusual sensory experiences, and psychosis-like phenomena.

- The first session will focus on exploring the young person's DSEs and goals, psychoeducation and normalisation [1, 30, 65].

For peer review only

- The second will introduce formulation and multisensory coping strategies based on compassion-focused therapy (CFT) principles, shaped by recommendations from young people with lived experience and the growing compassion for voices literature [17, 23, 32].
- The third session will include cognitive appraisal work, coping skills practice, and simple self-formulation exercises [31, 33].
- The final session will focus on compassion-focused reflection, consolidation of coping strategies, and safety/future planning.

ChUSE Parent Training Programme

Parents/caregivers will be invited to attend three structured sessions designed to complement the child's therapy.

- The first session offers psychoeducation, normalisation, reappraisal strategies, and therapist-guided, scenario-based problem-solving [19, 65].
- The second introduces compassion-focused coping techniques and self-kind containment strategies.
- The third provides therapist-guided reflective discussions, consolidating new knowledge and coping skills.

Between-session homework will involve reading psychoeducational materials and practising coping strategies, facilitating skills generalisation and enhancing therapeutic outcomes [66].

Fidelity and Process Monitoring

With participant consent, therapy sessions may be audio- or video-recorded for supervision and fidelity monitoring purposes. Declining to consent for recording will not affect the care received. Recordings will be destroyed following review or fidelity assessment.

Fidelity will be monitored via a therapist-completed intervention integrity checklist developed for the study. Additionally, a random sample of 10% of recorded sessions will be rated using the Cognitive Behaviour Therapy Scale for Children and Young People (CBT-SCYP) to assess adherence to the ChUSE manual [67]. The therapeutic alliance will also be measured post-intervention using the Therapeutic Alliance Scale for Children and Caregivers (TASC), completed by children, and parents/caregivers [68].

Follow-Up Assessments and Safeguarding Procedures

Participants will be invited to complete follow-up assessments at three months (T1) and six months (T2) post-randomisation. These assessments will replicate the baseline battery of measures, as shown in Tables One and Three. To enhance active safety monitoring, adverse events (AEs) will be systematically assessed during each contact point and through consultation with clinical notes.

Each assessment is expected to last approximately one hour, with options for breaks or multiple shorter sessions to accommodate participant's needs (see Table 2). T2 may take up to two hours, as this data point includes qualitative interviews. For all participants, there is then an option to provide further qualitative feedback through a Qualtrics survey, based on the same questions as the semi-structured interview guides. There is one survey for children and young people, and one for parents/caregivers. Upon completion of the final follow-up, participants will be formally debriefed

For peer review only

about the feasibility RCT and, where consent has been provided, reminded about possible future contact for research updates or follow-up studies.

To minimise missing data, the research team will make multiple attempts to contact participants before considering them lost to follow-up. Data collection methods will include in-person, telephone, video conferencing, or postal options. Any spurious or incomplete data will be reviewed by the research team, who will determine appropriate actions such as verification or re-collection.

Safeguarding procedures will adhere to local and national guidelines. The Chief Investigator (CI) will be informed of any safeguarding concerns, and referrals will be made to the appropriate safeguarding team for the child's local area. In cases of immediate risk, the Emergency Duty Team will be contacted directly. Contact details for safeguarding services across Greater Manchester's ten boroughs will be held by designated trial staff, and procedures will follow the Greater Manchester Mental Health NHS Foundation Trust safeguarding policies.

Progression Criteria

Progression to a definitive trial will be determined using pre-specified feasibility criteria across five domains (see Table 4). Recruitment feasibility will be assessed by the number of parent–child dyads consented and randomised per month, alongside referral sources and reasons for non-eligibility or non-participation. Feasibility will be demonstrated if an average of at least three dyads are recruited per month. Recruitment of two dyads per month will indicate conditional feasibility, requiring the identification of additional recruitment strategies, while recruitment averaging one participant per month (fewer than 20 participants overall) will indicate that progression is not feasible within the current design. However, these criteria will be applied with some flexibility where there are contextual factors to consider, such as the impact of school holidays and national holidays on family's engagement with services.

Intervention feasibility will be evaluated through adherence and attendance. Progression will be supported if at least 70% of participants allocated to the intervention arm complete the full course of treatment, defined as engagement with all four CYP sessions. Completion rates of 50–70% will indicate conditional feasibility, contingent on identifying barriers and potential adaptations informed by qualitative interviews and adherence data. Completion rates below 50% will indicate that progression to a definitive trial is not feasible without substantial modification of the intervention or delivery model.

Trial retention will be assessed by the proportion of participants completing end-of-treatment and follow-up assessments. Retention of at least 70% of participants will demonstrate feasibility. Retention rates between 30% and 70% will indicate conditional feasibility, requiring the development of strategies to address attrition, informed by reasons for withdrawal, recorded at the point of withdrawal in a participant tracker, and qualitative interview data. Retention below 30% will indicate non-feasibility of progression under the current trial design.

The feasibility of including the parent training programme in a future definitive trial will be determined by parental uptake and attendance. Inclusion of the parent sessions will be considered feasible if more than 70% of parents allocated to the intervention arm take up the offer. Uptake of 50–70% will indicate conditional feasibility, dependent on evidence of session attendance, potential added benefit, and health economic data suggesting cost-effectiveness. Uptake below 50% will indicate that inclusion of the parent sessions is not feasible within the current design.

Safety and acceptability of trial procedures will be assessed through monitoring of serious adverse events (SAEs). Feasibility will be demonstrated if fewer than 5% of participants experience SAEs

For peer review only - http://bmjopen.bmj.com/site/about/guidelines.xhtml

related to trial procedures. SAE rates between 5% and 10% will indicate conditional feasibility, requiring the identification of additional risk-mitigation strategies informed by qualitative data. SAE rates exceeding 10% will indicate that progression to a definitive trial is not feasible. All SAEs will be reviewed by the Trial Steering Committee Chair and reported to the sponsor as appropriate.

Table 4: *Progression Criteria*

**Domain**	**Green**	**Amber**	**Red**
Recruitment	≥3 dyads/month	2 dyads/month	≤1/month (<20 total)
Intervention completion	≥70% complete	50–70%	<50%
Trial retention	≥70% retained	30–70%	<30%
Parent programme uptake	>70% uptake	50–70%	<50%
Safety (SAEs)	<5% SAE	5–10%	>10% SAE

Analysis of Key Feasibility Outcomes

Descriptive statistics will be employed to summarise key feasibility and acceptability outcomes, including trial recruitment, retention, therapy engagement, and treatment fidelity. Participant flow through the trial will be reported in accordance with the CONSORT extension for feasibility and pilot trials [49], detailing the number of referrals received per month, recruitment sources, number of participants contacted and assessed for eligibility, numbers consented (with reasons for ineligibility or declining participation when available), randomisation to trial arms, adherence to allocated interventions, and retention at the three- and six-month follow-up assessments.

All quantitative data analysis will be conducted according to a pre-specified statistical analysis plan prepared by our trial statistician (co-author LAC) and available upon request from the corresponding author. Descriptive analyses of therapy session records and fidelity checklists will estimate the adherence of therapists to the intervention protocol. Adverse events (AEs) and serious adverse events (SAEs) will be tabulated by trial arm and reviewed to assess the safety and acceptability of the intervention and study procedures.

In addition, descriptive statistics (mean, standard deviation, minimum, maximum, range, and percentage of missing data) will be reported for all outcome measures at baseline and follow-up assessments, disaggregated by trial arm. These data will inform feasibility parameters for a future definitive trial, including estimates of variance and preliminary outcome differences. No hypothesis testing for clinical effectiveness (p-values) will be conducted, but where appropriate, mean differences, standard deviations, and 80% confidence intervals will be calculated at follow-up time points to provide a preliminary indication of the 'promise of efficacy' of the ChUSE intervention on valued trial outcomes [69].

To support the feasibility of future health economic evaluation, the completeness and quality of data collected using the EQ-5D [70], EQ-5D-Y [59], and CHU-9D [60] will be summarised. Intervention and

For peer review only

TAU service costs will be estimated using national unit cost data from NHS England and the Personal Social Services Research Unit.

Qualitative Evaluation

Interviews will be guided by topic guides co-developed with the PPIE groups and refined iteratively throughout the trial. Qualitative inquiry will be undertaken to explore participant and clinician experiences of trial processes and intervention delivery, which is essential for informing the development and future implementation of complex interventions [71]. Semi-structured interviews will be conducted by members of the research team at mutually convenient locations, either face-to-face, online via Microsoft Teams, or by telephone. Young people may be accompanied by a trusted adult if they wish.

Interviews will be audio-recorded, transcribed verbatim using secure transcription software, and subsequently reviewed and anonymised by a second member of the research team. Transcripts will be analysed using the Framework Method [72], a structured and transparent approach suitable for multidisciplinary health research teams [73]. Regular interpretation meetings will be held among the qualitative research team and LEAG to promote reflexivity and transparency during the analytic process.

Future access to anonymised final datasets will be considered case-by-case by the CI and Trial Sterring Committee, ensuring no risk of participant identification. Study findings will be disseminated through peer-reviewed publications, conference presentations, public forums, and participant summaries, in line with NIHR publication requirements.

Nested Validation Study: Manchester Voices Inventory for Children and Young People – Revised (MAVIC-R)

This nested validation study within the ChUSE Trial aims to assess the reliability, validity, and usability of the Manchester Voices Inventory for Children and Young People - Revised (MAVIC-R). MAVIC-R is a novel communication and assessment tool designed to help young people describe their unusual sensory experiences (USEs, which may be comforting, banal, or distressing) or DSEs in a non-pathologising, supportive manner. This study seeks to explore the tool's psychometric properties and explore its practical applications in NHS CAMHS and related services.

Research Questions

1. How effectively does MAVIC-R facilitate communication about DSEs with young people?
2. Which components are most helpful in exploring personal appraisals and meanings of DSEs?
3. How do young people, caregivers, and professionals perceive the tool's utility?
4. What are the practical and therapeutic implications of its use in CAMHS?

Research Design

Phase 1: MAVIC-R Refinement
Discussions with the ChUSE PPIE groups will inform updates to the existing MAVIC, expanding its

For peer review only

scope from voice-hearing to multisensory experiences. Insights from these consultations will guide revisions to language and content.

Phase 2: Validation Study
A mixed-methods design will assess the psychometric properties and practical acceptability of the MAVIC-R:

- **Quantitative**: An online survey with young people will assess item properties and factor structure via Exploratory Factor Analysis (EFA).
- **Qualitative**: Mental health professionals will trial the tool in practice and provide feedback through interviews on its implementation and acceptability.

Participant Recruitment

- Up to 150 Young People (Aged 8–18) with lived experience of USEs, recruited through professional and lived experience networks, such as Voice Collective.
- 10 Parents/Caregivers of young people with USEs
- 10 Multiservice Professionals working with young people experiencing USEs

Inclusion Criteria

- Young people aged 8–18, fluent in conversational English, with personal experiences of one or more USEs
- Caregivers with current or past experience of supporting a young person with USEs
- Professionals with regular experience working with young people experiencing USEs

Exclusion Criteria

- Young people outside the specified age range, or whose current mental health prevents participation
- Professionals/caregivers without relevant experience
- Autistic young people whose USEs are solely attributed to sensory processing differences

Recruitment Process
Participants will be recruited via the RRDN, NHS networks, peer organisations (e.g. Voice Collective), and professional contacts. The same referral process as the wider ChUSE Trial will be followed, with practitioners completing brief referral forms with consent.

Consent/Assent
Participants will provide electronic consent via Qualtrics for surveys or audio-recorded consent for in-person interviews. For those under 16, caregiver consent and participant assent will be obtained.

Data Collection and Analysis

Audio-recorded interviews and focus groups will be transcribed and analysed thematically [74] using NVivo. Qualitative survey data will also be analysed thematically, and quantitative survey data will be analysed in SPSS. Data will be screened for missingness, item correlations examined, and sampling adequacy assessed via KMO and Bartlett's tests. EFA using Maximum Likelihood extraction

For peer review only

with oblique rotation will determine factor structure, guided by scree plots, Kaiser's criterion, and parallel analysis. Reliability will be tested via Cronbach's alpha and item-total correlations.

Expected Outcomes and Dissemination

If validated, the MAVIC-R will provide a practical, non-stigmatising tool for facilitating discussions about USEs with young people, supporting tailored, person-centred care. Its use could foster more holistic, youth-informed care pathways within CAMHS and other youth services. Findings will be disseminated via peer-reviewed publications, stakeholder summaries, and multimedia resources for young people, families, and professionals. This study will also enable the inclusion of a validated, youth-specific measure in a future definitive ChUSE Trial.

Trial Endpoints, Ethical and Regulatory Considerations, and Data Management

The intervention period for this trial is scheduled to conclude by 1st March 2026, with all data collection for the trial completed by 1st June 2026. The nested validation study will be completed by the 1st December 2026. Prior to study commencement, the protocol, consent materials, and study documents will obtain favourable NHS REC and HRA approvals, with local Capacity and Capability agreements from participating NHS Trusts. The study will adhere to the Declaration of Helsinki, Good Clinical Practice, and the UK Policy Framework for Health and Social Care Research (2017).

Regulatory compliance will be maintained through appropriate approvals for all participating sites, timely submission of amendments, and regular oversight from the Sponsor and TSC. Protocol deviations will be formally logged and corrective actions agreed upon collaboratively.

Risk management procedures will include bespoke SOPs for safeguarding, clear limits of confidentiality, procedures for managing participant distress, and strict lone working and infection control policies for face-to-face contact.

Adverse events (AEs) and serious adverse events (SAEs) will be monitored closely, recorded according to established SOPs, and reviewed for severity, relatedness, expectedness, and seriousness. SAEs that are unexpected and related will be reported to the REC and Sponsor in line with standard HRA timelines.

Data management will comply with the Data Protection Act 2018 and GDPR. All personal data will be pseudonymised, securely stored, and deleted or anonymised at study end. Research data will be stored on encrypted servers, with access restricted to authorised study personnel. Final datasets and supporting materials will be archived securely with the Sponsor.

Ethics and Dissemination

The ChUSE Trial received ethical approval on 2nd May 2024 through the Health Research Authority following review by a Research Ethics Committee (REC) specialising in paediatric research (ID 327343). Any amendments to the protocol will be submitted to the REC committee as modifications. This study was registered with the ISRCTN on 14th June 2024 (https://www.isrctn.com/ISRCTN12245618).

For peer review only

Study findings will be widely disseminated through Open Access publications and presentations at national and international conferences. alongside more accessible multimedia disseminations developed in conjunction with our PPIE group such as podcasts, animations, infographics and youth friendly summaries. In parallel, findings will be disseminated through policy briefings and stakeholder events to inform national guidance and service development, with the aim of influencing policy and practice in child and adolescent mental health care. If successful, this study has the potential to inform clinical guidelines, contribute to workforce development through training and supervision models, and ultimately improve the quality and safety of services offered to young people in NHS mental health settings.

Patient and Public Involvement

Public engagement has been central to the development and design of this study, with young people, families, and practitioners contributing to intervention refinement, study materials, and data collection processes. Their input ensures that the research is sensitive to the needs and preferences of those it aims to support, and that dissemination plans are inclusive and accessible. We will endeavour to adopt the multisensory approach of the ChUSE intervention into the development of multisensory and multimedia translational outputs from the study, suitable and engaging for a range of audiences, in collaboration with our advisory groups.

Dissemination activities will be designed to engage a range of audiences, including young people, families, the public, and professionals across health, education, and voluntary sectors. A co-produced multimedia portfolio, including short animations, infographics, podcasts, and youth-friendly summaries, will support accessible communication of findings. These outputs will be developed in collaboration with the LEAG and shared through digital platforms, schools, CAMHS services, and third-sector networks.

Declarations: Ethics approval and consent to participate: Approved by NHS Research Ethics Committee (Ref: IRAS 327343). This study was funded by the National Institute for Health and Care Research (NIHR). The views expressed are those of the authors and not necessarily those of the NIHR or the Department of Health and Social Care.

Consent for publication: Not applicable.

Availability of data and materials: The datasets generated and/or analysed during this study will be available from the corresponding author on reasonable request.

Competing interests: The authors declare that they have no competing interests.

Funding: National Institute for Health and Care Research (NIHR) Research for Patient Benefit Programme (NIHR205326).

Author contributions: SP and FV conceived the study and developed the protocol. FM contributed to intervention development and PPI coordination. TM, SK, and DF provided methodological expertise. LAC advised on statistical analyses. GS and AT advised on health economics. All authors reviewed and approved the final manuscript.

For peer review only

Acknowledgements: The authors thank the young people, parents, CAMHS practitioners, and members of the Lived Experience Advisory Group who contributed to the development of this protocol.

For peer review only

References

[1] Jolley, S., Kuipers, E., Stewart, C., Browning, S., Bracegirdle, K., Basit, N., Gin, K., Hirsch, C., Corrigall, R., Banerjea, P., Turley, G., Stahl, D., & Laurens, K. R. (2018). The Coping with Unusual Experiences for Children Study (CUES): A pilot randomized controlled evaluation of the acceptability and potential clinical utility of a cognitive behavioural intervention package for young people aged 8–14 years with unusual experiences and emotional symptoms. *British Journal of Clinical Psychology*, *57*(3), 328–350. https://doi.org/10.1111/bjc.12176

[2] Johns, L. C., Kompus, K., Connell, M., Humpston, C., Lincoln, T. M., Longden, E., Preti, A., Alderson-Day, B., Badcock, J. C., Cella, M., Fernyhough, C., McCarthy-Jones, S., Peters, E., Raballo, A., Scott, J., Siddi, S., Sommer, I. E., & Larøi, F. (2014). Auditory Verbal Hallucinations in Persons With and Without a Need for Care. *Schizophrenia Bulletin*, *40*(Suppl_4), S255–S264. https://doi.org/10.1093/schbul/sbu005

[3] Maijer, K., Palmen, S. J. M. C., & Sommer, I. E. C. (2017). Children seeking help for auditory verbal hallucinations; who are they? *Schizophrenia Research*, *183*, 31–35. https://doi.org/10.1016/j.schres.2016.10.033

[4] Hastings, P. D., Serbin, L. A., Bukowski, W., Helm, J. L., Stack, D. M., Dickson, D. J., Ledingham, J. E., & Schwartzman, A. E. (2020). Predicting psychosis-spectrum diagnoses in adulthood from social behaviors and neighborhood contexts in childhood. *Development and Psychopathology*, *32*(2), 465–479. https://doi.org/10.1017/S095457941900021X

[5] Corstens, D., & Longden, E. (2013). The origins of voices: Links between life history and voice hearing in a survey of 100 cases. *Psychosis*, *5*(3), 270–285. https://doi.org/10.1080/17522439.2013.816337

For peer review only

[6] Longden, E., Madill, A., & Waterman, M. G. (2012). Dissociation, trauma, and the role of lived experience: Toward a new conceptualization of voice hearing. *Psychological Bulletin*, *138*(1), 28–76. https://doi.org/10.1037/a0025995

[7] Domínguez, T., Nieto, L., Fresán, A., Sheinbaum, T., & Robles, R. (2025). The association of bullying and self-esteem with psychotic-like experiences and clinical high risk for psychosis symptoms. *Child and Adolescent Psychiatry and Mental Health*, *19*(1), 51. https://doi.org/10.1186/s13034-025-00900-w

[8] Bisulli, F., Menghi, V., Vignatelli, L., Licchetta, L., Zenesini, C., Stipa, C., Morigi, F., Gizzi, M., Avoni, P., Provini, F., Mostacci, B., d'Orsi, G., Pippucci, T., Muccioli, L., & Tinuper, P. (2018). Epilepsy with auditory features: Long-term outcome and predictors of terminal remission. *Epilepsia*, *59*(4), 834–843. https://doi.org/10.1111/epi.14033

[9] Capaldi, V. F., & Carr, R. B. (2010). Citalopram-induced hallucinations and delusions in a young adult. *General Hospital Psychiatry*, *32*(6), 648.e1-648.e3. https://doi.org/10.1016/j.genhosppsych.2010.07.008

[10] Waters, F., Blom, J. D., Jardri, R., Hugdahl, K., & Sommer, I. E. C. (2018). Auditory hallucinations, not necessarily a hallmark of psychotic disorder. *Psychological Medicine*, *48*(4), 529–536. https://doi.org/10.1017/S0033291717002203

[11] Medjkane, F., Notredame, C.-E., Sharkey, L., D'Hondt, F., Vaiva, G., & Jardri, R. (2020). Association between childhood trauma and multimodal early-onset hallucinations. *The British Journal of Psychiatry*, *216*(3), 156–158. https://doi.org/10.1192/bjp.2019.266

[12] Skokauskas, N., Pillay, D., Moran, T., & Kahn, D. A. (2010). Transient Auditory Hallucinations in an Adolescent. *Journal of Psychiatric Practice®*, *16*(3), 187. https://doi.org/10.1097/01.pra.0000375716.77831.ca

For peer review only

[13] Calkins, M. E., Moore, T. M., Satterthwaite, T. D., Wolf, D. H., Turetsky, B. I., Roalf, D. R., Merikangas, K. R., Ruparel, K., Kohler, C. G., Gur, R. C., & Gur, R. E. (2017). Persistence of psychosis spectrum symptoms in the Philadelphia Neurodevelopmental Cohort: A prospective two-year follow-up. *World Psychiatry*, *16*(1), 62–76. https://doi.org/10.1002/wps.20386

[14] Fujita, J., Takahashi, Y., Nishida, A., Okumura, Y., Ando, S., Kawano, M., Toyohara, K., Sho, N., Minami, T., & Arai, T. (2015). Auditory verbal hallucinations increase the risk for suicide attempts in adolescents with suicidal ideation. *Schizophrenia Research*, *168*(1), 209–212. https://doi.org/10.1016/j.schres.2015.07.028

[15] Kelleher, I., & Cannon, M. (2011). Psychotic-like experiences in the general population: Characterizing a high-risk group for psychosis. *Psychological Medicine*, *41*(1), 1–6. https://doi.org/10.1017/S0033291710001005

[16] Maijer, K., Hayward, M., Fernyhough, C., Calkins, M. E., Debbané, M., Jardri, R., Kelleher, I., Raballo, A., Rammou, A., Scott, J. G., Shinn, A. K., Steenhuis, L. A., Wolf, D. H., & Bartels-Velthuis, A. A. (2019). Hallucinations in Children and Adolescents: An Updated Review and Practical Recommendations for Clinicians. *Schizophrenia Bulletin*, *45*(Supplement_1), S5–S23. https://doi.org/10.1093/schbul/sby119

[17] Parry, S., & Varese, F. (2020). Whispers, echoes, friends and fears: Forms and functions of voice-hearing in adolescence. *Child and Adolescent Mental Health*, *26*(3), 195–203. https://doi.org/10.1111/camh.12403

[18] Parry, S., Djabaeva, R., & Varese, F. (2018). *Engaging Young People Who Hear Voices in Online Mixed-Methods Research*. SAGE Publications Ltd. https://doi.org/10.4135/9781526457783

For peer review only

[19] Parry, S., Loren, E., & Varese, F. (2021). Young people's narratives of hearing voices: Systemic influences and conceptual challenges. *Clinical Psychology & Psychotherapy*, *28*(3), 715–726. https://doi.org/10.1002/cpp.2532

[20] Yang, L. H., Wonpat-Borja, A. J., Opler, M. G., & Corcoran, C. M. (2010). Potential stigma associated with inclusion of the psychosis risk syndrome in the DSM-V: An empirical question. *Schizophrenia Research*, *120*(1), 42–48. https://doi.org/10.1016/j.schres.2010.03.012

[21] Fusar-Poli, P., Cappucciati, M., Rutigliano, G., Schultze-Lutter, F., Bonoldi, I., Borgwardt, S., Riecher-Rössler, A., Addington, J., Perkins, D., Woods, S. W., McGlashan, T. H., Lee, J., Klosterkötter, J., Yung, A. R., & McGuire, P. (2015). At risk or not at risk? A meta-analysis of the prognostic accuracy of psychometric interviews for psychosis prediction. *World Psychiatry*, *14*(3), 322–332. https://doi.org/10.1002/wps.20250

[22] Hayward, M., John, M., Parry, S., Bibby-Jones, A.-M., Orchard, F., Malpass, F., Dixon, C., & Naito, A. (2025). Support for young people who are distressed by hearing voices: Protocol for an uncontrolled feasibility evaluation of a psychological intervention package delivered within secondary schools (the ECHOES study). *Pilot and Feasibility Studies*, *11*(1), 39. https://doi.org/10.1186/s40814-025-01611-x

[23] Parry, S., & Varese, F. (2021). "Listen to the parents… really listen to the child!" Family narratives of supporting children hearing voices. *Psychosis*, *13*(3), 209–219. https://doi.org/10.1080/17522439.2020.1856174

[24] Longden, E. (2017). Listening to the Voices People Hear: Auditory Hallucinations Beyond a Diagnostic Framework. *Journal of Humanistic Psychology*, *57*(6), 573–601. https://doi.org/10.1177/0022167817696838

For peer review only

[25] Alderson-Day, B., Woods, A., Moseley, P., Common, S., Deamer, F., Dodgson, G., & Fernyhough, C. (2021). Voice-Hearing and Personification: Characterizing Social Qualities of Auditory Verbal Hallucinations in Early Psychosis. *Schizophrenia Bulletin*, *47*(1), 228–236. https://doi.org/10.1093/schbul/sbaa095

[26] Jardri, R., Bartels-Velthuis, A. A., Debbané, M., Jenner, J. A., Kelleher, I., Dauvilliers, Y., Plazzi, G., Demeulemeester, M., David, C. N., Rapoport, J., Dobbelaere, D., Escher, S., & Fernyhough, C. (2014). From Phenomenology to Neurophysiological Understanding of Hallucinations in Children and Adolescents. *Schizophrenia Bulletin*, *40*(Suppl_4), S221–S232. https://doi.org/10.1093/schbul/sbu029

[27] Maijer, K., Begemann, M. J. H., Palmen, S. J. M. C., Leucht, S., & Sommer, I. E. C. (2018). Auditory hallucinations across the lifespan: A systematic review and meta-analysis. *Psychological Medicine*, *48*(6), 879–888. https://doi.org/10.1017/S0033291717002367

[28] Løberg, E.-M., Gjestad, R., Posserud, M.-B., Kompus, K., & Lundervold, A. J. (2019). Psychosocial characteristics differentiate non-distressing and distressing voices in 10,346 adolescents. *European Child & Adolescent Psychiatry*, *28*(10), 1353–1363. https://doi.org/10.1007/s00787-019-01292-x

[29] Maijer, K., Staring, T., Bartels-Velthuis, A. A., Palmen, S. J., & Sommer, I. E. (2020). Stronger than your voices: A cognitive behavioral therapy for youth suffering from auditory verbal hallucinations. *Clinical Child Psychology and Psychiatry*, *25*(2), 386–400. https://doi.org/10.1177/1359104519888011

[30] Maddox, L., Jolley, S., Laurens, K. R., Hirsch, C., Hodgins, S., Browning, S., Bravery, L., Bracegirdle, K., Smith, P., & Kuipers, E. (2013). Cognitive Behavioural Therapy for Unusual Experiences in Children: A Case Series. *Behavioural and Cognitive Psychotherapy*, *41*(3), 344–358. https://doi.org/10.1017/S1352465812000343

For peer review only

[31] Norman, D., Correia, H., & Paulik, G. (2020). An exploration of relationship between self-compassion and voice-related distress in people who hear voices. *Journal of Clinical Psychology*, *76*(10), 1984–1994. https://doi.org/10.1002/jclp.22975

[32] Heriot-Maitland, C., McCarthy-Jones, S., Longden, E., & Gilbert, P. (2019). Compassion Focused Approaches to Working With Distressing Voices. *Frontiers in Psychology*, *10*. https://doi.org/10.3389/fpsyg.2019.00152

[33] Nisar, A., Abid, G., Elahi, N. S., Ahsan Athar, M., & Farooqi, S. (2020). Impact of Compassion on Voice Behavior: A Moderated Mediation Model. *Journal of Open Innovation: Technology, Market, and Complexity*, *6*(4), 148. https://doi.org/10.3390/joitmc6040148

[34] Brown, K., & Parry, S. (2024). How do people with first episode psychosis experience therapeutic relationships with mental health practitioners? A narrative review. *Psychosis*, *16*(2), 155–166. https://doi.org/10.1080/17522439.2022.2160487

[35] Roberts, S., & Parry, S. (2023). Girl's and women's experiences of seeking mental health support for symptoms associated with psychosis. A narrative review. *Clinical Psychology & Psychotherapy*, *30*(2), 294–301. https://doi.org/10.1002/cpp.2819

[36] Rodell, S., & Parry, S. (2024). Family members' experiences of seeking help for a young person with symptoms associated with the psychosis spectrum: A narrative review and synthesis. *Clinical Child Psychology and Psychiatry*, *29*(3), 897–912. https://doi.org/10.1177/13591045231176701

[37] Leach, H., Kelly, J., & Parry, S. (2024). Compassion-informed approaches for coping with hearing voices: Literature review and narrative synthesis. *Psychosis*, *16*(3), 325–335. https://doi.org/10.1080/17522439.2023.2253883

[38] Dodgson et al. 2021 - Dodgson, G., Aynsworth, C., Mitrenga, K. J., Gibbs, C., Patton, V., Fernyhough, C., & Common, S. (2021). Managing unusual sensory experiences: a feasibility

For peer review only

trial in an at risk mental states for psychosis group. *Psychology and Psychotherapy: Theory, Research and Practice*, *94*(3), 481-503.

[39] Dudley et al., 2022 - Dudley, R., Dodgson, G., Common, S., O'Grady, L., Watson, F., Gibbs, C., & Aynsworth, C. (2022). Managing Unusual Sensory Experiences in People with First-Episode Psychosis (MUSE FEP): a study protocol for a single-blind parallel-group randomised controlled feasibility trial. *BMJ open*, *12*(5), e061827.

[40] Di Luzio, M., Pontillo, M., Di Vincenzo, C., Bellantoni, D., Demaria, F., & Vicari, S. (2022). Hallucinations in the Child and Adolescent "Ultra-High Risk" population: A Systematic Review. *Schizophrenia Bulletin Open*, *3*(1), sgac068. https://doi.org/10.1093/schizbullopen/sgac068

[41] D'Agnano, D., Lo Cascio, S., Correnti, E., Raieli, V., & Sciruicchio, V. (2023). A Narrative Review of Visual Hallucinations in Migraine and Epilepsy: Similarities and Differences in Children and Adolescents. *Brain Sciences*, *13*(4), 643. https://doi.org/10.3390/brainsci13040643

[42] Tang, S., Xie, Y., Wang, X., You, S., Fu, Y., & Wu, D. (2025). The prevalence and influencing factors of psychotic-like experiences in children and adolescents: A meta-analysis and systematic review. *European Child & Adolescent Psychiatry*. https://doi.org/10.1007/s00787-025-02905-4

[43] Nishiura, S., Miyawaki, D., Hirai, K., Sukigara, A., Kakishita, Y., & Inoue, K. (2024). Association between hallucinations and sensory processing difficulties in children and adolescents. *Frontiers in Psychiatry*, *15*. https://doi.org/10.3389/fpsyt.2024.1472328

[44] Fernyhough, C., de Boer, J., Davis, P. E., Schneider, M., Moriguchi, Y., Alderson-Day, B., Brederoo, S. G., & Jardri, R. (2025). Cognitive-Developmental Mechanisms in Hallucinations. *Schizophrenia Bulletin*, *51*(Supplement_3), S261–S272. https://doi.org/10.1093/schbul/sbaf144

For peer review only

[45] Kelleher, I. (2025). Annual Research Review: Psychosis in children and adolescents: key updates from the past 2 decades on psychotic disorders, psychotic experiences, and psychosis risk. *Journal of Child Psychology and Psychiatry*, *66*(4), 460–476. https://doi.org/10.1111/jcpp.14088

[46] Romelli, K., & Pozzi, G. O. (2018). Therapeutic community for children with diagnosis of psychosis: What place for parents? The relation between subject and the institutional 'Other'. In *Developments in Qualitative Psychotherapy Research*. Routledge.

[47] Claxton, M., Onwumere, J., & Fornells-Ambrojo, M. (2017). Do Family Interventions Improve Outcomes in Early Psychosis? A Systematic Review and Meta-Analysis. *Frontiers in Psychology*, *8*. https://doi.org/10.3389/fpsyg.2017.00371

[48] Kapur et al., 2014 - Kapur, P., Hayes, D., Waddingham, R., Hillman, S., Deighton, J., & Midgley, N. (2014). The experience of engaging with mental health services among young people who hear voices and their families: a mixed methods exploratory study. *BMC Health Services Research*, *14*(1), 527.

[49] Eldridge, S. M., Chan, C. L., Campbell, M. J., Bond, C. M., Hopewell, S., Thabane, L., & Lancaster, G. A. (2016). CONSORT 2010 statement: Extension to randomised pilot and feasibility trials. *BMJ*, *355*, i5239. https://doi.org/10.1136/bmj.i5239

[50] Lancaster et al., 2004 - Lancaster, G. A., Dodd, S., & Williamson, P. R. (2004). Design and analysis of pilot studies: recommendations for good practice. *Journal of evaluation in clinical practice*, *10*(2), 307-312.

[51] Teare, M. D., Dimairo, M., Shephard, N., Hayman, A., Whitehead, A., & Walters, S. J. (2014). Sample size requirements to estimate key design parameters from external pilot randomised controlled trials: A simulation study. *Trials*, *15*(1), 264. https://doi.org/10.1186/1745-6215-15-264

For peer review only

[52] Whitehead, A. L., Julious, S. A., Cooper, C. L., & Campbell, M. J. (2016). Estimating the sample size for a pilot randomised trial to minimise the overall trial sample size for the external pilot and main trial for a continuous outcome variable. *Statistical Methods in Medical Research*, *25*(3), 1057–1073. https://doi.org/10.1177/0962280215588241

[53] Chan, A. W., Tetzlaff, J. M., Gøtzsche, P. C., Altman, D. G., Mann, H., Berlin, J. A., ... & Moher, D. (2013). SPIRIT 2013 explanation and elaboration: guidance for protocols of clinical trials. *Bmj*, *346*. https://doi.org/10.1136/bmj.e7586

[54] Goodman, R., Meltzer, H., & Bailey, V. (1998). The Strengths and Difficulties Questionnaire: A pilot study on the validity of the self-report version. *European child & adolescent psychiatry*, *7*(3), 125-130.

[55] Mitchell, C. A., Maybery, M. T., Russell-Smith, S. N., Collerton, D., Gignac, G. E., & Waters, F. (2017). The structure and measurement of unusual sensory experiences in different modalities: the Multi-Modality Unusual Sensory Experiences Questionnaire (MUSEQ). *Frontiers in Psychology*, *8*, 1363. https://doi.org/10.3389/fpsyg.2017.01363.

[56] Liddle, I., & Carter, G. F. (2015). Emotional and psychological well-being in children: the development and validation of the Stirling Children's Well-being Scale. *Educational Psychology in Practice*, *31*(2), 174-185. https://doi.org/10.1080/02667363.2015.1008409

[57] Neff, K. D. (2003). The Development and Validation of a Scale to Measure Self-Compassion. *Self and Identity*, *2*(3), 223–250. https://doi.org/10.1080/15298860309027

[58] Spirito, A., Stark, L., & Williams, C. (1989). Development of a Brief Coping Checklist for Use with Pediatric Populations. *Journal of Pediatric Psychology*, *13*, 555–574. https://doi.org/10.1093/jpepsy/13.4.555

[59] Wille, N., Badia, X., Bonsel, G., Burström, K., Cavrini, G., Devlin, N., Egmar, A.-C., Greiner, W., Gusi, N., Herdman, M., Jelsma, J., Kind, P., Scalone, L., & Ravens-Sieberer, U. (2010).

For peer review only

Development of the EQ-5D-Y: A child-friendly version of the EQ-5D. *Quality of Life Research*, *19*(6), 875–886. https://doi.org/10.1007/s11136-010-9648-y

[60] Stevens, K. (2012). Valuation of the Child Health Utility 9D Index. *PharmacoEconomics*, *30*(8), 729–747. https://doi.org/10.2165/11599120-000000000-00000

[61] Austin, J. K., MacLeod, J., Dunn, D. W., Shen, J., & Perkins, S. M. (2004). Measuring stigma in children with epilepsy and their parents: instrument development and testing. *Epilepsy & Behavior*, *5*(4), 472-482. https://doi.org/10.1016/j.yebeh.2004.04.008.

[62] Craig, P., Dieppe, P., Macintyre, S., Michie, S., Nazareth, I., & Petticrew, M. (2008). *Developing and evaluating complex interventions: The new Medical Research Council guidance*. https://doi.org/10.1136/bmj.a1655

[63] NICE. (2013, January 23). *Overview | Psychosis and schizophrenia in children and young people: Recognition and management | Guidance | NICE*. https://www.nice.org.uk/guidance/cg155

[64] Waltz, J., Addis, M. E., Koerner, K., & Jacobson, N. S. (1993). Testing the integrity of a psychotherapy protocol: assessment of adherence and competence. *Journal of consulting and clinical psychology*, *61*(4), 620.

[65] Jolley, S., Browning, S., Corrigall, R., Laurens, K. R., Hirsch, C., Bracegirdle, K., ... & Emsley, R. (2017). Coping with Unusual ExperienceS for 12–18 year olds (CUES+): A transdiagnostic randomised controlled trial of the effectiveness of cognitive therapy in reducing distress associated with unusual experiences in adolescent mental health services: Study protocol for a randomised controlled trial. *Trials*, *18*(1), 586.

[66] Kazantzis, N., Whittington, C., Zelencich, L., Kyrios, M., Norton, P. J., & Hofmann, S. G. (2016). Quantity and quality of homework compliance: A meta-analysis of relations with outcome in cognitive behavior therapy. *Behavior Therapy*, *47*(5), 755-772.

For peer review only

[67] Stallard, P., Myles, P., & Branson, A. (2014). The cognitive behaviour therapy scale for children and young people (CBTS-CYP): Development and psychometric properties. *Behavioural and cognitive psychotherapy*, *42*(3), 269-282.

[68] Shirk, S. R., & Saiz, C. C. (1992). Clinical, empirical, and developmental perspectives on the therapeutic relationship in child psychotherapy. *Development and Psychopathology*, *4*(4), 713-728.

[69] Lee 2014 - Lee, E. C., Whitehead, A. L., Jacques, R. M., & Julious, S. A. (2014). The statistical interpretation of pilot trials: should significance thresholds be reconsidered?. *BMC medical research methodology*, *14*(1), 41.

[70] Devlin, N. J., & Brooks, R. (2017). EQ-5D and the EuroQol group: past, present and future. *Applied health economics and health policy*, *15*(2), 127-137.

[71] Martin-Kerry, J. M., Knapp, P., Atkin, K., Bower, P., Watt, I., Stones, C., ... & Young, B. (2019). Supporting children and young people when making decisions about joining clinical trials: qualitative study to inform multimedia website development. *BMJ open*, *9*(1), e023984. https://doi.org/10.1136/bmjopen-2018-023984

[72] Ritchie & Lewis 2003 – A better option: Spencer, L., Ritchie, J., Lewis, J., & Dillon, L. (2004). Quality in qualitative evaluation: a framework for assessing research evidence.

[73] Gale, N. K., Heath, G., Cameron, E., Rashid, S., & Redwood, S. (2013). Using the framework method for the analysis of qualitative data in multi-disciplinary health research. *BMC medical research methodology*, *13*(1), 117.

[74] Braun, V., & Clarke, V. (2022). Conceptual and design thinking for thematic analysis. *Qualitative psychology*, *9*(1), 3.

For peer review only

BMJ Open

The ChUSE Trial - Improving Access to Psychological Therapies for Children with Distressing Sensory Experiences in NHS CAMHS: A Randomised Controlled Feasibility Trial Protocol

Journal:	*BMJ Open*
Manuscript ID	bmjopen-2026-119727.R2
Article Type:	Protocol
Date Submitted by the Author:	15-Jul-2026
Complete List of Authors:	Parry, Sarah ; Pennine Care NHS Foundation Trust, Young People's Mental Health Research Centre; The University of Manchester, Division of Psychology and Mental Health | School of Health Sciences | Faculty of Biology<br>Carr, Sarah; Greater Manchester Mental Health NHS Foundation Trust, Complex Trauma and Resilience Research Unit<br>Carter, Lesley-Anne; The University of Manchester, Centre for Biostatistics, Division of Population Health, Health Services Research and Primary Care<br>Kirk, Sue; The University of Manchester, School of Health Sciences<br>Petrussa, Cinzia; Greater Manchester Mental Health NHS Foundation Trust, Complex Trauma and Resilience Research Unit<br>Malpass, Fiona; Voice Collective, Mind in Camden<br>Shields, Gemma; University of Manchester, Centre for Health Economics<br>Thompson, Alexander; The University of Manchester, Manchester Centre for Health Economics<br>Morrision, Tony; The University of Manchester, Division of Psychology and Mental Health; Greater Manchester Mental Health NHS Foundation Trust, Psychosis Research Unit<br>Varese, Filippo; The University of Manchester, Division of Psychology and Mental Health; Greater Manchester Mental Health NHS Foundation Trust, Complex Trauma and Resilience Research Unit
**Primary Subject Heading**:	Mental health
Secondary Subject Heading:	Evidence based practice, Health services research, Paediatrics, Research methods
Keywords:	MENTAL HEALTH, Child & adolescent psychiatry < PSYCHIATRY, Psychosocial Intervention, Clinical Protocols

The ChUSE Trial - Improving Access to Psychological Therapies for Children with Distressing Sensory Experiences in NHS CAMHS: A Randomised Controlled Feasibility Trial Protocol

For peer review only

Authors:

1. Dr Sarah Parry, University of Manchester, Division of Psychology and Mental Health and Pennine Care NHS Foundation Trust, Young People's Mental Health Research Centre, , 225 Old St, Ashton-under-Lyne OL6 7SR, UK Email: sarah.parry@manchester.ac.uk
2. Dr Sarah Carr, Greater Manchester Mental Health NHS Foundation Trust, Complex Trauma and Resilience Research Unit, Manchester, UK
3. Dr Lesley-Anne Carter, University of Manchester, Centre for Biostatistics, Division of Population Health, Health Service Research and Primary Care, Manchester, UK
4. Professor Sue Kirk, University of Manchester, School of Health Sciences, Manchester, UK
5. Cinzia Petrussa, Greater Manchester Mental Health NHS Foundation Trust, Complex Trauma and Resilience Research Unit, Manchester, UK
6. Fiona Malpass, Voice Collective, Mind in Camden, London, UK
7. Gemma Shields, University of Manchester, Centre for Health Economics, Manchester, UK
8. Dr Alexander Thompson, University of Manchester, Centre for Health Economics, Manchester, UK
9. Professor Tony Morrison, University of Manchester, Division of Psychology and Mental Health and Greater Manchester Mental Health NHS Foundation Trust, Psychosis Research Unit, Manchester, UK
10. Professor Filippo Varese, University of Manchester, Division of Psychology and Mental Health and Greater Manchester Mental Health NHS Foundation Trust, Complex Trauma and Resilience Research Unit, Manchester, UK

Corresponding Author: Dr Sarah Parry, University of Manchester and Young People's Mental Health Research Centre, Pennine Care NHS Foundation Trust. University of Manchester | Division of Psychology and Mental Health | School of Health Sciences | Faculty of Biology, Medicine and Health, M13 9PL, UK Email: sarah.parry@manchester.ac.uk

Abstract

Introduction

Distressing sensory experiences (DSEs) are commonly reported by children and young people accessing Child and Adolescent Mental Health Services (CAMHS) in the UK. Despite their prevalence, evidence-based, tailored interventions for this population are scarce. The ChUSE trial aims to address this gap by evaluating the feasibility and acceptability of a novel brief talking therapy for children aged 8-16 years old and an accompanying parent coaching programme.

Methods and Analysis

A single-blind, two-arm randomised controlled feasibility trial will be conducted across three NHS Trusts in Greater Manchester, UK. Sixty young people under the care of CAMHS experiencing DSEs and their parents/caregivers will be randomised 1:1 to receive either Treatment as Usual (TAU) or TAU plus the ChUSE intervention. The intervention involves four child therapy sessions and three optional parent sessions. Primary feasibility outcomes include recruitment and retention rates, treatment adherence, and intervention safety. Secondary objectives assess the feasibility of data collection procedures and gather qualitative insights into trial acceptability and the influence of the intervention upon young people's wellbeing.

Ethics and Dissemination

The ChUSE Trial has received ethical approval through the Health Research Authority following review by the West Midlands – South BirminghamResearch Ethics Committee specialising in paediatric research (ID 327343). The feasibility trial addresses a significant clinical gap in CAMHS provision and will also capture information as to current TAU practices, contributing novel data to guide future service provision, as TAU is currently understood to be heterogeneous. The information gathered through this trial will inform the design of a definitive trial evaluating the clinical effectiveness and cost-consequences of an early, age-appropriate intervention for DSEs. Findings and translational outputs will be shared through scientific and public forums to reach a variety of audiences.

Trial registration: ISRCTN12245618

Keywords: distressing sensory experiences, CAMHS, child mental health, cognitive therapy, compassionate mind training, early intervention, feasibility trial

Strengths and limitations of this study

1. Rigorous methodological design with clearly defined procedures that support transparency and replicability.
2. Systematic recruitment and sampling approach to minimise selection bias and enhance the credibility of the findings.
3. Use of validated data-collection tools, increasing reliability and consistency of measurement, paired with more novel and specialised measures to explore any phenomenological change.
4. Potential limitations in generalisability, as the sample is suitably small, so is unlikely to be representative of all relevant populations or contexts.
5. Risk of unmeasured confounding variables, which may influence interpretations despite efforts to control methodological bias.

For peer review only

Word count: 7,553 (not including abstract, references or checklists)

Improving Access to Psychological Therapies for Children with Distressing Sensory Experiences in NHS CAMHS: The ChUSE Trial Protocol

Introduction

Approximately half of children and young people attending Child and Adolescent Mental Health Services (CAMHS) report distressing sensory experiences (DSEs; [1]), including auditory and visual hallucinations, feelings of disconnection, and intrusive sensory thoughts [2,3]. Young people can experience DSEs due to the chronic stress of poverty[4] 4], bereavement, abuse and neglect [5][6], bullying [7], medical conditions such as epilepsy [8], adverse responses to medication [9], or disrupted sleep[10]. Distressing voice content has been associated with negative self-schemas and insecure attachments with caregivers [10]. Voice hearing is often transient in childhood and not usually a sign of severe mental illness, with DSEs (also referred to as multimodal hallucinations) typically less complex and less distressing in non-clinical samples [11, 12]. However, young people who experience even transient voice-hearing can report reduced quality of life at later follow-ups compared to their peers [13] and a higher rate of suicidal ideation [14], highlighting the potential long-term impact of voice hearing. Furthermore, DSEs in adolescence have been connected to later experiences of depression and anxiety [15], indicating a need for intervention as soon as possible to promote coping, wellbeing, and to prevent further difficulties.

A large-scale review [16] and primary research [17][18] indicate that many children begin to report unusual sensory experiences between the ages of 8 and 11 years, with 51% of a community sample describing these experiences as distressing [19]. From the age of 14, some young people may access specialist support through psychosis care pathways in the UK's National Health Service (NHS). However, referrals to such services can generate additional anxieties, potentially increasing self-stigma and adversely affecting wellbeing [20]. Current referral pathways for psychosis are not appropriate for many children experiencing DSEs, as only 15–22% of young people identified as at-risk for developing psychosis will access these pathways within a year [21]. This misalignment in referral pathways can increase anxiety for families and delay access to proportionate support in age-appropriate child and adolescent settings.

Providing earlier, non-psychosis-specific support within CAMHS could improve timely access to appropriate, proportionate interventions, while reducing future reliance on specialist services. Early intervention is crucial to promote coping strategies [22], enhance wellbeing [15], and to potentially mitigate the development of more severe conditions such as psychosis or mood disorders throughout the turbulent teenage years and into early adulthood.

Auditory verbal hallucinations are thought to be the most common multisensory experience, although this assumption is largely based on data with adults and young people aged 16 years and over. Voices can be comforting or distressing, and young people can experience both comforting and distressing voices at the same time [23], each sometimes taking on distinct identities[16,24,25]. Visual and tactile hallucinations can occur separately or concurrently and have been linked to trauma and/or anxiety [26, 27]. The impact of these multisensory experiences varies, influenced by

individual psychosocial factors, personal resources, and social support [28, 29]. Despite nearly 50% of children in CAMHS reporting multisensory experiences [1], there is currently no evidence-based talking therapy available for children with DSEs.

Young people have described benefitting from re-appraisal, normalizing [30], psychoeducation, externalisation [1], multisensory and cognitive restructuring coping strategies [17,19], similar to many of the approaches within cognitive therapies and compassionate mind training (CMT). Emerging findings suggest Compassion for Voices is a beneficial approach for young adult voice hearers, [31-33]. Our recent reviews have explored what is particularly important for young people with DSEs and first episode psychosis in therapeutic relationships [34], specific gender needs for girls when help-seeking [35], family members experiences of help-seeking for DSEs [36], and compassionate approaches to coping with voice hearing for young people [37]. These reviews recommend enhancing early support and communication about DSEs for young people, highlighting that girls and young women may experience more dismissive responses to their difficulties when they present to services, and the importance of collaborative therapeutic relationships and compassion-informed approaches for coping with hearing voices.

Drawing from the adult literature, a recent feasibility study on e-CBT for adults with DSEs found 4-6 sessions were acceptable for service users and progression criteria were met for recruitment, retention, and adherence to treatment [38]. Whilst it is positive to see the development of new interventions amongst the adult population [39], there remains a need for DSE research to be undertaken with young people in the unique setting of CAMHS, employing age-appropriate interventions [16]. Suitably tailored youth-focused interventions are limited in their availability and scientific evidence-base due to a scarcity of research with young people and their families to inform tailored protocols. Overall, research has overlooked younger children with DSEs which means important developmental and contextual information is currently missing from our understanding of how children experience DSEs and what support they and their families want from services.

Thus far, we know further work is needed with younger children to improve treatment pathways and reduce delays in accessing suitable support, and that clinicians desire clearer clinical guidance for tailored care [16]. Priorities for tailored care seem to include careful, developmentally informed assessment and caution around the use of structured interviews, which may insufficiently capture the form, function and content of DSEs[40]. Detailed clinical assessment is also important to distinguish between DSEs and hallucinations caused by neurological conditions such as migraine and occipital epilepsy [41]. Due to the role traumatic events [40]42]), substance use, sleep disorders, depression and anxiety may play in the experience of DSEs [42], a trauma-informed assessment may also be helpful where appropriate support can then be offered. Additionally, DSEs have been associated with sensory processing difficulties in paediatric samples for many years, although causal relationships and treatment implications remain unclear [43]. The ChUSE Trial is open to children in core CAMHS, autistic children, and children currently being assessed for neurodiversity in CAMHS. Adopting a neuroinclusive approach as early as possible in the treatment development process helps to ensure the final intervention is more acceptable and useful for all.

Developmental processes, including executive functioning, social cognition and language development, are thought to influence DSE phenomenology [44], although further research with younger children is needed to understand how to inform psychological support for DSEs. Overall, further research is needed to design developmentally sensitive assessments, interventions, treatment strategies, and to improve psychosocial outcomes [45]. Drawing on this emerging literature base and alongside lived experience consultations between 2016 and 2025, the ChUSE Trial aims to address recommendations for care in CAMHS by offering young people and their

For peer review only

parents/caregivers an intervention that mobilises existent knowledge to offer a tailored brief coping focused therapy, whilst assessing feasibility parameters to prepare for a definitive trial.

When supporting younger children with DSEs, it is essential to consider the role and involvement of parents and caregivers. Parental exclusion within services, and feelings of shame and guilt, can contribute towards relational instability within families [46]. Family interventions for psychosis (FIfP) have been found to improve cognitive functioning, reduce symptoms of psychosis, and reduce the likelihood of relapse [47]. Importantly, carer burden and wellbeing can also improve following a FIfP approach, indicating benefits for the wider family system of parental support, which may be especially relevant for younger children. The parent coaching sessions within the ChUSE Trial will offer a new and tailored approach for parents, offering separate spaces with a tailored therapeutic focus for parent and child.

In a recent review of parent's experiences of seeking help for young people with DSEs, we found many parents cannot find suitable care or are deterred from help-seeking following finding 'nothing on the other side of the door' of children's mental health services for DSEs, which can delay help-finding until the young person is in crisis [36]. Our primary research with parents and caregivers prior to the ChUSE Trial demonstrated parents want reliable information, skills and strategies to help their child cope [23]. Prior research identified that many families have dissatisfying experiences of help-seeking for hearing voices and would like more opportunities for normalising and holistic care [48]. Therefore, the ChUSE Trial meets an identified need in CAMHS, for children, practitioners and parents alike, offering a symptom-focused, low-intensity, systemic early-intervention, adding significantly to the clinical support for children and their parents and caregivers.

The ChUSE Trial

A significant challenge in treating DSEs for young people is the absence of child-centred research informing clinical guidelines, despite the recognised prevalence of these difficulties within CAMHS [1]. CAMHS practitioners frequently report missing the clinical guidance necessary to support this group of young people, resulting in restricted treatment options and delays in care for young people presenting with DSEs. To address this, the ChUSE trial will implement and evaluate an intervention protocol designed to offer early intervention for use within CAMHS. In time, this could provide practitioners with clear, practical guidance and ensure timely, appropriate support for young people. Informed by evidence from CBT interventions for adults and the few studies available that have involved children and young people, the ChUSE therapy integrates psychoeducation and therapeutic mechanisms from CBT and CMT to formulate young people's experiences of DSEs and identify potential coping strategies to reduce distress and enhancing coping for each young person.

A challenge for the ChUSE Trial is our limited understanding of what constitutes 'treatment as usual' (TAU) for this population. This adds complexity to the design and delivery of high-quality research in this area. To mitigate this, the ChUSE trial will incorporate a TAU arm, systematically gathering data on existing care practices across three NHS Trusts. This will provide essential information to inform the design of a future definitive trial.

For peer review only

Methods and Analysis

Aims and Objectives

Aim

To evaluate the feasibility and acceptability of conducting a future, larger-scale, definitive trial to assess the clinical effectiveness and cost-consequences of a brief psychological intervention for young people aged 8–16 years experiencing DSEs, accompanied by up to three parent coaching sessions.

Objectives

The primary objective is to assess key feasibility outcomes and gather data to inform the design of a future multi-site randomised controlled trial (RCT). Specifically, the study will examine:

- The feasibility of recruiting children and their parents or carers to the trial.
- The retention of participants throughout the trial period.
- Engagement with and adherence to the intervention and trial procedures.
- The safety of the intervention and trial procedures.
- Estimation of key parameters to support a sample size calculation for a future definitive efficacy trial.

Secondary Objectives

In addition to the primary feasibility outcomes, this study will address the following feasibility uncertainties:

- To determine the uptake of the optional parent coaching sessions.
- To evaluate the feasibility of collecting a set of proposed outcome measures, informing the selection of appropriate measures and data collection procedures for a future trial.
- To gather qualitative data exploring the acceptability of the intervention and trial procedures from the perspectives of young people, parents and carers, as well as their recommendations for refining the trial design.
- To collect qualitative insights from Child and Adolescent Mental Health Services (CAMHS) practitioners and clinical leads regarding barriers, facilitators, costs and consequences of implementing the intervention within routine CAMHS provision.

Primary Feasibility Outcomes

The main feasibility outcomes for this study are as follows:

- Recruitment rates, measured as the number of family units recruited and randomised per month.
- Participant retention rates, expressed as the proportion of participants completing outcome assessments at each time point.
- Treatment engagement and adherence, assessed by the number of sessions attended by young people and parents or carers allocated to both the ChUSE intervention plus treatment as usual (TAU) arm, and the TAU-only arm.

For peer review only

- Treatment fidelity, determined by the extent to which trial therapists deliver the ChUSE intervention according to the intervention protocol, as measured by fidelity checklists.
- Safety of the intervention, evaluated through the monitoring and recording of adverse events (AEs) and serious adverse events (SAEs), both related and unrelated to the intervention or trial procedures, using detailed AE reporting forms.
- Preliminary signals of efficacy and estimation of parameters required for a future sample size calculation, with outcome data collected at baseline (T0), three months (T1), and six months (T2).

Secondary Feasibility Outcomes

Secondary feasibility outcomes will include:

- Uptake of the parent coaching sessions, assessed through treatment engagement data, specifically the number of parents or carers who accept the offer of the PCS and the number of sessions attended.
- Feasibility of outcome data collection, determined by examining the completeness of the proposed outcome measures (percentage of missing responses and reasons for missing data), to guide measure selection and optimise data collection procedures for the future definitive trial.

Key secondary outcome measures

The following secondary outcome measures will be evaluated at baseline, T1 (three months), and T2 (six months) using a range of self-report measures collected from both children and young people (CYP) and their parents/guardians:

The 'promise of efficacy' of the ChUSE intervention and the necessary parameters to inform a sample size calculation for a future efficacy trial.

Measures for CYP

1. Symptom Level measured using:
1.1. Manchester Voices Inventory for Children (MAVIC)
1.2. Unusual Experiences Questionnaire (UEQ)
1.3. Brief Self-compassion Scale

2. Health-related quality of life outcomes measured using:
2.1. Stirling Children's Wellbeing Scale
2.2. EQ-5D-Y
2.3. KidCOPE scale
2.4. Child Health Utility Instrument (CHU-9D)
2.5. Strengths and Difficulties Questionnaire Self-Report version for 11-17 year olds

Measures for parents/caregivers

1. Health-related quality of life outcomes measured using:
1.1. SDQ-parent version
1.2. Adult Wellbeing Scale (parents/carers)

For peer review only

1.3. Parental Stress Scale (PSS), an 18-item questionnaire assessing parents' feelings about their parenting role, exploring positive [e.g. demands on resources, feelings of stress]
1.4. Coping Health Inventory for Parents (CHIP)
1.5. EQ-5D

Trial Design

This study will take the form of a feasibility randomised controlled trial (RCT) with an embedded process evaluation. It will employ a single-blind, parallel-group design, comprising two arms: (1) a brief psychological intervention involving four-six sessions of individual talking therapy for DSEs, with the optional offer of up to three sessions for parents/caregivers, in addition to treatment as usual (TAU); and (2) TAU alone. Participants in the intervention arm will also be offered a joint session zero with the trial therapist to discuss taking part in the intervention and to plan appointments. Outcome assessments will be conducted with all participants (N=60 parent-child dyads) at baseline (T0), at the end of the intervention (three months post-randomisation, T1), and at six months post-randomisation (T2). At T2, participants will be invited to take part in qualitative interviews to inform the process evaluation. Participants who do not wish to take part in qualitative interviews will be sent a brief online questionnaire via Qualtrics to enable participants to provide feedback on their experience of being in the trial and their experience of the intervention or CAMHS, depending on which arm of the trial they were randomised to. The trial is due to run between October 2024 and November 2026.

While the primary focus of the study is on feasibility outcomes, the process evaluation will generate both quantitative and qualitative data to enhance understanding of potential mechanisms of change, the acceptability of the intervention, preliminary indications of clinical promise within CAMHS settings as well as likely cost drivers and wider system consequences. Quantitative measures will help to track changes in symptoms and wellbeing over time, while qualitative feedback will capture participants’ experiences of the intervention, trial procedures, and how they have experienced their engagement in the trial. Qualitative interviews with practitioner (N=20) will also be conducted to inform interpretation of contextual factors and future implementation considerations.

Trial Setting

The trial will be conducted across multiple sites, with participant identification, recruitment, and treatment delivery taking place in outpatient CAMHS across three NHS Trusts in Greater Manchester. Should additional recruitment be required, participant identification may be extended to other comparable services within Trusts supported by the NIHR Research Delivery Network.

Patient and Public Involvement and Engagement (PPIE) and Equality, Diversity, Inclusion and Accessibility (EDIA)

This study has been co-developed with meaningful input from young people, parents and caregivers, and professionals with personal lived and clinical experience. A Lived Experience Advisory Group (LEAG), consisting of eight young people, will advise on the study throughout its duration. The LEAG has contributed to the design of study materials, intervention content and recruitment approaches through their involvement with Voice Collective, a lived experience led community group, to ensure relevance, accessibility, and sensitivity to lived experience. Alongside the LEAG, a Parent and

For peer review only

Caregiver Advisory Group comprising six members provides insights into family needs, language appropriateness, and acceptability of procedures from a caregiver's perspective.

A Stakeholder Advisory Group of ten professionals, including CAMHS clinicians, education specialists, and voluntary sector representatives, will offer strategic guidance on implementation, dissemination, and real-world relevance. Governance oversight will be provided by a Trial Steering Committee (TSC), which includes eight members with expertise in child mental health, trial methodology, and public involvement.

We are committed to upholding the principles of equality, diversity, inclusion, and accessibility (EDIA) across all components of the trial. Recruitment materials and procedures have been co-produced to ensure they are inclusive and appropriate for a range of cultural, linguistic, and neurodevelopmental needs. Monitoring of demographic data will help ensure transparency and accountability in terms of equitable access and identify any disparities in participation or outcomes.

Participants and Recruitment

This feasibility trial has been designed to be inclusive and accessible, with eligibility criteria and recruitment procedures shaped by extensive public and patient involvement and engagement (PPIE) and consultation with clinical stakeholders. Recognising that DSEs are not condition-specific, young people will not be excluded based on mental health diagnoses, co-occurring treatments, or neurodevelopmental status. The trial and intervention have been specifically designed to be inclusive of neurodiverse participants, and parents/caregivers are invited, but not required, to take part in the parent/caregiver sessions.

Eligibility Criteria

Children and young people (1) aged 8–16 years who are (2) currently receiving support from participating CAMHS and who (3) self-identify as experiencing DSEs will be eligible to participate. Given the absence of validated clinical thresholds for DSEs, eligibility will be confirmed using the Manchester Voices Inventory for Children (MAVIC) and the unusual sensory experiences questionnaire (MUSEQ) in screening to determine the presence of at least one self-identified current DSE, following initial referral. Although additional funding is not available across all locations, translators and interpreters will be sourced where funding from clinical services is available. If funding for this provision is not available, (4) the young person and their parent/caregiver will need to have conversational English to take part. (5) Current inpatient admission for acute mental health crisis, or (6) participation in another active psychological intervention trial, will also preclude people from taking part.

Participant Identification and Recruitment

Participants will be identified from existing CAMHS referrals where young people have self-reported DSEs. Research staff will regularly attend multidisciplinary team meetings to support clinicians in identifying eligible participants. Clinicians will provide initial study information to families and seek verbal consent for contact by the research team. The research team will subsequently contact interested families to provide full study details and obtain informed consent from parents/caregivers and young people aged 16 years and over, and assent from young people aged 15 years and under, documented in accordance with local governance procedures.

Screening and Enrolment

Following consent and assent, screening assessments using the MAVIC and MUSEQ measures will confirm eligibility prior to enrolment. Consenting participants will complete baseline assessments

For peer review only

(T0) before randomisation. Young people and parents/caregivers will be offered choices regarding the format and location of assessments (in-person, telephone, or video call) and whether to complete these independently or together.

Sampling and Recruitment Targets

As a feasibility trial, a formal power calculation is not required [49, 50]. In line with recommendations for pilot trials, we aim to randomise 60 young people and parent/caregiver dyads [51, 52]. This sample size is considered sufficient to estimate outcome measure variability and inform the design of a future definitive trial. Recruitment will take place over 19 months, with a minimum target of four participants per month in the first year. A recruitment review will be conducted at month 12, with contingency plans to extend to additional NHS Trusts if 75% of the target sample has not been achieved 6 months before the end of the trial.

Consent, Assent, and Withdrawal

Informed consent and assent will be obtained prior to participation in each stage of the study (T0, T1, T2). Participants may provide consent via signed hard copy, electronic form, or audio-recorded verbal consent, depending on preference. The consent process will be clearly documented, and participants will be reminded that participation is voluntary and can be withdrawn at any time without affecting their access to usual clinical care. If a participant withdraws but then wishes to opt back in, they may do so, if this is within four weeks of their six-month post-randomisation date for T2 data collection. Additional optional consent provisions will cover audio-recording assessments, use of anonymised data for secondary analysis, future research contact, teaching, and sharing study results. Based on media interest in our work in this area previously, there is also an option for participants to opt-in to receive information about future media enquiries or invitations. In line with the SPIRIT statement [53], predefined criteria for the discontinuation or interruption of the intervention will be applied at the individual participant level, with intervention delivery paused or possibly stopped where a serious adverse event (SAE) is assessed as being related to the ChUSE intervention procedures.

Remuneration and Expenses

Participants will receive a £10 shopping voucher as a thank you for completing outcome assessments at T0, T1 and T2. Reasonable travel expenses can be reimbursed upon request for in-person research appointments, although the majority of trial procedures will be conducted remotely or during routine clinic visits.

Measures

Eligibility Screening and Baseline Assessments

Eligibility will be confirmed via a short screening form (age and presence of at least one self-identified current distressing sensory experience using the MAVIC and MUSEQ). Those eligible will proceed to baseline (T0) assessments, including demographic, clinical history, and validated outcome measures. Ineligible participants will be debriefed, receive a voucher, and be offered a summary of their screening results to share with their clinical team.

Assessments will be flexible, delivered face-to-face or remotely, with options for breaks or multiple shorter sessions. After completing the baseline assessment, the young person (and, by proxy, their parent/caregiver) will be randomised to receive either TAU or ChUSE+TAU. Young people's preferences around who is present during assessment will be respected. Measures will be repeated at T1 and T2, as shown in table one and three.

For peer review only

Table 1: Assessments for children and young people

T0 (baseline)	T1 (3-month follow-up)	T2 (6-month follow-up)
Screen: MAVIC	MAVIC	MAVIC
Screen: Unusual Sensory Experiences Questionnaire	-	-
Demographics	-	-
Strengths and Difficulties Questionnaire (SDQ)	SDQ	SDQ
Kidcope Scale	Kidcope Scale	Kidcope Scale
The Stirling Children's Wellbeing Scale	The Stirling Children's Wellbeing Scale	The Stirling Children's Wellbeing Scale
Self-Compassion Scale Short Form (SCS-SF)	SCS-SF	SCS-SF
EQ-5D-Y	EQ-5D-Y	EQ-5D-Y
CHU-9D	CHU-9D	CHU-9D
CSS	CSS	CSS
		TAU checklist

Table 2: Measures and Completion Times

Instrument used in the proposed trial	Number of items	Indicative completion time
Strengths and Difficulties Questionnaire Self-Report version for 11–17-year-olds [54]	25	10-15 minutes
Manchester Voices Inventory for Children (MAVIC; [23])	18	10-12 minutes
Unusual Experiences Questionnaire (MUSEQ;[55])	9 (only issued at T0)	10-15 minutes
Stirling Children's Wellbeing Scale [56]	12	8-10 minutes
Brief Self-compassion Scale [57]	12	8-10 minutes
KidCOPE scale [58]	15	10-20 minutes
EQ-5D-Y [59]	5	5 minutes
Child Health Utility Instrument (CHU-9D; [60])	9	5-8 minutes
Child Stigma Scale (CSS) – adapted multisensory experiences [61]	7	5 minutes
Treatment as usual checklist (TAU checklist)	12	8-10 minutes

For peer review only

Table 3: Parent/carergiver measures

T0 (baseline)	T1 (3-month follow-up)	T2 (6-month follow-up)
Demographics	-	-
SDQ (parent version)	SDQ (parent version)	SDQ (parent version)
Adult Wellbeing Scale	Adult Wellbeing Scale	Adult Wellbeing Scale
Parental Stress Scale	Parental Stress Scale	Parental Stress Scale
Coping Health Inventory for Parents	Coping Health Inventory for Parents	Coping Health Inventory for Parents
EQ-5D	EQ-5D	EQ-5D

For peer review only

Participants do not have to complete all questions in the outcome measures. At the end of baseline assessment, participants will be informed of their trial arm allocation by a member of the research team.

Randomisation and Blinding

Participants will be randomly allocated on a 1:1 basis to either (1) ChUSE talking therapy plus parent sessions alongside TAU, or (2) TAU alone. Randomisation will be conducted independently using a web-based service (sealedenvelope.com), with concealed allocation via a pseudo-random sequence employing random permuted blocks of varying sizes, unknown to the study team.

Following baseline assessment, research workers will notify the Chief Investigator (CI) or a delegate about participant eligibility for randomisation. The CI or trial therapist (or delegate) will then enter the participant ID into the secure online system, which will immediately generate the allocation. Allocation outcomes will be communicated only to unblinded team members (CI, Co-CI, and the trial therapist) and centrally logged on the secure platform and a restricted-access randomisation log.

Blinding will be maintained for research workers and trainees responsible for data collection. Research workers and trainees will not access clinical notes or treatment information post-randomisation and will be routinely reminded, along with participants and clinical teams, to avoid disclosing treatment allocations during assessments. The CI, therapist, and co-investigators will remain unblinded in line with standard practice for psychological intervention trials. Any breaches of blinding will be documented, and where possible, follow-up will be reassigned to a blinded researcher.

Interventions

Comparator: Treatment as Usual (TAU)

Participants allocated to the TAU arm will continue to receive care as usual in CAMHS. The trial team will not intervene in routine care or request clinicians to withhold any referrals or treatments typically offered to participants. This aligns with established practices for pragmatic psychological intervention trials [62]. In the absence of national clinical guidelines specifically addressing DSEs for children, clinicians will be asked to describe what interventions and supports they provide to these young people and their families. These data will be systematically collected and tabulated using a

structured checklist and free-text responses, offering novel insights into routine clinical practices for DSE [63].

ChUSE Intervention

The feasibility trial will deliver the ChUSE intervention through a qualified, NHS-employed, HCPC-registered Clinical Psychologists, trained and supervised in the protocol by the study's Chief Investigator (CI). The manualised intervention will comprise of ideally seven 50-minute sessions (four child-focused and up to three parent coaching sessions) delivered over a three-month period. While the intervention will always remain brief, there is some flexibility in the number of sessions offered to young people to support engagement for up to a maximum of six sessions. This is to allow for pacing of content and accommodate breaks or shorter sessions as required by families with different needs. There will also be a session zero offered to all participants in the trial arm to attend as a child-parent/caregiver dyad. After each session, the therapist will complete an adherence checklist to monitor progression towards therapy milestones, the use of intervention strategies in line with the manual, and any deviations [64].

Parents/caregivers of children in the intervention arm will be offered the parent sessions alongside the child-focused therapy. Importantly, parental engagement in the parent sessions will not be a prerequisite for children to access individual therapy. The structure and delivery format of both child and parent components were informed by PPIE consultations, which recommended offering a choice of in-person or online sessions via Microsoft Teams to enhance accessibility.

ChUSE Child-Focused Intervention

The child-focused therapy is a psychological intervention targeting DSEs, delivered across four-six structured sessions. Content draws upon contemporary research on child distress, unusual sensory experiences, and psychosis-like phenomena.

- The first session will focus on exploring the young person's DSEs and goals, psychoeducation and normalisation [1, 30, 65].
- The second will introduce formulation and multisensory coping strategies based on compassion-focused therapy (CFT) principles, shaped by recommendations from young people with lived experience and the growing compassion for voices literature [17, 23, 32].
- The third session will include cognitive appraisal work, coping skills practice, and simple self-formulation exercises [31, 33].
- The final session will focus on compassion-focused reflection, consolidation of coping strategies, and safety/future planning.

ChUSE Parent Training Programme

Parents/caregivers will be invited to attend three structured sessions designed to complement the child's therapy.

- The first session offers psychoeducation, normalisation, reappraisal strategies, and therapist-guided, scenario-based problem-solving [19, 65].
- The second introduces compassion-focused coping techniques and self-kind containment strategies.

For peer review only

- The third provides therapist-guided reflective discussions, consolidating new knowledge and coping skills.

Between-session homework will involve reading psychoeducational materials and practising coping strategies, facilitating skills generalisation and enhancing therapeutic outcomes [66].

Fidelity and Process Monitoring

With participant consent, therapy sessions may be audio- or video-recorded for supervision and fidelity monitoring purposes. Declining to consent for recording will not affect the care received. Recordings will be destroyed following review or fidelity assessment.

Fidelity will be monitored via a therapist-completed intervention integrity checklist developed for the study. Additionally, a random sample of 10% of recorded sessions will be rated using the Cognitive Behaviour Therapy Scale for Children and Young People (CBT-SCYP) to assess adherence to the ChUSE manual [67]. The therapeutic alliance will also be measured post-intervention using the Therapeutic Alliance Scale for Children and Caregivers (TASC), completed by children, and parents/caregivers [68].

Follow-Up Assessments and Safeguarding Procedures

Participants will be invited to complete follow-up assessments at three months (T1) and six months (T2) post-randomisation. These assessments will replicate the baseline battery of measures, as shown in Tables One and Three. To enhance active safety monitoring, adverse events (AEs) will be systematically assessed during each contact point and through consultation with clinical notes.

Each assessment is expected to last approximately one hour, with options for breaks or multiple shorter sessions to accommodate participant's needs (see Table 2). T2 may take up to two hours, as this data point includes qualitative interviews. For all participants, there is then an option to provide further qualitative feedback through a Qualtrics survey, based on the same questions as the semi-structured interview guides. There is one survey for children and young people, and one for parents/caregivers. Upon completion of the final follow-up, participants will be formally debriefed about the feasibility RCT and, where consent has been provided, reminded about possible future contact for research updates or follow-up studies.

To minimise missing data, the research team will make multiple attempts to contact participants before considering them lost to follow-up. Data collection methods will include in-person, telephone, video conferencing, or postal options. Any spurious or incomplete data will be reviewed by the research team, who will determine appropriate actions such as verification or re-collection.

Safeguarding procedures will adhere to local and national guidelines. The Chief Investigator (CI) will be informed of any safeguarding concerns, and referrals will be made to the appropriate safeguarding team for the child's local area. In cases of immediate risk, the Emergency Duty Team will be contacted directly. Contact details for safeguarding services across Greater Manchester's ten boroughs will be held by designated trial staff, and procedures will follow the Greater Manchester Mental Health NHS Foundation Trust safeguarding policies.

Progression Criteria

Progression to a definitive trial will be determined using pre-specified feasibility criteria across five domains (see Table 4). Recruitment feasibility will be assessed by the number of parent–child dyads consented and randomised per month, alongside referral sources and reasons for non-eligibility or

non-participation. Feasibility will be demonstrated if an average of at least three dyads are recruited per month. Recruitment of two dyads per month will indicate conditional feasibility, requiring the identification of additional recruitment strategies, while recruitment averaging one participant per month (fewer than 20 participants overall) will indicate that progression is not feasible within the current design. However, these criteria will be applied with some flexibility where there are contextual factors to consider, such as the impact of school holidays and national holidays on family's engagement with services.

Intervention feasibility will be evaluated through adherence and attendance. Progression will be supported if at least 70% of participants allocated to the intervention arm complete the full course of treatment, defined as engagement with all four CYP sessions. Completion rates of 50–70% will indicate conditional feasibility, contingent on identifying barriers and potential adaptations informed by qualitative interviews and adherence data. Completion rates below 50% will indicate that progression to a definitive trial is not feasible without substantial modification of the intervention or delivery model.

Trial retention will be assessed by the proportion of participants completing end-of-treatment and follow-up assessments. Retention of at least 70% of participants will demonstrate feasibility. Retention rates between 30% and 70% will indicate conditional feasibility, requiring the development of strategies to address attrition, informed by reasons for withdrawal, recorded at the point of withdrawal in a participant tracker, and qualitative interview data. Retention below 30% will indicate non-feasibility of progression under the current trial design.

The feasibility of including the parent training programme in a future definitive trial will be determined by parental uptake and attendance. Inclusion of the parent sessions will be considered feasible if more than 70% of parents allocated to the intervention arm take up the offer. Uptake of 50–70% will indicate conditional feasibility, dependent on evidence of session attendance, potential added benefit, and health economic data suggesting cost-effectiveness. Uptake below 50% will indicate that inclusion of the parent sessions is not feasible within the current design.

Safety and acceptability of trial procedures will be assessed through monitoring of serious adverse events (SAEs). Feasibility will be demonstrated if fewer than 5% of participants experience SAEs related to trial procedures. SAE rates between 5% and 10% will indicate conditional feasibility, requiring the identification of additional risk-mitigation strategies informed by qualitative data. SAE rates exceeding 10% will indicate that progression to a definitive trial is not feasible. All SAEs will be reviewed by the Trial Steering Committee Chair and reported to the sponsor as appropriate.

Table 4: *Progression Criteria*

**Domain**	**Green**	**Amber**	**Red**
Recruitment	≥3 dyads/month	2 dyads/month	≤1/month (<20 total)
Intervention completion	≥70% complete	50–70%	<50%
Trial retention	≥70% retained	30–70%	<30%
Parent programme uptake	>70% uptake	50–70%	<50%
Safety (SAEs)	<5% SAE	5–10%	>10% SAE

For peer review only

Analysis of Key Feasibility Outcomes

Descriptive statistics will be employed to summarise key feasibility and acceptability outcomes, including trial recruitment, retention, therapy engagement, and treatment fidelity. Participant flow through the trial will be reported in accordance with the CONSORT extension for feasibility and pilot trials [49], detailing the number of referrals received per month, recruitment sources, number of participants contacted and assessed for eligibility, numbers consented (with reasons for ineligibility or declining participation when available), randomisation to trial arms, adherence to allocated interventions, and retention at the three- and six-month follow-up assessments.

All quantitative data analysis will be conducted according to a pre-specified statistical analysis plan prepared by our trial statistician (co-author LAC) and available upon request from the corresponding author. Descriptive analyses of therapy session records and fidelity checklists will estimate the adherence of therapists to the intervention protocol. Adverse events (AEs) and serious adverse events (SAEs) will be tabulated by trial arm and reviewed to assess the safety and acceptability of the intervention and study procedures.

In addition, descriptive statistics (mean, standard deviation, minimum, maximum, range, and percentage of missing data) will be reported for all outcome measures at baseline and follow-up assessments, disaggregated by trial arm. These data will inform feasibility parameters for a future definitive trial, including estimates of variance and preliminary outcome differences. No hypothesis testing for clinical effectiveness (p-values) will be conducted, but where appropriate, mean differences, standard deviations, and 80% confidence intervals will be calculated at follow-up time points to provide a preliminary indication of the 'promise of efficacy' of the ChUSE intervention on valued trial outcomes [69].

To support the feasibility of future health economic evaluation, the completeness and quality of data collected using the EQ-5D [70], EQ-5D-Y [59], and CHU-9D [60] will be summarised. Intervention and TAU service costs will be estimated using national unit cost data from NHS England and the Personal Social Services Research Unit.

Qualitative Evaluation

Interviews will be guided by topic guides co-developed with the PPIE groups and refined iteratively throughout the trial. Qualitative inquiry will be undertaken to explore participant and clinician experiences of trial processes and intervention delivery, which is essential for informing the development and future implementation of complex interventions [71]. Semi-structured interviews will be conducted by members of the research team at mutually convenient locations, either face-to-face, online via Microsoft Teams, or by telephone. Young people may be accompanied by a trusted adult if they wish.

Interviews will be audio-recorded, transcribed verbatim using secure transcription software, and subsequently reviewed and anonymised by a second member of the research team. Transcripts will be analysed using the Framework Method [72], a structured and transparent approach suitable for multidisciplinary health research teams [73]. Regular interpretation meetings will be held among the qualitative research team and LEAG to promote reflexivity and transparency during the analytic process.

For peer review only

Future access to anonymised final datasets will be considered case-by-case by the CI and Trial Sterring Committee, ensuring no risk of participant identification. Study findings will be disseminated through peer-reviewed publications, conference presentations, public forums, and participant summaries, in line with NIHR publication requirements.

Nested Validation Study: Manchester Voices Inventory for Children and Young People – Revised (MAVIC-R)

This nested validation study within the ChUSE Trial aims to assess the reliability, validity, and usability of the Manchester Voices Inventory for Children and Young People - Revised (MAVIC-R). MAVIC-R is a novel communication and assessment tool designed to help young people describe their unusual sensory experiences (USEs, which may be comforting, banal, or distressing) or DSEs in a non-pathologising, supportive manner. This study seeks to explore the tool's psychometric properties and explore its practical applications in NHS CAMHS and related services.

Research Questions

1. How effectively does MAVIC-R facilitate communication about DSEs with young people?
2. Which components are most helpful in exploring personal appraisals and meanings of DSEs?
3. How do young people, caregivers, and professionals perceive the tool's utility?
4. What are the practical and therapeutic implications of its use in CAMHS?

Research Design

Phase 1: MAVIC-R Refinement
Discussions with the ChUSE PPIE groups will inform updates to the existing MAVIC, expanding its scope from voice-hearing to multisensory experiences. Insights from these consultations will guide revisions to language and content.

Phase 2: Validation Study
A mixed-methods design will assess the psychometric properties and practical acceptability of the MAVIC-R:

- **Quantitative**: An online survey with young people will assess item properties and factor structure via Exploratory Factor Analysis (EFA).
- **Qualitative**: Mental health professionals will trial the tool in practice and provide feedback through interviews on its implementation and acceptability.

Participant Recruitment

- Up to 150 Young People (Aged 8–18) with lived experience of USEs, recruited through professional and lived experience networks, such as Voice Collective.
- 10 Parents/Caregivers of young people with USEs
- 10 Multiservice Professionals working with young people experiencing USEs

For peer review only

Inclusion Criteria

- Young people aged 8–18, fluent in conversational English, with personal experiences of one or more USEs
- Caregivers with current or past experience of supporting a young person with USEs
- Professionals with regular experience working with young people experiencing USEs

Exclusion Criteria

- Young people outside the specified age range, or whose current mental health prevents participation
- Professionals/caregivers without relevant experience
- Autistic young people whose USEs are solely attributed to sensory processing differences

Recruitment Process

Participants will be recruited via the RRDN, NHS networks, peer organisations (e.g. Voice Collective), and professional contacts. The same referral process as the wider ChUSE Trial will be followed, with practitioners completing brief referral forms with consent.

Consent/Assent

Participants will provide electronic consent via Qualtrics for surveys or audio-recorded consent for in-person interviews. For those under 16, caregiver consent and participant assent will be obtained.

Data Collection and Analysis

Audio-recorded interviews and focus groups will be transcribed and analysed thematically [74] using NVivo. Qualitative survey data will also be analysed thematically, and quantitative survey data will be analysed in SPSS. Data will be screened for missingness, item correlations examined, and sampling adequacy assessed via KMO and Bartlett's tests. EFA using Maximum Likelihood extraction with oblique rotation will determine factor structure, guided by scree plots, Kaiser's criterion, and parallel analysis. Reliability will be tested via Cronbach's alpha and item-total correlations.

Expected Outcomes and Dissemination

If validated, the MAVIC-R will provide a practical, non-stigmatising tool for facilitating discussions about USEs with young people, supporting tailored, person-centred care. Its use could foster more holistic, youth-informed care pathways within CAMHS and other youth services. Findings will be disseminated via peer-reviewed publications, stakeholder summaries, and multimedia resources for young people, families, and professionals. This study will also enable the inclusion of a validated, youth-specific measure in a future definitive ChUSE Trial.

Trial Endpoints, Ethical and Regulatory Considerations, and Data Management

The intervention period for this trial is scheduled to conclude by 1st March 2026, with all data collection for the trial completed by 1st June 2026. The nested validation study will be completed by the 1st December 2026. Prior to study commencement, the protocol, consent materials, and study documents will obtain favourable NHS REC and HRA approvals, with local Capacity and Capability agreements from participating NHS Trusts. The study will adhere to the Declaration of Helsinki, Good Clinical Practice, and the UK Policy Framework for Health and Social Care Research (2017).

For peer review only

Regulatory compliance will be maintained through appropriate approvals for all participating sites, timely submission of amendments, and regular oversight from the Sponsor and TSC. Protocol deviations will be formally logged and corrective actions agreed upon collaboratively.

Risk management procedures will include bespoke SOPs for safeguarding, clear limits of confidentiality, procedures for managing participant distress, and strict lone working and infection control policies for face-to-face contact.

Adverse events (AEs) and serious adverse events (SAEs) will be monitored closely, recorded according to established SOPs, and reviewed for severity, relatedness, expectedness, and seriousness. SAEs that are unexpected and related will be reported to the REC and Sponsor in line with standard HRA timelines.

Data management will comply with the Data Protection Act 2018 and GDPR. All personal data will be pseudonymised, securely stored, and deleted or anonymised at study end. Research data will be stored on encrypted servers, with access restricted to authorised study personnel. Final datasets and supporting materials will be archived securely with the Sponsor.

Ethics and Dissemination

The ChUSE Trial received ethical approval on 2nd May 2024 through the Health Research Authority following review by the West Midlands - South Birmingham Research Ethics Committee (REC) specialising in paediatric research (ID 327343). Any amendments to the protocol will be submitted to the REC committee as modifications. This study was registered with the ISRCTN on 14th June 2024 (https://www.isrctn.com/ISRCTN12245618).

Study findings will be widely disseminated through Open Access publications and presentations at national and international conferences. alongside more accessible multimedia disseminations developed in conjunction with our PPIE group such as podcasts, animations, infographics and youth friendly summaries. In parallel, findings will be disseminated through policy briefings and stakeholder events to inform national guidance and service development, with the aim of influencing policy and practice in child and adolescent mental health care. If successful, this study has the potential to inform clinical guidelines, contribute to workforce development through training and supervision models, and ultimately improve the quality and safety of services offered to young people in NHS mental health settings.

Patient and Public Involvement

Public engagement has been central to the development and design of this study, with young people, families, and practitioners contributing to intervention refinement, study materials, and data collection processes. Their input ensures that the research is sensitive to the needs and preferences of those it aims to support, and that dissemination plans are inclusive and accessible. We will endeavour to adopt the multisensory approach of the ChUSE intervention into the development of multisensory and multimedia translational outputs from the study, suitable and engaging for a range of audiences, in collaboration with our advisory groups.

For peer review only

Dissemination activities will be designed to engage a range of audiences, including young people, families, the public, and professionals across health, education, and voluntary sectors. A co-produced multimedia portfolio, including short animations, infographics, podcasts, and youth-friendly summaries, will support accessible communication of findings. These outputs will be developed in collaboration with the LEAG and shared through digital platforms, schools, CAMHS services, and third-sector networks.

Declarations: Ethics approval and consent to participate: Approved by West Midlands – South Birmingham NHS Research Ethics Committee (Ref: IRAS 327343). This study was funded by the National Institute for Health and Care Research (NIHR). The views expressed are those of the authors and not necessarily those of the NIHR or the Department of Health and Social Care.

Consent for publication: Not applicable.

Availability of data and materials: The datasets generated and/or analysed during this study will be available from the corresponding author on reasonable request.

Competing interests: The authors declare that they have no competing interests.

Funding: National Institute for Health and Care Research (NIHR) Research for Patient Benefit Programme (NIHR205326).

Author contributions: SP and FV conceived the study and developed the protocol. FM contributed to intervention development and PPI coordination. TM, SK, and DF provided methodological expertise. LAC advised on statistical analyses. GS and AT advised on health economics. All authors reviewed and approved the final manuscript.

Dr Sarah Parry is the author guarantor.

Acknowledgements: The authors thank the young people, parents, CAMHS practitioners, and members of the Lived Experience Advisory Group who contributed to the development of this protocol.

For peer review only

References

[1] Jolley, S., Kuipers, E., Stewart, C., Browning, S., Bracegirdle, K., Basit, N., Gin, K., Hirsch, C., Corrigall, R., Banerjea, P., Turley, G., Stahl, D., & Laurens, K. R. (2018). The Coping with Unusual Experiences for Children Study (CUES): A pilot randomized controlled evaluation of the acceptability and potential clinical utility of a cognitive behavioural intervention package for young people aged 8–14 years with unusual experiences and emotional symptoms. *British Journal of Clinical Psychology*, *57*(3), 328–350. https://doi.org/10.1111/bjc.12176

[2] Johns, L. C., Kompus, K., Connell, M., Humpston, C., Lincoln, T. M., Longden, E., Preti, A., Alderson-Day, B., Badcock, J. C., Cella, M., Fernyhough, C., McCarthy-Jones, S., Peters, E., Raballo, A., Scott, J., Siddi, S., Sommer, I. E., & Larøi, F. (2014). Auditory Verbal Hallucinations in Persons With and Without a Need for Care. *Schizophrenia Bulletin*, *40*(Suppl_4), S255–S264. https://doi.org/10.1093/schbul/sbu005

[3] Maijer, K., Palmen, S. J. M. C., & Sommer, I. E. C. (2017). Children seeking help for auditory verbal hallucinations; who are they? *Schizophrenia Research*, *183*, 31–35. https://doi.org/10.1016/j.schres.2016.10.033

[4] Hastings, P. D., Serbin, L. A., Bukowski, W., Helm, J. L., Stack, D. M., Dickson, D. J., Ledingham, J. E., & Schwartzman, A. E. (2020). Predicting psychosis-spectrum diagnoses in adulthood from social behaviors and neighborhood contexts in childhood. *Development and Psychopathology*, *32*(2), 465–479. https://doi.org/10.1017/S095457941900021X

[5] Corstens, D., & Longden, E. (2013). The origins of voices: Links between life history and voice hearing in a survey of 100 cases. *Psychosis*, *5*(3), 270–285. https://doi.org/10.1080/17522439.2013.816337

For peer review only

[6] Longden, E., Madill, A., & Waterman, M. G. (2012). Dissociation, trauma, and the role of lived experience: Toward a new conceptualization of voice hearing. *Psychological Bulletin*, *138*(1), 28–76. https://doi.org/10.1037/a0025995

[7] Domínguez, T., Nieto, L., Fresán, A., Sheinbaum, T., & Robles, R. (2025). The association of bullying and self-esteem with psychotic-like experiences and clinical high risk for psychosis symptoms. *Child and Adolescent Psychiatry and Mental Health*, *19*(1), 51. https://doi.org/10.1186/s13034-025-00900-w

[8] Bisulli, F., Menghi, V., Vignatelli, L., Licchetta, L., Zenesini, C., Stipa, C., Morigi, F., Gizzi, M., Avoni, P., Provini, F., Mostacci, B., d'Orsi, G., Pippucci, T., Muccioli, L., & Tinuper, P. (2018). Epilepsy with auditory features: Long-term outcome and predictors of terminal remission. *Epilepsia*, *59*(4), 834–843. https://doi.org/10.1111/epi.14033

[9] Capaldi, V. F., & Carr, R. B. (2010). Citalopram-induced hallucinations and delusions in a young adult. *General Hospital Psychiatry*, *32*(6), 648.e1-648.e3. https://doi.org/10.1016/j.genhosppsych.2010.07.008

[10] Waters, F., Blom, J. D., Jardri, R., Hugdahl, K., & Sommer, I. E. C. (2018). Auditory hallucinations, not necessarily a hallmark of psychotic disorder. *Psychological Medicine*, *48*(4), 529–536. https://doi.org/10.1017/S0033291717002203

[11] Medjkane, F., Notredame, C.-E., Sharkey, L., D'Hondt, F., Vaiva, G., & Jardri, R. (2020). Association between childhood trauma and multimodal early-onset hallucinations. *The British Journal of Psychiatry*, *216*(3), 156–158. https://doi.org/10.1192/bjp.2019.266

[12] Skokauskas, N., Pillay, D., Moran, T., & Kahn, D. A. (2010). Transient Auditory Hallucinations in an Adolescent. *Journal of Psychiatric Practice®*, *16*(3), 187. https://doi.org/10.1097/01.pra.0000375716.77831.ca

For peer review only

[13] Calkins, M. E., Moore, T. M., Satterthwaite, T. D., Wolf, D. H., Turetsky, B. I., Roalf, D. R., Merikangas, K. R., Ruparel, K., Kohler, C. G., Gur, R. C., & Gur, R. E. (2017). Persistence of psychosis spectrum symptoms in the Philadelphia Neurodevelopmental Cohort: A prospective two-year follow-up. *World Psychiatry*, *16*(1), 62–76. https://doi.org/10.1002/wps.20386

[14] Fujita, J., Takahashi, Y., Nishida, A., Okumura, Y., Ando, S., Kawano, M., Toyohara, K., Sho, N., Minami, T., & Arai, T. (2015). Auditory verbal hallucinations increase the risk for suicide attempts in adolescents with suicidal ideation. *Schizophrenia Research*, *168*(1), 209–212. https://doi.org/10.1016/j.schres.2015.07.028

[15] Kelleher, I., & Cannon, M. (2011). Psychotic-like experiences in the general population: Characterizing a high-risk group for psychosis. *Psychological Medicine*, *41*(1), 1–6. https://doi.org/10.1017/S0033291710001005

[16] Maijer, K., Hayward, M., Fernyhough, C., Calkins, M. E., Debbané, M., Jardri, R., Kelleher, I., Raballo, A., Rammou, A., Scott, J. G., Shinn, A. K., Steenhuis, L. A., Wolf, D. H., & Bartels-Velthuis, A. A. (2019). Hallucinations in Children and Adolescents: An Updated Review and Practical Recommendations for Clinicians. *Schizophrenia Bulletin*, *45*(Supplement_1), S5–S23. https://doi.org/10.1093/schbul/sby119

[17] Parry, S., & Varese, F. (2020). Whispers, echoes, friends and fears: Forms and functions of voice-hearing in adolescence. *Child and Adolescent Mental Health*, *26*(3), 195–203. https://doi.org/10.1111/camh.12403

[18] Parry, S., Djabaeva, R., & Varese, F. (2018). *Engaging Young People Who Hear Voices in Online Mixed-Methods Research*. SAGE Publications Ltd. https://doi.org/10.4135/9781526457783

For peer review only

[19] Parry, S., Loren, E., & Varese, F. (2021). Young people's narratives of hearing voices: Systemic influences and conceptual challenges. *Clinical Psychology & Psychotherapy*, *28*(3), 715–726. https://doi.org/10.1002/cpp.2532

[20] Yang, L. H., Wonpat-Borja, A. J., Opler, M. G., & Corcoran, C. M. (2010). Potential stigma associated with inclusion of the psychosis risk syndrome in the DSM-V: An empirical question. *Schizophrenia Research*, *120*(1), 42–48. https://doi.org/10.1016/j.schres.2010.03.012

[21] Fusar-Poli, P., Cappucciati, M., Rutigliano, G., Schultze-Lutter, F., Bonoldi, I., Borgwardt, S., Riecher-Rössler, A., Addington, J., Perkins, D., Woods, S. W., McGlashan, T. H., Lee, J., Klosterkötter, J., Yung, A. R., & McGuire, P. (2015). At risk or not at risk? A meta-analysis of the prognostic accuracy of psychometric interviews for psychosis prediction. *World Psychiatry*, *14*(3), 322–332. https://doi.org/10.1002/wps.20250

[22] Hayward, M., John, M., Parry, S., Bibby-Jones, A.-M., Orchard, F., Malpass, F., Dixon, C., & Naito, A. (2025). Support for young people who are distressed by hearing voices: Protocol for an uncontrolled feasibility evaluation of a psychological intervention package delivered within secondary schools (the ECHOES study). *Pilot and Feasibility Studies*, *11*(1), 39. https://doi.org/10.1186/s40814-025-01611-x

[23] Parry, S., & Varese, F. (2021). "Listen to the parents… really listen to the child!" Family narratives of supporting children hearing voices. *Psychosis*, *13*(3), 209–219. https://doi.org/10.1080/17522439.2020.1856174

[24] Longden, E. (2017). Listening to the Voices People Hear: Auditory Hallucinations Beyond a Diagnostic Framework. *Journal of Humanistic Psychology*, *57*(6), 573–601. https://doi.org/10.1177/0022167817696838

For peer review only

[25] Alderson-Day, B., Woods, A., Moseley, P., Common, S., Deamer, F., Dodgson, G., & Fernyhough, C. (2021). Voice-Hearing and Personification: Characterizing Social Qualities of Auditory Verbal Hallucinations in Early Psychosis. *Schizophrenia Bulletin*, *47*(1), 228–236. https://doi.org/10.1093/schbul/sbaa095

[26] Jardri, R., Bartels-Velthuis, A. A., Debbané, M., Jenner, J. A., Kelleher, I., Dauvilliers, Y., Plazzi, G., Demeulemeester, M., David, C. N., Rapoport, J., Dobbelaere, D., Escher, S., & Fernyhough, C. (2014). From Phenomenology to Neurophysiological Understanding of Hallucinations in Children and Adolescents. *Schizophrenia Bulletin*, *40*(Suppl_4), S221–S232. https://doi.org/10.1093/schbul/sbu029

[27] Maijer, K., Begemann, M. J. H., Palmen, S. J. M. C., Leucht, S., & Sommer, I. E. C. (2018). Auditory hallucinations across the lifespan: A systematic review and meta-analysis. *Psychological Medicine*, *48*(6), 879–888. https://doi.org/10.1017/S0033291717002367

[28] Løberg, E.-M., Gjestad, R., Posserud, M.-B., Kompus, K., & Lundervold, A. J. (2019). Psychosocial characteristics differentiate non-distressing and distressing voices in 10,346 adolescents. *European Child & Adolescent Psychiatry*, *28*(10), 1353–1363. https://doi.org/10.1007/s00787-019-01292-x

[29] Maijer, K., Staring, T., Bartels-Velthuis, A. A., Palmen, S. J., & Sommer, I. E. (2020). Stronger than your voices: A cognitive behavioral therapy for youth suffering from auditory verbal hallucinations. *Clinical Child Psychology and Psychiatry*, *25*(2), 386–400. https://doi.org/10.1177/1359104519888011

[30] Maddox, L., Jolley, S., Laurens, K. R., Hirsch, C., Hodgins, S., Browning, S., Bravery, L., Bracegirdle, K., Smith, P., & Kuipers, E. (2013). Cognitive Behavioural Therapy for Unusual Experiences in Children: A Case Series. *Behavioural and Cognitive Psychotherapy*, *41*(3), 344–358. https://doi.org/10.1017/S1352465812000343

For peer review only

[31] Norman, D., Correia, H., & Paulik, G. (2020). An exploration of relationship between self-compassion and voice-related distress in people who hear voices. *Journal of Clinical Psychology*, *76*(10), 1984–1994. https://doi.org/10.1002/jclp.22975

[32] Heriot-Maitland, C., McCarthy-Jones, S., Longden, E., & Gilbert, P. (2019). Compassion Focused Approaches to Working With Distressing Voices. *Frontiers in Psychology*, *10*. https://doi.org/10.3389/fpsyg.2019.00152

[33] Nisar, A., Abid, G., Elahi, N. S., Ahsan Athar, M., & Farooqi, S. (2020). Impact of Compassion on Voice Behavior: A Moderated Mediation Model. *Journal of Open Innovation: Technology, Market, and Complexity*, *6*(4), 148. https://doi.org/10.3390/joitmc6040148

[34] Brown, K., & Parry, S. (2024). How do people with first episode psychosis experience therapeutic relationships with mental health practitioners? A narrative review. *Psychosis*, *16*(2), 155–166. https://doi.org/10.1080/17522439.2022.2160487

[35] Roberts, S., & Parry, S. (2023). Girl's and women's experiences of seeking mental health support for symptoms associated with psychosis. A narrative review. *Clinical Psychology & Psychotherapy*, *30*(2), 294–301. https://doi.org/10.1002/cpp.2819

[36] Rodell, S., & Parry, S. (2024). Family members' experiences of seeking help for a young person with symptoms associated with the psychosis spectrum: A narrative review and synthesis. *Clinical Child Psychology and Psychiatry*, *29*(3), 897–912. https://doi.org/10.1177/13591045231176701

[37] Leach, H., Kelly, J., & Parry, S. (2024). Compassion-informed approaches for coping with hearing voices: Literature review and narrative synthesis. *Psychosis*, *16*(3), 325–335. https://doi.org/10.1080/17522439.2023.2253883

[38] Dodgson et al. 2021 - Dodgson, G., Aynsworth, C., Mitrenga, K. J., Gibbs, C., Patton, V., Fernyhough, C., & Common, S. (2021). Managing unusual sensory experiences: a feasibility

For peer review only

trial in an at risk mental states for psychosis group. *Psychology and Psychotherapy: Theory, Research and Practice*, *94*(3), 481-503.

[39] Dudley et al., 2022 - Dudley, R., Dodgson, G., Common, S., O'Grady, L., Watson, F., Gibbs, C., & Aynsworth, C. (2022). Managing Unusual Sensory Experiences in People with First-Episode Psychosis (MUSE FEP): a study protocol for a single-blind parallel-group randomised controlled feasibility trial. *BMJ open*, *12*(5), e061827.

[40] Di Luzio, M., Pontillo, M., Di Vincenzo, C., Bellantoni, D., Demaria, F., & Vicari, S. (2022). Hallucinations in the Child and Adolescent "Ultra-High Risk" population: A Systematic Review. *Schizophrenia Bulletin Open*, *3*(1), sgac068. https://doi.org/10.1093/schizbullopen/sgac068

[41] D'Agnano, D., Lo Cascio, S., Correnti, E., Raieli, V., & Sciruicchio, V. (2023). A Narrative Review of Visual Hallucinations in Migraine and Epilepsy: Similarities and Differences in Children and Adolescents. *Brain Sciences*, *13*(4), 643. https://doi.org/10.3390/brainsci13040643

[42] Tang, S., Xie, Y., Wang, X., You, S., Fu, Y., & Wu, D. (2025). The prevalence and influencing factors of psychotic-like experiences in children and adolescents: A meta-analysis and systematic review. *European Child & Adolescent Psychiatry*. https://doi.org/10.1007/s00787-025-02905-4

[43] Nishiura, S., Miyawaki, D., Hirai, K., Sukigara, A., Kakishita, Y., & Inoue, K. (2024). Association between hallucinations and sensory processing difficulties in children and adolescents. *Frontiers in Psychiatry*, *15*. https://doi.org/10.3389/fpsyt.2024.1472328

[44] Fernyhough, C., de Boer, J., Davis, P. E., Schneider, M., Moriguchi, Y., Alderson-Day, B., Brederoo, S. G., & Jardri, R. (2025). Cognitive-Developmental Mechanisms in Hallucinations. *Schizophrenia Bulletin*, *51*(Supplement_3), S261–S272. https://doi.org/10.1093/schbul/sbaf144

For peer review only

[45] Kelleher, I. (2025). Annual Research Review: Psychosis in children and adolescents: key updates from the past 2 decades on psychotic disorders, psychotic experiences, and psychosis risk. *Journal of Child Psychology and Psychiatry*, *66*(4), 460–476. https://doi.org/10.1111/jcpp.14088

[46] Romelli, K., & Pozzi, G. O. (2018). Therapeutic community for children with diagnosis of psychosis: What place for parents? The relation between subject and the institutional 'Other'. In *Developments in Qualitative Psychotherapy Research*. Routledge.

[47] Claxton, M., Onwumere, J., & Fornells-Ambrojo, M. (2017). Do Family Interventions Improve Outcomes in Early Psychosis? A Systematic Review and Meta-Analysis. *Frontiers in Psychology*, *8*. https://doi.org/10.3389/fpsyg.2017.00371

[48] Kapur et al., 2014 - Kapur, P., Hayes, D., Waddingham, R., Hillman, S., Deighton, J., & Midgley, N. (2014). The experience of engaging with mental health services among young people who hear voices and their families: a mixed methods exploratory study. *BMC Health Services Research*, *14*(1), 527.

[49] Eldridge, S. M., Chan, C. L., Campbell, M. J., Bond, C. M., Hopewell, S., Thabane, L., & Lancaster, G. A. (2016). CONSORT 2010 statement: Extension to randomised pilot and feasibility trials. *BMJ*, *355*, i5239. https://doi.org/10.1136/bmj.i5239

[50] Lancaster et al., 2004 - Lancaster, G. A., Dodd, S., & Williamson, P. R. (2004). Design and analysis of pilot studies: recommendations for good practice. *Journal of evaluation in clinical practice*, *10*(2), 307-312.

[51] Teare, M. D., Dimairo, M., Shephard, N., Hayman, A., Whitehead, A., & Walters, S. J. (2014). Sample size requirements to estimate key design parameters from external pilot randomised controlled trials: A simulation study. *Trials*, *15*(1), 264. https://doi.org/10.1186/1745-6215-15-264

For peer review only

[52] Whitehead, A. L., Julious, S. A., Cooper, C. L., & Campbell, M. J. (2016). Estimating the sample size for a pilot randomised trial to minimise the overall trial sample size for the external pilot and main trial for a continuous outcome variable. *Statistical Methods in Medical Research*, *25*(3), 1057–1073. https://doi.org/10.1177/0962280215588241

[53] Chan, A. W., Tetzlaff, J. M., Gøtzsche, P. C., Altman, D. G., Mann, H., Berlin, J. A., ... & Moher, D. (2013). SPIRIT 2013 explanation and elaboration: guidance for protocols of clinical trials. *Bmj*, *346*. https://doi.org/10.1136/bmj.e7586

[54] Goodman, R., Meltzer, H., & Bailey, V. (1998). The Strengths and Difficulties Questionnaire: A pilot study on the validity of the self-report version. *European child & adolescent psychiatry*, *7*(3), 125-130.

[55] Mitchell, C. A., Maybery, M. T., Russell-Smith, S. N., Collerton, D., Gignac, G. E., & Waters, F. (2017). The structure and measurement of unusual sensory experiences in different modalities: the Multi-Modality Unusual Sensory Experiences Questionnaire (MUSEQ). *Frontiers in Psychology*, *8*, 1363. https://doi.org/10.3389/fpsyg.2017.01363.

[56] Liddle, I., & Carter, G. F. (2015). Emotional and psychological well-being in children: the development and validation of the Stirling Children's Well-being Scale. *Educational Psychology in Practice*, *31*(2), 174-185. https://doi.org/10.1080/02667363.2015.1008409

[57] Neff, K. D. (2003). The Development and Validation of a Scale to Measure Self-Compassion. *Self and Identity*, *2*(3), 223–250. https://doi.org/10.1080/15298860309027

[58] Spirito, A., Stark, L., & Williams, C. (1989). Development of a Brief Coping Checklist for Use with Pediatric Populations. *Journal of Pediatric Psychology*, *13*, 555–574. https://doi.org/10.1093/jpepsy/13.4.555

[59] Wille, N., Badia, X., Bonsel, G., Burström, K., Cavrini, G., Devlin, N., Egmar, A.-C., Greiner, W., Gusi, N., Herdman, M., Jelsma, J., Kind, P., Scalone, L., & Ravens-Sieberer, U. (2010).

For peer review only

Development of the EQ-5D-Y: A child-friendly version of the EQ-5D. *Quality of Life Research*, *19*(6), 875–886. https://doi.org/10.1007/s11136-010-9648-y

[60] Stevens, K. (2012). Valuation of the Child Health Utility 9D Index. *PharmacoEconomics*, *30*(8), 729–747. https://doi.org/10.2165/11599120-000000000-00000

[61] Austin, J. K., MacLeod, J., Dunn, D. W., Shen, J., & Perkins, S. M. (2004). Measuring stigma in children with epilepsy and their parents: instrument development and testing. *Epilepsy & Behavior*, *5*(4), 472-482. https://doi.org/10.1016/j.yebeh.2004.04.008.

[62] Craig, P., Dieppe, P., Macintyre, S., Michie, S., Nazareth, I., & Petticrew, M. (2008). *Developing and evaluating complex interventions: The new Medical Research Council guidance*. https://doi.org/10.1136/bmj.a1655

[63] NICE. (2013, January 23). *Overview | Psychosis and schizophrenia in children and young people: Recognition and management | Guidance | NICE*. https://www.nice.org.uk/guidance/cg155

[64] Waltz, J., Addis, M. E., Koerner, K., & Jacobson, N. S. (1993). Testing the integrity of a psychotherapy protocol: assessment of adherence and competence. *Journal of consulting and clinical psychology*, *61*(4), 620.

[65] Jolley, S., Browning, S., Corrigall, R., Laurens, K. R., Hirsch, C., Bracegirdle, K., ... & Emsley, R. (2017). Coping with Unusual ExperienceS for 12–18 year olds (CUES+): A transdiagnostic randomised controlled trial of the effectiveness of cognitive therapy in reducing distress associated with unusual experiences in adolescent mental health services: Study protocol for a randomised controlled trial. *Trials*, *18*(1), 586.

[66] Kazantzis, N., Whittington, C., Zelencich, L., Kyrios, M., Norton, P. J., & Hofmann, S. G. (2016). Quantity and quality of homework compliance: A meta-analysis of relations with outcome in cognitive behavior therapy. *Behavior Therapy*, *47*(5), 755-772.

For peer review only

[67] Stallard, P., Myles, P., & Branson, A. (2014). The cognitive behaviour therapy scale for children and young people (CBTS-CYP): Development and psychometric properties. *Behavioural and cognitive psychotherapy*, *42*(3), 269-282.

[68] Shirk, S. R., & Saiz, C. C. (1992). Clinical, empirical, and developmental perspectives on the therapeutic relationship in child psychotherapy. *Development and Psychopathology*, *4*(4), 713-728.

[69] Lee 2014 - Lee, E. C., Whitehead, A. L., Jacques, R. M., & Julious, S. A. (2014). The statistical interpretation of pilot trials: should significance thresholds be reconsidered?. *BMC medical research methodology*, *14*(1), 41.

[70] Devlin, N. J., & Brooks, R. (2017). EQ-5D and the EuroQol group: past, present and future. *Applied health economics and health policy*, *15*(2), 127-137.

[71] Martin-Kerry, J. M., Knapp, P., Atkin, K., Bower, P., Watt, I., Stones, C., ... & Young, B. (2019). Supporting children and young people when making decisions about joining clinical trials: qualitative study to inform multimedia website development. *BMJ open*, *9*(1), e023984. https://doi.org/10.1136/bmjopen-2018-023984

[72] Ritchie & Lewis 2003 – A better option: Spencer, L., Ritchie, J., Lewis, J., & Dillon, L. (2004). Quality in qualitative evaluation: a framework for assessing research evidence.

[73] Gale, N. K., Heath, G., Cameron, E., Rashid, S., & Redwood, S. (2013). Using the framework method for the analysis of qualitative data in multi-disciplinary health research. *BMC medical research methodology*, *13*(1), 117.

[74] Braun, V., & Clarke, V. (2022). Conceptual and design thinking for thematic analysis. *Qualitative psychology*, *9*(1), 3.

For peer review only
