# Supplementary material for: The ChUSE trial - improving access to psychological therapies for children with distressing sensory experiences in NHS CAMHS: a randomised controlled feasibility trial protocol
Source: BMJ Open. 2026 Sep 10;16(9):e119727. doi: 10.1136/bmjopen-2026-119727 (PMC13584016; doi:10.1136/bmjopen-2026-119727)
Supplement: Supplementary file 2 [file bmjopen-2026-119727.reviewer_comments.pdf]

PEER REVIEW HISTORY

BMJ Open publishes all reviews undertaken for accepted manuscripts. Reviewers are asked to complete a checklist review form and are provided with free text boxes to elaborate on their assessment. These free text comments are reproduced below.

ARTICLE DETAILS

Title (Provisional)

The ChUSE Trial - Improving Access to Psychological Therapies for Children with Distressing Sensory Experiences in NHS CAMHS: A Randomised Controlled Feasibility Trial Protocol

Authors

Parry, Sarah; Carr, Sarah; Carter, Lesley-Anne; Kirk, Sue; Petrussa, Cinzia; Malpass, Fiona; Shields, Gemma E; Thompson, Alexander; Morrision, Tony; Varese, Filippo

---

VERSION 1 - REVIEW

**Reviewer**	**1**
**Name**	**Jacobsen, Pamela**
**Affiliation**	**University of Bath Department of Psychology, Psychology**
**Date**	**29-Apr-2026**
**COI**	**None**

Thank you for the opportunity to review this protocol paper. The protocol is very thorough and well-written. The study addresses an area of clinical importance which has been much neglected so far, in developing and testing psychological interventions for children and adolescents with transdiagnostic distressing sensory experiences. It is also good to see a trial recruiting younger children as under-18s are underrepresented in health research in general and this means that a specific evidence base for this age group is lacking, and children often end up receiving interventions tested in adult populations but which are not necessarily suitable or effective in the same way for younger people.

The design of the study fits with the research questions, which is focused on acceptability and feasibility. The study as designed should be well placed to answer the research questions and lay the ground work for a later larger trial if progression criteria are met. The inclusion of a process evaluation is also a strength, and this was well described in the study.

I have some minor suggestions which the authors may wish to consider to improve clarity further in some places:-

- The introduction is very comprehensive and detailed. However, I wondered if it might be beneficial to make a more explicit statement that DSE in children are usually transient and can resolve with appropriate and time-limited support, and in most cases will not persist or develop into a more serious mental health difficulty or a specific diagnosis such as psychosis. This is implicit within the introduction, but could benefit from being made a bit more concretely. This is not to diminish the argument that there is unmet need around DSE but more to frame it as needing treatment in its own right rather than it only being of clinical interest because its an early sign of a more serious disorder.

- I think it could be made clearer in the introduction what the underlying theoretical basis of the new intervention is – several different therapy types are named e.g. compassionate approach, but it might be helpful to more explicitly frame the treatment as being under the umbrella of a cognitive-behavioural approach.

- The intervention is well-described, however the authors may wish to refer to the TiDIER reporting tool for interventions which provides a helpful checklist to make sure all relevant information is given in describing the intervention:-

Hoffmann, T. C., Glasziou, P. P., Boutron, I., Milne, R., Perera, R., Moher, D., Altman, D. G., Barbour, V., Macdonald, H., Johnston, M., Lamb, S. E., Dixon-Woods, M., McCulloch, P., Wyatt, J. C., Chan, A. W., & Michie, S. (2014). Better reporting of interventions: template for intervention description and replication (TIDieR) checklist and guide. BMJ, 348, g1687. https://doi.org/10.1136/bmj.g1687

- Method – Consider using a table to display the progression criteria more 'at a glance' with RAG categories (red, amber, green) for each criterion

---

VERSION 1 - AUTHOR RESPONSE

Response to reviewer comments to the author:

- The introduction is very comprehensive and detailed. However, I wondered if it might be beneficial to make a more explicit statement that DSE in children are usually transient and can resolve with appropriate and time-limited support, and in most cases will not persist or develop into a more serious mental health difficulty or a specific diagnosis such as psychosis. This is implicit within the introduction, but could benefit from being made a bit more concretely. This is not to diminish the argument that there is unmet need around DSE but more to frame it as needing treatment in its own right rather than it only being of clinical interest because its an early sign of a more serious disorder.

In response to this recommendation we have included the following sentence in the first paragraph of our Introduction (p. 3):

Voice hearing is often transient in childhood and not usually a sign of severe mental illness, with DSEs (also referred to as multimodal hallucinations) typically less complex and less distressing in non-clinical samples (Medjkane et al., 2020; Skokauskas et al., 2010).

- I think it could be made clearer in the introduction what the underlying theoretical basis of the new intervention is – several different therapy types are named e.g. compassionate

approach, but it might be helpful to more explicitly frame the treatment as being under the umbrella of a cognitive-behavioural approach.

In response to this recommendation we have included the following in our introduction under the section titled ‘The ChUSE trial’ (p.5):

Informed by evidence from CBT interventions for adults and the few studies available that have involved children and young people, the ChUSE therapy integrates psychoeducation and therapeutic mechanisms from CBT and CMT to formulate young people’s experiences of DSEs and identify potential coping strategies to reduce distress and enhancing coping for each young person.

- The intervention is well-described, however the authors may wish to refer to the TiDIER reporting tool for interventions which provides a helpful checklist to make sure all relevant information is given in describing the intervention.

We thank the reviewer for this recommendation and have submitted the completed TiDIER reporting tool as part of our supplementary materials.

- Method – Consider using a table to display the progression criteria more ‘at a glance’ with RAG categories (red, amber, green) for each criterion

We have added this as a Table in the progression criteria section. Please see table below:

Table 4: *Progression Criteria*

**Domain**	**Green**	**Amber**	**Red**
Recruitment	≥3 dyads/month	2 dyads/month	≤1/month (<20 total)
Intervention completion	≥70% complete	50–70%	<50%
Trial retention	≥70% retained	30–70%	<30%
Parent programme uptake	>70% uptake	50–70%	<50%
Safety (SAEs)	<5% SAE	5–10%	>10% SAE

VERSION 2 - REVIEW

**Reviewer**	**1**
**Name**	**Jacobsen, Pamela**
**Affiliation**	**University of Bath Department of Psychology, Psychology**
**Date**	**08-Jun-2026**
**COI**	

The authors have responded well to all the issues raised, and I am happy to recommend publication.
